# Rational Design of CYP3A4 Inhibitors: A One-Atom Linker Elongation in Ritonavir-Like Compounds Leads to a Marked Improvement in the Binding Strength

**DOI:** 10.3390/ijms22020852

**Published:** 2021-01-16

**Authors:** Eric R. Samuels, Irina F. Sevrioukova

**Affiliations:** 1 Department of Pharmaceutical Sciences, University of California, Irvine, CA 92697-3900, USA; samuelse@uci.edu; 2 Department of Molecular Biology and Biochemistry, University of California, Irvine, CA 92697-3900, USA

**Keywords:** CYP3A4, ligand binding, inhibitor design, crystal structure, structure–activity relations, surface mutation

## Abstract

Inhibition of the major human drug-metabolizing cytochrome P450 3A4 (CYP3A4) by pharmaceuticals and other xenobiotics could lead to toxicity, drug–drug interactions and other adverse effects, as well as pharmacoenhancement. Despite serious clinical implications, the structural basis and attributes required for the potent inhibition of CYP3A4 remain to be established. We utilized a rational inhibitor design to investigate the structure–activity relationships in the analogues of ritonavir, the most potent CYP3A4 inhibitor in clinical use. This study elucidated the optimal length of the head-group spacer using eleven (series V) analogues with the R_1_/R_2_ side-groups as phenyls or R_1_–phenyl/R_2_–indole/naphthalene in various stereo configurations. Spectral, functional and structural characterization of the inhibitory complexes showed that a one-atom head-group linker elongation, from pyridyl–ethyl to pyridyl–propyl, was beneficial and markedly improved K_s_, IC_50_ and thermostability of CYP3A4. In contrast, a two-atom linker extension led to a multi-fold decrease in the binding and inhibitory strength, possibly due to spatial and/or conformational constraints. The lead compound, **3h**, was among the best inhibitors designed so far and overall, the strongest binder (K_s_ and IC_50_ of 0.007 and 0.090 µM, respectively). **3h** was the fourth structurally simpler inhibitor superior to ritonavir, which further demonstrates the power of our approach.

## 1. Introduction

**Figure 1:**
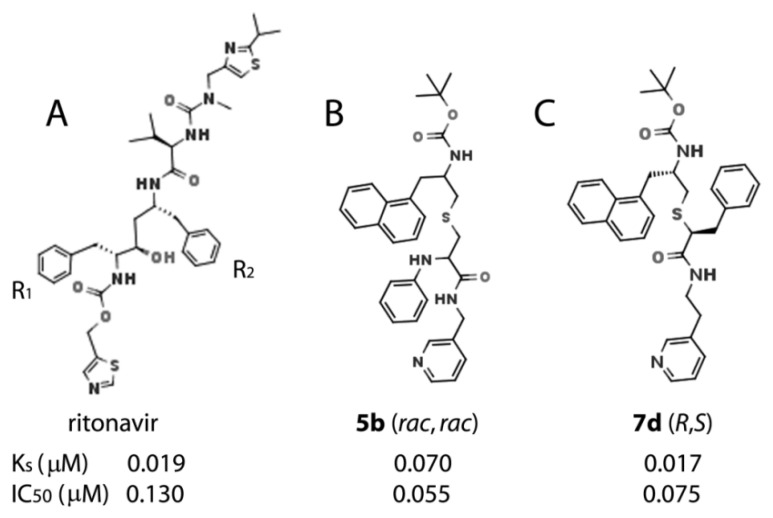
Chemical structures of ritonavir (**A**), a highly potent CYP3A4 inhibitor in clinical use, and the best two rationally designed inhibitors from series IV [10] (**B**,**C**). The R_1_ and R_2_ side-groups are indicated. If the central hydroxyl group of ritonavir is removed, the phenyl side-groups would be in R/R configuration.

Cytochrome P450 (CYP) enzymes are monooxygenases that mediate xenobiotic metabolism and the synthesis of steroids, vitamins and fatty acids [1,2]. In humans, CYP3A4 is the major liver and intestinal P450 isoform with a large and malleable active site which can accommodate chemically diverse compounds. As a result, CYP3A4 clears the majority of administered drugs along with other foreign compounds, such as environmental pollutants, insecticides and pesticides [3,4,5]. Drugs may serve not only as substrates but also as inducers and inhibitors of CYP3A4 [6]. The inhibition of CYP3A4 is usually undesired, because it could lead to drug toxicity, drug–drug interactions and other serious adverse effects. However, when carefully controlled, CYP3A4 inhibition can improve therapeutic efficacy of drugs by slowing down their conversion to more water-soluble, readily excreted metabolites. This beneficial pharmacoenhancing (boosting) effect is currently used for the treatment of HIV infection, where potent CYP3A4 inhibitors, ritonavir (Figure 1A) and its derivative cobicistat, are co-administered with anti-HIV drugs that otherwise are quickly metabolized by CYP3A4 [7,8]. The discovery and utilization of ritonavir as a pharmacoenhancer for HIV-protease inhibitors was a paradigm shift in the treatment of HIV and helped transform the deadly infection into a chronic and manageable disease [9].

Other areas that could benefit from in-depth inhibitory studies on CYP3A4 are the treatment of chronic hepatitis C virus (HCV) infection and the development of CYP3A4-specific index inhibitors. HCV infection is a worldwide health issue with no effective or well tolerated treatments available to date. Through CYP3A4 inhibition, ritonavir is able to diminish the hepatic metabolism of co-administered anti-HCV drugs, enhance response rates, alleviate a pill burden, and provide an additional barrier to the development of resistance-associated variants [11]. The antifungal agent ketoconazole, on the other hand, was widely used as an index inhibitor of CYP3A enzymes in in vivo and in vitro drug–drug interaction studies but, due to high hepatotoxicity, its usage is now limited [12], with ritonavir recommended as the best replacement [13].

However, two major issues associated with ritonavir are off-target activities and poor physicochemical properties [14]. Even though cobicistat has higher solubility, better tolerability and fewer side effects [8,15], it inhibits CYP3A4 2-fold weaker and has similar reactivity toward other drug-metabolizing CYPs [16]. Thus, the inhibitory studies on CYP3A4 could help develop safer and more potent pharmacoenhancers and index inhibitors for in vivo and in vitro studies. Importantly, both ritonavir and cobicistat are chemically complex molecules that were not developed based on the 3D structure of CYP3A4. Moreover, ritonavir was originally designed as an HIV protease inhibitor, whose ability to potently inhibit CYP3A4 was purely coincidental [7]. Therefore, we undertook a series of studies to investigate what structural determinants are needed for potent inhibition and whether the inhibitory potency of ritonavir, the most potent CYP3A4 inhibitor in clinical use, could be further improved via rational structure-based inhibitor design. Detailed investigations on the structure–activity relationship (SAR) of ritonavir analogues and their interaction with CYP3A4 are also important from the basic science perspective, as they could clarify the inhibitory/ligand binding mechanism and provide deeper insight into the plasticity and adaptability of CYP3A4.

Our earlier studies on the analogues of desoxyritonavir (provided by Gilead Sciences) demonstrated that the association of ritonavir-like molecules is driven by heme coordination [17], identified pyridine as the strongest heme-ligating group [18] and laid the groundwork for the development of a pharmacophore model for a potent CYP3A4 inhibitor [19]. In the following studies [10,20,21,22], we utilized a build-from-scratch approach to evaluate the importance of other pharmacophoric determinants, such as the backbone length/composition and the size/hydrophobicity of the R_1_ and R_2_ side-groups, represented by phenyls in ritonavir (Figure 1A). We found that the binding affinity and inhibitory potency of ritonavir analogues depend on the distance between the functional groups, H-bonding to the active site S119, and the side-group hydrophobicity and stereochemistry. One particular observation was that a one-carbon linker extension, from pyridyl–methyl to pyridyl–ethyl, and/or phenyl>indole>naphthalene substitution in R_2_ largely improve the binding affinity and IC_50_ [10,22]. The most potent inhibitors designed so far, **5b** and **7d** (series IV) [10], are shown in Figure 1B,C.

**Figure 2:**
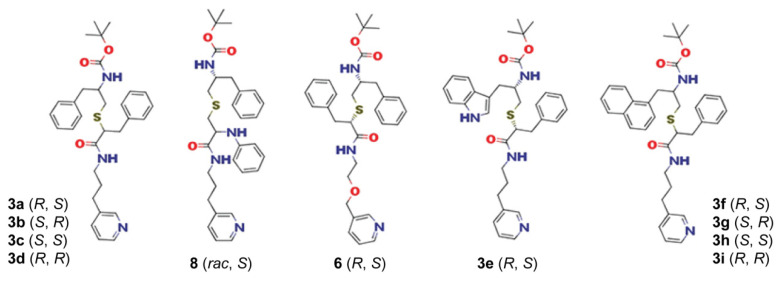
Chemical structures of series V analogues.

The current study was set to test if further pyridyl–linker extension would be beneficial or not. For this purpose, we synthesized eleven (series V) compounds with the pyridyl–propyl or pyridyl–methoxyethyl (butyl-like) spacer, R_1_–phenyl, and R_2_–phenyl/indole/naphthalene in various stereo configurations (Figure 2). It was found that one- but not two-atom linker extension leads to tighter binding and more potent inhibition of CYP3A4. Crystal structures of the inhibitory complexes showed that the elongated compounds adopt a more relaxed conformation, which enables stronger N-pyridine ligation to the heme. There was also a downside effect—an increase in ligand mobility in the active site. A comparative analysis of series IV and V analogues suggests that further improvement in the inhibitory power could be achieved by balancing the flexibility/adjustability and rigidity of the scaffold.

## 2. Results

### 2.1. Rationale for Series V Analogues

While working on series III inhibitors [22], we found that a one-atom elongation of the spacer between the heme-ligating pyridine and the R_1_ side-group, from five- to six-atom separation, improves the binding affinity and inhibitory potency for CYP3A4. Studies on series IV analogues [10], in turn, showed that the size/hydrophobicity of R_2_ is another factor critical for the binding and inhibitory strength, which could be modulated via R_1_/R_2_ stereochemistry. To further investigate the SAR of ritonavir-like inhibitors, we designed eleven (series V) analogues to test whether the further extension of the pyridyl-linker by one or two atoms would be beneficial or not. The first subseries, **3a**–**d** and **8**, had a pyridyl–propyl linker and R_1_/R_2_–phenyls in different stereo configuration (Figure 2). Compound **8** (*rac*, *S*) was included to test the effect of a longer R_1_/R_2_ spacer (five vs. four atoms for other analogues). Compound **6** (*R*, *S*) also contains R_1_/R_2_–phenyls and an extended pyridyl–methoxyethyl (butyl-like) linker. The second pyridyl–propyl subseries was designed to assess the impact of a bulkier R_2_, represented by indole in **3e** (*R*, *S*) and naphthalene in **3f**–**i** in various stereo configurations. Compounds **6** and **3e** were synthesized in the R/S configuration because it is more preferable for the binding to CYP3A4 [10]. As previously, spectral titrations, inhibitory and temperature denaturation assays, kinetic measurements, and co-crystallization of analogues with CYP3A4 were conducted for SAR evaluation.

### 2.2. The ***3a**–**d*** Analogues

#### 2.2.1. Spectral and Biochemical Properties of **3a**–**d**

**Figure 3:**
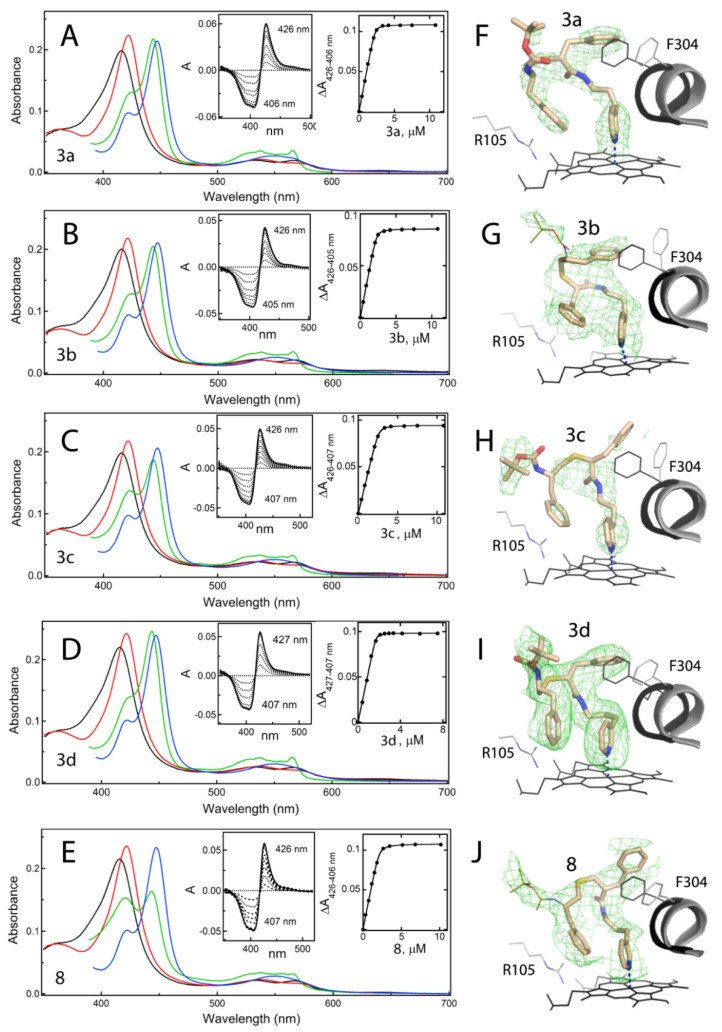
Spectral properties and the binding modes of **3a**–**d** and **8**. (**A**–**E**) Spectral changes induced in cytochrome P450 (CYP) by **3a**–**d** and **8**. Absorbance spectra of ferric ligand-free and inhibitor-bound CYP3A4 are in black and red, respectively. Spectra of ferrous ligand-bound CYP3A4 and its CO-adduct are in green and blue, respectively. Left insets are difference spectra recorded during equilibrium titrations; right insets are titration plots with quadratic fittings. The derived spectral dissociation constants (K_s_) are given in Table 1. (**F**–**J**) The binding modes of **3a**–**d** and **8** observed in the crystal structures. Carbon, oxygen, nitrogen and sulphur atoms are depicted in beige, red, blue and yellow, respectively. The *R*/*S* conformer of **8** (*rac*, *S*) was selectively co-crystallized with CYP3A4. The adjacent I-helix and F304 in the inhibitory complexes and water-bound CYP3A4 (5VCC structure) are depicted in gray and black, respectively. Polder omit maps are contoured at 3σ level and shown as green mesh. Tail-groups of **3b** and **8** that could not be traced in the electron density maps are shown as thin lines.

Compared to the pyridyl–ethyl containing **4e**–**h** (series III analogues) [22], there was a marked improvement in the binding and inhibitory properties of **3a**–**d**. Except for **3b** (*R*, *S*), all compounds induced a larger red shift in the Soret band (type II spectral change; Figure 3A–D), with λ_max_ for the ferric and ferrous CYP3A4 at 422 and 444 nm, respectively, vs. 421 and 443 nm for **4e**–**h**. Two other spectral parameters reflecting the strength of N-pyridine ligation, A_417/422nm_ (ratio between the absorbance maxima in the absolute ligand-free/bound spectra) and ΔA_max_ (peak/trough amplitude in the difference spectra; left insets in Figure 3A–D), were the highest observed to date (Table 1). Spectral dissociation constants (K_s_, measure of the binding affinity) were derived from titration plots (right insets in Figure 3A–D). Compared to **4e**–**h**, the binding affinity of **3a**–**d** was 2-to-3-fold higher: K_s_ of 0.013–0.029 μM vs. 0.040–0.055 μM, respectively. The IC_50_ values for the 7-benzyloxy-4-(trifluoromethyl)coumarin (BFC) debenzylase activity of CYP3A4 correlated with K_s_ and decreased by 1.5-to-5-fold upon linker extension (Table 1). The lowest IC_50_ was derived for **3a** (0.16 μM). Another notable improvement was in thermostability of ligand-bound CYP3A4, whose melting temperature (T_m_) increased by 5.3–8.1 °C upon **3a**–**d** ligation. Again, the highest ΔT_m_ was observed for the CYP3A4–**3a** complex. For comparison, ΔT_m_ for **4e**–**h** did not exceed 4.7 °C [22]. The ligand binding rate (*k*_fast_ in Table 1) was not affected by linker modification and remained within the 9.5–12.5 s^−1^ range.

#### 2.2.2. Crystallization of **3a**–**d**-Bound CYP3A4

All compounds willingly co-crystallized with CYP3A4 in the I222 crystal form, which has one molecule per asymmetric unit. Ligand fitting for **3b** and **3c** was relatively straightforward. The **3a** and **3d** binding modes, however, could not be accurately determined due to partially discontinuous electron density, likely the result of thermal disorder. The wild-type (WT) CYP3A4–**3a** complex was re-crystallized in the C2 space group, where one of the two molecules in the asymmetric unit (molecule A) was well defined. In the C2 crystal lattice, the F–F’ fragment mediates intermolecular contacts and undergoes positional and conformational rearrangement (Figure S1A–C). Nonetheless, these changes had no significant effect on the ligand conformation (Figure S2). With **3d**, neither WT nor the previously used K282A/K285A mutant [20] produced crystals in space groups other than I222. Therefore, we replaced with alanine two other high-entropy surface residues, K421 and K424 [20], located in the loop region on the distal face of CYP3A4. The K421A/K424A mutant was well expressed, could bind **3d** equally tight (K_s_ of 0.019 vs. 0.013 μM for WT; Figure S3), and produced the desired C2 crystals. Importantly, K421 and K424 do not form specific interactions with residues of the same or nearby molecules, and their substitution with alanine does not alter the 400–430 fragment conformation (Figure S1D).

#### 2.2.3. Compounds **3a**–**d** Binding Modes

**Figure 4:**
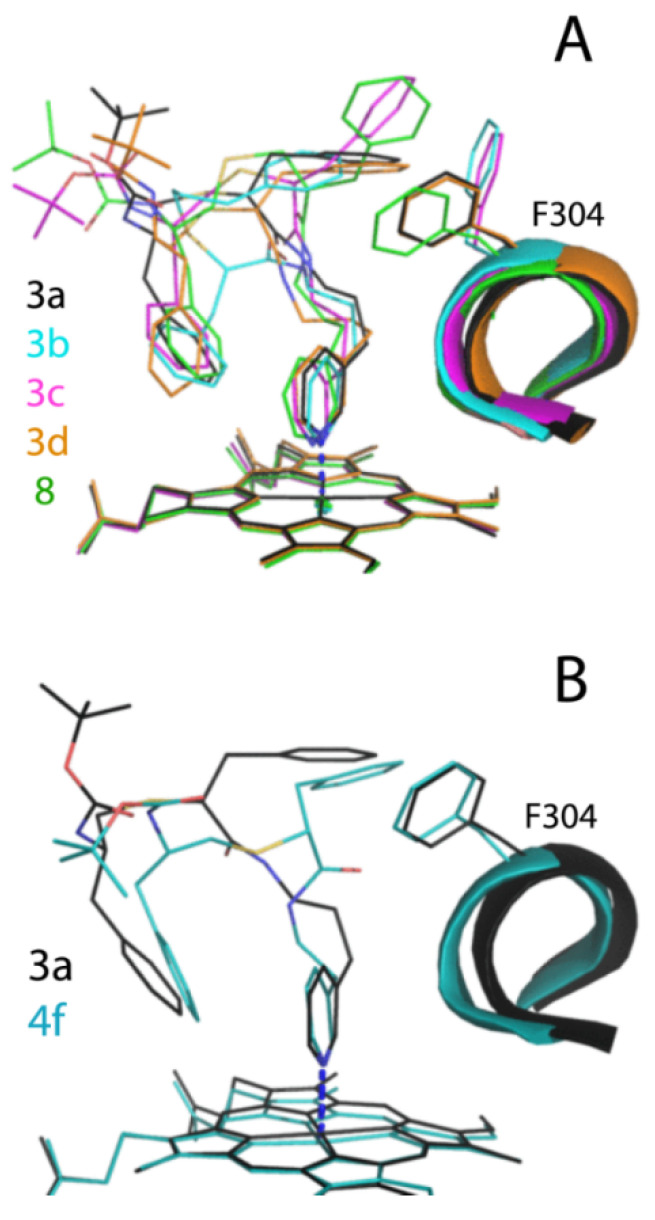
(**A**) Structural overlay of **3a**–**d-** and **8**-bound CYP3A4. Unlike other compounds, **3b** (*S*, *R*) has a less favourable reverse side-group orientation, with R_2_–phenyl above the I-helix (P1 site) and R_2_–phenyl near the heme-ligating pyridine (P2 site). The disordered end-moiety of **3b** is not shown. (**B**) Comparison of isosteric **3a** and series IV **4f** analogue [10] demonstrates how one-atom linker elongation impacts the ligand binding mode. The Boc-group of **4f** is disordered and shown in an arbitrary conformation.

Structures of CYP3A4 bound to **3a**–**d** were solved to 2.55–2.8 Å resolution (Table S1). In these and other structures reported here, ligands were fit into polder omit maps, the validity of which was verified (Table S3) [23]. Only the well defined molecules A of **3a**- and **3d**-bound complexes were used for structural comparison, summarized in Table 2. Ligand positioning and orientation relative to the central I-helix, as well as polder omit maps, are shown in Figure 3F–I. Compounds **3a**, **3c** and **3d** bind in a traditional orientation, with the R_1_–phenyl embedded into a hydrophobic pocket above the I-helix (P1 site) and R_2_–phenyl near the heme-ligating pyridine (P2 site). In contrast, **3d** ligates to CYP3A4 in a reverse orientation, with R_1_ and R_2_ at the P2 and P1 sites, respectively (Figure 3G), just like its methyl- and ethyl-linker counterparts [22]. The *S*/*R* conformation is less favourable for the binding to CYP3A4, as **3d** forms the longest Fe–N bond and the weakest H-bond with the active site S119, a prerequisite for potent inhibition [21]. This translates to the lowest binding affinity, inhibitory potency, and stabilizing effect of **3d** (Table 1). Even so, superposition of **3a**–**d** shows that, regardless of the side-group stereochemistry, phenyls at the P2 site virtually coincide (Figure 4A). Being equally close to the heme and pyridine (~3.8–4.2 Å), the side-groups are optimally poised for hydrophobic and aromatic interactions. The nearby R105 side chain (displayed in Figure 3F–J) further stabilizes the inhibitory complexes by promoting π–cation interactions with the aromatic R_2_. Comparison of the subseries lead compound, **3a**, with the shorter **4f** stereoisomer [22], demonstrates how a one-atom linker extension alters the ligand binding mode (Figure 4B). The longer pyridine–R_1_ spacer allows **3a** to adopt a more relaxed conformation, with a larger tilt toward the heme. This strengthens the Fe–N bond (2.1 vs. 2.3 Å in **4f**) and increases the overlap with the heme macrocycle. Additional stabilization is provided by the Boc-group, mediating multiple van der Waals interactions but disordered in **4f**.

### 2.3. R_1_/R_2_ Spacer Extension Has No Beneficial Effect

Compound **8** (*rac*, *S*) resembles **3a**–**d** but has a one-atom longer R_1_–R_2_ spacer. Spectral, biochemical and kinetic properties of **8** are mostly similar (Table 1). The only distinction was a slightly lower ΔT_m_ (5.0 °C) and incomplete heme reduction in the inhibitory complex: ~60% vs. ≥80% for **3a**–**d** (compare green spectra in Figure 3A–E). In the crystal structure, **8** ligates to the heme in a traditional orientation (Figure 3J). Importantly, the stereochemistry of racemic R_1_–phenyl was not specified during the first refining cycle but it was assigned as *R* by the structure refinement program. The *R*/*S* configuration was later confirmed to provide a better fit. That the five-atom R_1_–R_2_ spacer is less optimal for the binding to CYP3A4 is evident from the inability of **8** to strongly H-bond with S119 (Table 2). Moreover, the latter interaction involves the amide nitrogen rather than carbonyl oxygen, recruited in other analogues. Thus, the R_1_/R_2_ spacer extension has no beneficial effect.

### 2.4. Two-Atom Pyridyl-Linker Elongation Is Detrimental for the Binding to CYP3A4

#### 2.4.1. Synthetic Approaches for Linker Elongation

To further investigate the correlation between length/flexibility and binding affinity/inhibitory potency, a butyl-like linker was evaluated. For preliminary examination, a methoxyethyl linkage within the starting material was chosen as a bioisostere. Attempts to synthesize a starting material with either pyridine-3-methanol and 2-(Boc-amino)ethyl bromide [24] or 3-picolyl chloride [25] and Boc-ethanolamine, under various conditions, were either unsuccessful or produced product in very low yield. Moreover, the 3-picolyl chloride starting material was recovered from the synthesis, suggesting either low reactivity of chloride in this substitution, or diminished activity due to the insolubility of the hydrochloride salt starting material. Because ether formation with the Boc-protected amino starting material was problematic, bromoacetonitrile was utilized instead. The nitrile product **4** (Scheme 2 in Materials and Methods section) was formed in more suitable yield. Several reduction methods were then attempted to reduce the nitrile to the amine. Reduction using LiAlH_4_ under various conditions [26] was unsuccessful, as was electron transfer reduction via samarium (II) iodide [27]. H_2_/Raney Nickel with NH_4_OH [28] over-reduced the product to piperidine and attempts to control reduction by shortening the reaction time [29] were ineffective. Transition metal borides formed in situ were then employed. Zirconium boride, formed in situ from ZrCl_4_ and NaBH_4_ has been previously shown to reduce nitriles to amines [30], but was unsuccessful. However, nickel boride (formed in situ from NiCl_2_ and NaBH_4_) with Boc-anhydride as a trapping agent [31], formed the Boc-protected amino product **5**. Although catalytic quantities of nickel chloride have been shown to reduce nitriles to amines [32], the reaction was more successful with stoichiometric amounts of nickel chloride hexahydrate and nitrile [31,33]. Overreduction to the piperidine product did occur, however, it was successfully separated by column chromatography. Deprotection of Boc with trifluoroacetic acid resulted in free amine, which was then coupled with **2a** to form the final methox–ethyl, butyl-like product, **6**.

#### 2.4.2. Properties of **6**

**Figure 5:**
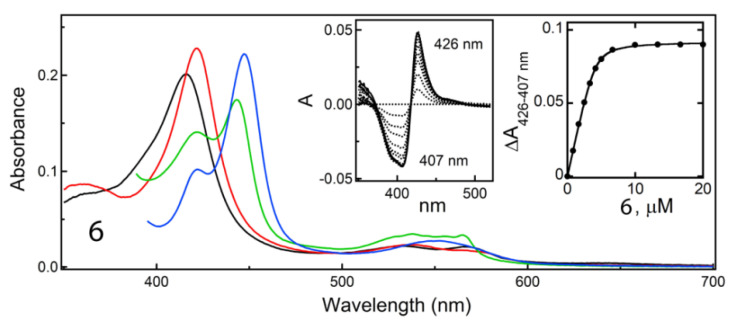
Spectral changes in CYP3A4 induced by compound **6** binding. Absorbance spectra of ferric ligand-free and inhibitor-bound CYP3A4 are in black and red, respectively. Spectra of ferrous ligand-bound CYP3A4 and its CO-adduct are in green and blue, respectively. Left inset shows difference in the spectra recorded during equilibrium titrations; right inset is a titration plot with quadratic fitting. The derived K_s_ value is given in Table 1. It was not possible to determine the inhibitory complex structure because **6** dissociated from CYP3A4 during crystallization.

Similar to other analogues, compound **6** (*R*, *S*) acted as a type II heme ligand and induced a red shift in the Soret band (Figure 5). Nonetheless, **6** was the weakest binder and the least potent inhibitor in the entire series, with K_s_ and IC_50_ increased by multi-fold (Table 1). The fact that **6** ligates weakly to CYP3A4 is also evident from the lowest ΔT_m_ (<3 °C). Moreover, we could not co-crystallize CYP3A4 with **6** because it dissociated during crystallization. The inability of **6** to strongly ligate to the heme could be due to spatial limitations in the active site and/or conformational constraints imposed by the lengthy linker. Therefore, we did not pursue any further modification of the pyridine-R_1_ spacer and instead focused on a more detailed investigation of the pyridyl–propyl scaffold by replacing R_2_–phenyl with the larger and more hydrophobic indole and naphthalene.

### 2.5. The ***3e**–**i*** Subseries

#### 2.5.1. Spectral and Biochemical Properties of **3e**–**i**

**Figure 6:**
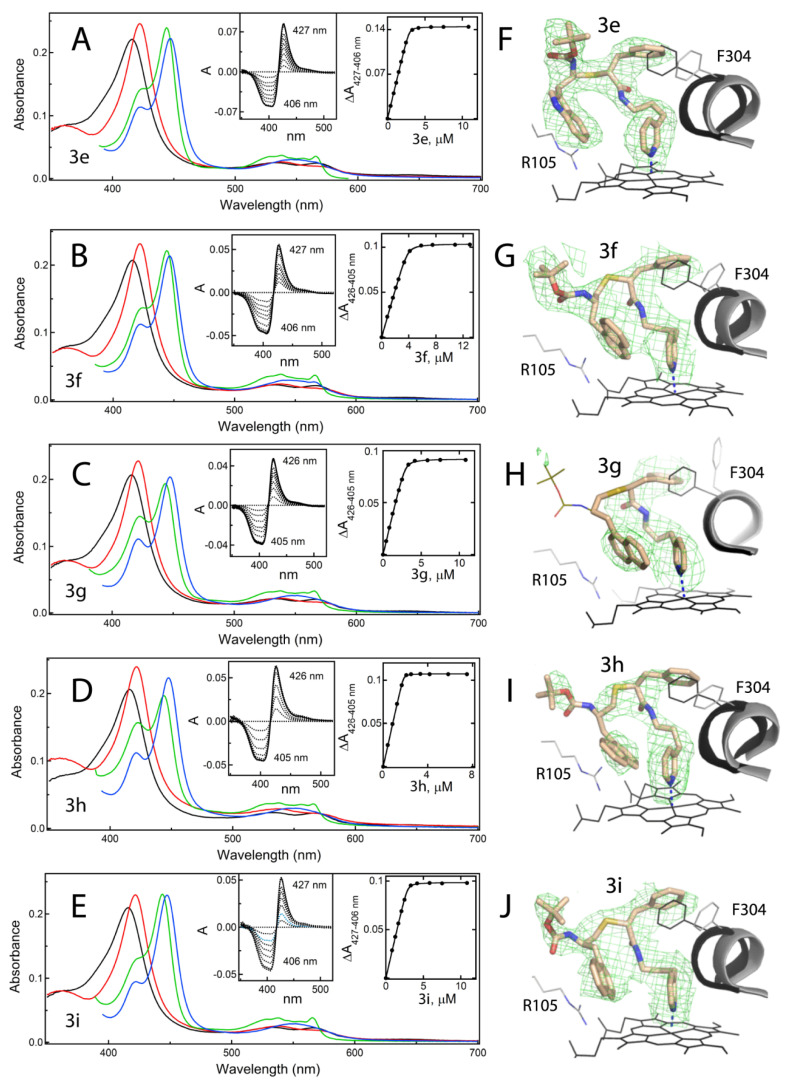
Spectral properties and binding orientations of **3e**–**i**. (**A**–**E**) Spectral changes induced in CYP3A4 by **3e**–**i**. Absorbance spectra of ferric ligand-free and inhibitor-bound CYP3A4 are in black and red, respectively. Spectra of ferrous ligand-bound CYP3A4 and its CO-adduct are in green and blue, respectively. Left insets are the difference spectra recorded during equilibrium titrations; right insets are titration plots with quadratic fittings. The derived K_s_ values are listed in Table 1. (**F**–**J**) The binding modes of **3e**–**i** observed in the crystal structures. Carbon, oxygen, nitrogen and sulphur atoms are shown in beige, red, blue and yellow, respectively. The adjacent I-helix and F304 in the inhibitory complexes and water-bound CYP3A4 (5VCC structure) are depicted in gray and black, respectively. Polder omit maps contoured at 3σ level are shown as green mesh. The disordered tail of **3g** is shown as thin lines.

Absolute and difference absorbance spectra and titration plots for the **3e**–**i** subseries are shown in Figure 6A–E. Compound **3e** (*R*, *S*) was the only R_2_-indole containing analogue investigated and, along with **3f** (*R*, *S*) and **3i** (*R*, *R*), was among the strongest binders and inhibitors of CYP3A4: K_s_ and IC_50_ of 0.0015–0.019 μM and 0.15–0.016 μM, respectively. However, none of these compounds were superior to the R_2_–phenyl containing **3a** (Table 1). The comparison between **3e** and **3f** with the shorter pyridyl-ethyl counterparts (**7b** and **7d** from series IV [10], respectively) showed that significant improvement in K_s_ was achieved only for **3e** (4-fold decrease), while the IC_50_ value and both parameters for **3f** were either higher or unchanged. As expected, the S/R conformer, **3g**, was the weakest inhibitor in the subseries and similar to **3b** (Table 1). **3h** (*S*, *S*), in turn, was the series V lead compound: K_S_ and IC_50_ of 0.007 and 0.090 µM, respectively. This is a 3-fold improvement relative to the R_1_/R_2_–phenyl containing stereoisomer **3c**. Thus, the impact of the phenyl-to-naphthalene replacement in R_2_ depends on the side-group stereochemistry and is more pronounced when configuration is *S*/*S*.

#### 2.5.2. The **3e**–**i** Binding Modes

All compounds could be co-crystallized with WT CYP3A4 either in I222, C2 or I2 space groups (Table S2). Similar to C2, the I2 space group has two molecules in the asymmetric unit, where only molecule A is well defined. Compound **3i** was poorly seen in the I222 structure and did not form C2 crystals with WT CYP3A4. Therefore, like **3d**, this analogue was co-crystallized with the K421A/K424A mutant. The ligand binding modes and structural features are compared in Figure 6F–J and Table 2. One distinction of this subseries is that all stereoisomers, including **3g**, ligate to the heme in a traditional orientation, with R_1_–phenyl in the P1 pocket and R_2_-indole/naphthalene at P2 site. Still, even in this orientation, **3g** has the longest Fe–N bond, cannot H-bond to S119, and has no R_1_/F304 overlap. This is in contrast to four other compounds, which strongly ligate to the heme, form strong H-bonds with S119, and maximize the R_1_/F304 and R_2_/pyridine overlap (Table 2).

**Figure 7:**
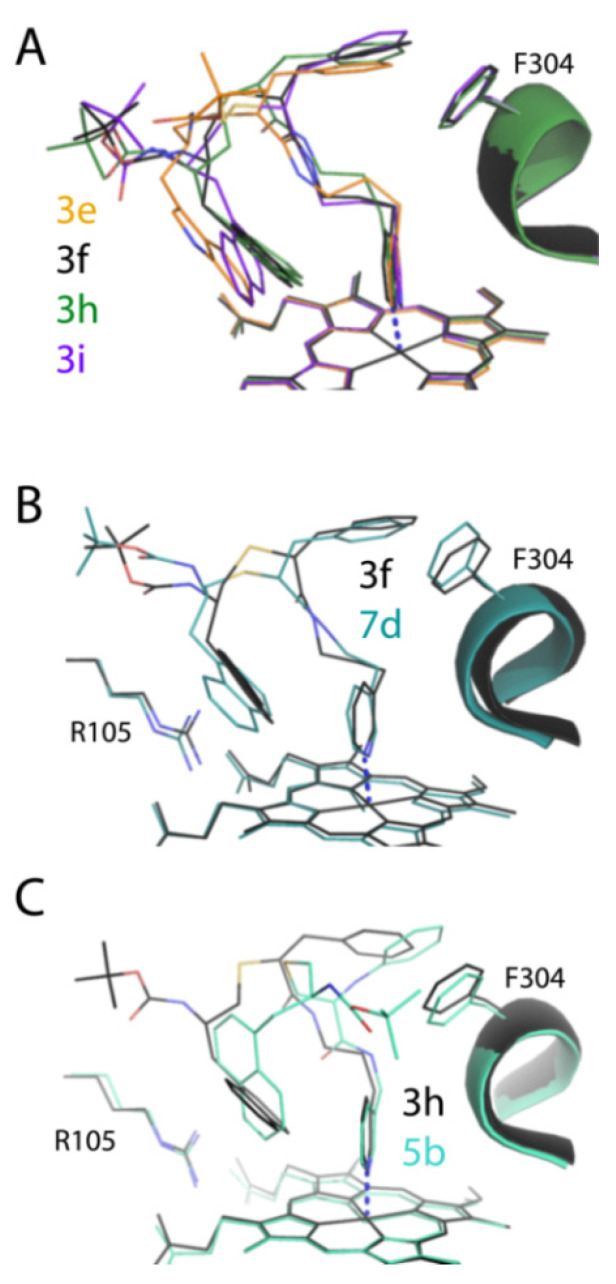
(**A**) Structural overlay of the **3e**–**i**-bound complexes. Unlike **3b** (*S*, *R*) (Figure 4A), **3f** (*S*, *R*) binds in a traditional orientation. However, **3f** forms the longest Fe–N bond and cannot establish an H-bond with the active site S119 (Table 2), meaning that the R/S configuration is still less favourable. (**B**) Superposition of isosteric **7d** (series IV analogue [10]) and **3f** shows a small impact of one-atom linker elongation on the ligand binding mode. (**C**) Comparison of the best series IV and V inhibitors, **5b** [10] and **3h**. The pyridyl–ethyl containing **5b** has a five- rather than four-atom R_1_–R_2_ spacer (Figure 1B) and adopts a distinct conformation in the active site: R_1_–phenyl protrudes deeper into the P1 pocket, R_2_-naphthalene is perpendicular rather than parallel to the heme, and the terminal Boc-group points away rather than toward the substrate channel.

Superposition of **3e**, **3f**, **3h** and **3i** shows similar backbone curvatures, coinciding with R_1_–phenyls, and the R_2_-groups equally distant from the heme but not the pyridine moiety (Figure 7A). In **3e**, the R_2_-indole is oriented vertically rather than horizontally, as naphthalene rings do, possibly due to its more hydrophilic nature. Since **3f** and **3h** bind in a similar manner, it was not possible to deduce based on structural and experimental data why the latter analogue inhibits CYP3A4 twice as stronger. Comparison of **3f** with the shorter and isosteric **7d** (Figure 7B) also could not explain the 2-fold difference in IC_50_, as the inhibitors’ head-, side- and tail-groups were positioned similarly. On the other hand, **5b** (*S*, *S*), selectively chosen by CYP3A4 from racemic mixture [10], and the isosteric and equally potent **3h** have notably distinct conformations (Figure 7C): the **5b** R_1_ inserts deeper in the P1 pocket, R_2_ orients vertically to the heme, and the end-moiety points away rather than toward the substrate channel. Since it became challenging to rank the high affinity analogues based on traditional approaches, we introduced an additional assay for inhibitor characterization.

### 2.6. H_2_O_2_ Heme Accessibility Assessment

**Figure 8:**
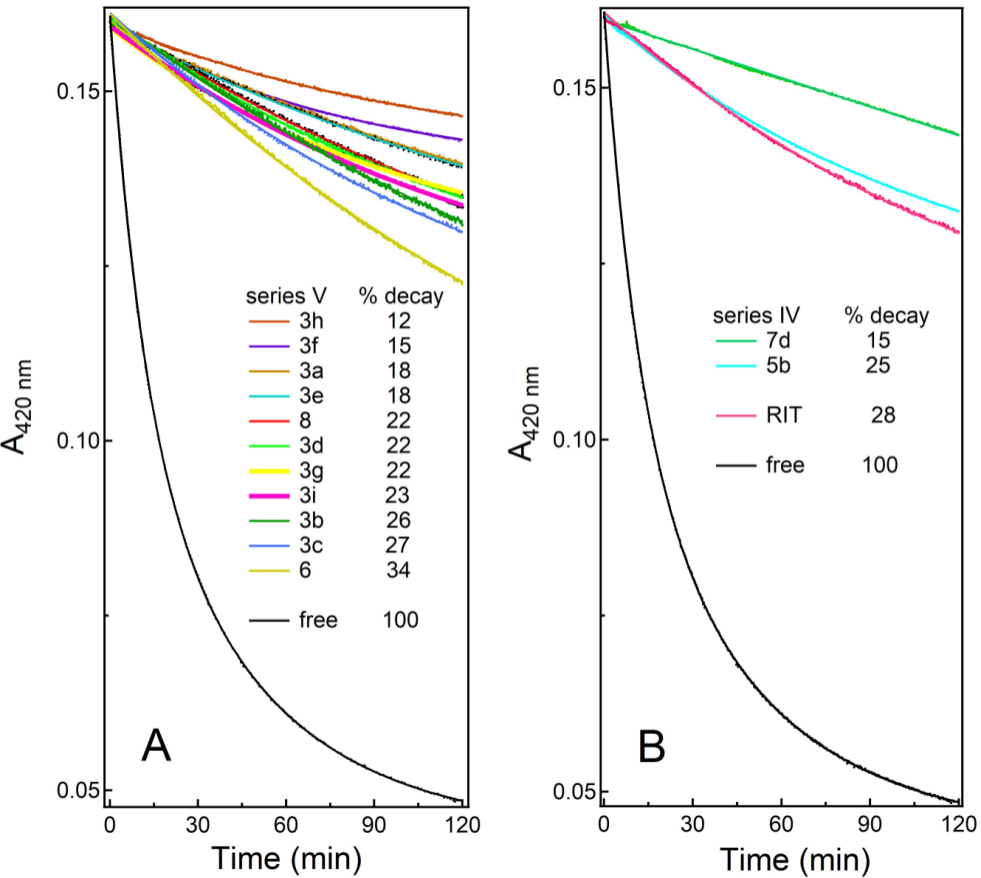
H_2_O_2_-dependent heme depletion in ligand-free and inhibitor-bound CYP3A4. (**A**) Effect of series V inhibitors on the heme accessibility to H_2_O_2_. Overall, the inhibitor binding significantly protected the cofactor, and the amount of heme destroyed correlated with the K_s_/IC_50_ values. (**B**) A comparative assay for CYP3A4 bound to ritonavir and the best series IV inhibitors, **5b** and **7d**. The heme accessibility was the lowest in CYP3A4 ligated to the high affinity/potency inhibitors, **3h** and **7d**.

Ritonavir-like molecules inhibit CYP3A4 not only via heme ligation and decrease in the heme redox potential [34] but also by blocking the active site and preventing substrates from accessing the catalytic centre. We usually evaluate the heme accessibility by measuring the rate and percentage of anaerobic heme reduction by sodium dithionite, an artificial electron donor (*k*^ET^ in Table 1). However, *k*^ET^ has a narrow spread, and the portion of heme reduced does not correlate with IC_50_ (Table 1). Therefore, we utilized another method, heme bleaching with hydrogen peroxide, to probe the accessibility of the cofactor. H_2_O_2_ is a small oxidizing agent that easily penetrates the protein interior and destroys the heme without altering the P450 structure [35]. Kinetic plots for series V inhibitors are shown in Figure 8A. Due to the complex, multiphasic nature of heme decay kinetics, it was more convenient to compare the percentage of heme destroyed at the end of the reaction. In general, the strongest binders/inhibitors (**3h**, **3f**, **3a** and **3e**) preserved most of the heme (>80%), whereas the weaker ligands (**3g**, **3b**, **3c** and **6**) had a smaller protecting effect (4–16% higher heme loss). To further test the applicability of the assay, we measured H_2_O_2_-dependent heme destruction in CYP3A4 bound to ritonavir and series IV analogues **5b** and **7d** (Figure 1). As seen from Figure 8B, only the R_2_–naphthalene containing **7d** protected the heme as well as the best series V inhibitors. Interestingly, the fraction of heme destroyed seemed to better correlate with the K_s_/IC_50_ pair than the individual parameters. Either way, the heme depletion assay was informative and suggests that, along with the strong heme-ligating moiety, the R_2_-mediated interactions at the P2 site could control/minimize heme accessibility.

## 3. Discussion

This work was a continuation of our efforts to identify structural attributes required for potent inhibition of human drug-metabolizing CYP3A4, the major and most clinically relevant P450 isoform. This knowledge is needed for a better understanding of the CYP3A4 inhibitory/ligand binding mechanism and building a pharmacophore model for a potent CYP3A4 inhibitor. The identification of an ensemble of electronic and steric features leading to potent CYP3A4 inhibition could help develop safer drugs and more effective pharmacoenhancers. Ritonavir, originally designed as an anti-HIV drug [36], remains the most potent CYP3A4 inhibitor in clinical use [37] and serves as a ‘gold standard’ in our studies. Based on the SAR analysis of the closest analogues of ritonavir, we constructed the first structure-based pharmacophore model for a potent CYP3A4-specific inhibitor [19] and tested some of the determinants, including the backbone length/composition, R_1_ and R_2_ substituents, and pyridine–R_1_ and R_1_–R_2_ spacers [10,20,21,22]. This study builds on our prior work and was performed to elucidate the optimal length of the pyridine–R_1_ linker.

The linker extension from pyridyl–ethyl to pyridyl–propyl (six- to seven-atom pyridine–R_1_ separation) was found to be more optimal for the R_1_/R_2_–phenyl containing compounds, **3a**–**d**, as it increased both the binding and inhibitory strength by several-fold (Table 1). For the R_1_–phenyl/R_2_-indole/naphthalene analogues, **3e**–**i**, the impact was not uniform and depended on the side-group stereochemistry. In accordance with the previous findings [10,22], we confirmed that the *S*/*R* configuration was the least favourable, because it forces analogues to bind in reverse or sub-optimal orientations. In contrast, regardless of the scaffold, *R*/*S* was the most preferable conformation and, once again, was selected by CYP3A4 during co-crystallization with **8** (*rac*, *S*). The series V front-runner, however, was **3h** (*S*, *S*). This analogue has outstandingly low K_s_ (0.007 μM) and inhibits CYP3A4 nearly as potently as the prior lead compounds (IC_50_ of 0.090 μM vs. 0.055–0.070 μM for **5b** and **7d**) and 1.5-fold stronger than ritonavir (K_s_ and IC_50_ of 0.019 and 0.130 μM, respectively).

The currently prevalent notion is that ritonavir acts as a mechanism-based inhibitor by producing reactive metabolite(s) inactivating CYP3A4 via covalent attachment [38,39,40,41,42]. In our experimental system, we could not detect time-dependent CYP3A4 inactivation upon preincubation with NADPH and, instead, observed a slight decrease in IC_50_ for ritonavir, likely due to its partial metabolism [22]. Whether the membrane bilayer, cytochrome b_5_ and/or other microsomal proteins are needed to mediate the reactive species production remains to be established. However, this and our prior studies demonstrate that the key factors contributing to the inhibition of CYP3A4 are the tight heme binding and blockage of the active site.

One problem with series V compounds was that, aside from the best and the worst inhibitors, the ranking was difficult due to low spread in IC_50_ (Table 1). By definition, IC_50_ is the half inhibitory concentration, which under our experimental conditions (0.1 μM CYP3A4; 1:1 enzyme–inhibitor complex) is limited to 0.05 μM. This explains the IC_50_ clustering and predicts a similar scenario for the next generation of optimized compounds. Contrarily, K_s_ does not depend on protein concentration and, as demonstrated for **3h**, its significant improvement can still be achieved. The newly introduced assay, H_2_O_2_-dependent heme bleaching, helped to better evaluate the heme accessibility in the inhibitor-bound CYP3A4 and demonstrated that, compared to the best series IV and V analogues, the protective effect of ritonavir was considerably lower. This further underlines the importance of strong heme ligation and the fuller occupancy of the P2 site.

Another challenge was to accurately define the ligand binding modes. Despite the fact that most of the new compounds were the tight binders that willingly co-crystallized with CYP3A4, it was difficult to determine their binding orientation in traditional I222 crystals, containing one loosely packed CYP3A4 molecule in the unit cell. This and the higher motional freedom of elongated compounds led to thermal disorder and discontinuous electron density maps. To overcome this problem, we recrystallized some but not all inhibitory complexes in the more densely packed C2 and I2 space groups. As expected, changes in crystal packing triggered the restructuring of surface loops, including the F–F’ fragment (Figure S1A–C), which nonetheless only minimally distorted the ligand binding modes (Figure S2). Two compounds, **3d** and **3i**, failed to co-crystallize with WT and the previously obtained K282A/K285A CYP3A4 [20] in the desired crystal forms. This prompted us to clone another surface mutant, K421A/K424A, which had similar spectral and ligand binding properties (Figure S3), easily formed C2 crystals, and maintained the same structural fold (Figure S1D). Thus, along with WT and K282A/K285A CYP3A4, this variant can be used in crystallization trials to increase chances for success.

**Figure 9:**
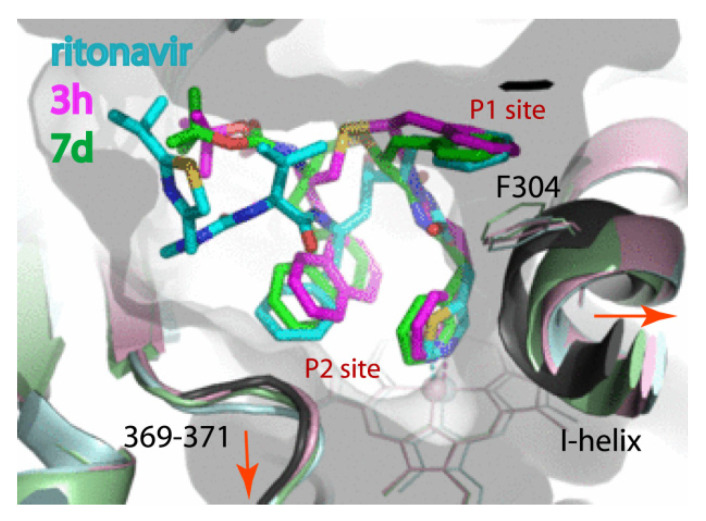
Relative orientation of the best rationally designed inhibitors, **3h** and **7d**, and ritonavir (3NXU structure; molecule A). The respective structures are coloured in pink, light green and light cyan. Structure of water bound CYP3A4 (5VCC) is rendered in black. Displacement of the I-helix and the 369–371 fragment triggered by the inhibitor binding is indicated by red arrows.

Determination of all but **6**-bound structures of CYP3A4 was important because it enabled a structural comparison within/between subseries and with the pyridyl–ethyl counterparts (Figure 4B and Figure 7B,C). Comparative analysis suggests that the positive impact of one-atom linker extension on K_s_ and IC_50_ could be due to the higher flexibility and adjustability of the elongated analogues, leading to stronger heme ligation and a more relaxed fit. This, in turn, strengthens the protein–ligand interactions mediated by the side-groups, as manifested by a large increase in thermostability of CYP3A4 (ΔT_m_ in Table 1). There was also a downside effect, an increase in ligand mobility and thermal motion (evident from the elevated B-factors; Tables S1 and S2), which could lower the inhibitory potency to some extent. This possibility explains why most series V analogues are inferior to the shorter **5b** and **7d**. Thus, a balance needs to be found between the flexibility/adjustability and rigidity of the scaffold. One way to achieve this would be through modification of the end-moiety, currently presented by a simple amino-protecting Boc-group. Due to a spacious active site and the adjacent substrate access channel (Figure 9), the addition/optimization of the end-moiety might not only improve the inhibitory potency of the analogues, but also address the solubility and cross-reactivity issues associated with ritonavir.

Since the end-moiety has yet to be evaluated and optimized, it would be premature to draw any final conclusions on the rational inhibitor design. However, this study completes the investigation of the most important part of the ritonavir-like scaffold—the heme-ligating, side-group containing inhibitor core. A build-from-scratch approach utilized for inhibitor design, with systematic changing/testing of every pharmacophoric determinant, and the availability of structural information on the inhibitory complexes were critical for deciphering the relative importance of the structural elements of ritonavir. SAR analyses of the rationally designed analogues not only confirmed the early observations [19], but also led to new discoveries and conclusions. In particular, all investigated compounds were found to cause the I-helix distortion, with the largest shift (>2.0 Å) observed in CYP3A4 complexed with strong binders/inhibitors, including ritonavir (Figure 9). Such high plasticity of the I-helix enables the R_1_–phenyl to insert deeper into P1 pocket, with no apparent penalty for the conformational constraint. However, a 2-fold drop in IC_50_ observed upon the R_1_–phenyl-to-naphthalene substitution [10] suggests that the pocket has limited capacity to expand and can optimally accommodate groups no larger than phenyl. Importantly, hydrophobic and aromatic interactions established at the P1 site have a higher impact on K_s_ than IC_50_. This was further demonstrated with the series V analogues, whose backbone elongation facilitated the insertion of R_1_ (compare the R_1_ insertion angle in Figure 9).

In contrast, the R_2_-mediated interactions at the P2 site largely affect the inhibitory potency, with naphthalene being the most optimal substituent. Notably, none of the designed analogues, including **3h** and **7d**, displace the I369-A370-M371 fragment as much as ritonavir: the A370 Cα atom shifts by 0.5, 0.9 and 1.8 Å, respectively (Figure 9). Thus, the R_2_-A370 clash is unfavourable and tends to be avoided or minimized. The active site expansion also proceeds through displacement of the F–F’ connecting loop, part of which (residues 210–214) is invisible in most of the CYP3A4 structures and does not seem to contribute much to the binding energy. Nonetheless, L210, L211 and F213 could establish hydrophobic interactions with the R_1_–phenyl and Boc-group (Figure S2B,D), which will be taken into consideration during optimization of the end-moiety.

In addition to the ability to strongly ligate to the heme via the N-pyridine heteroatom, all potent inhibitors establish an H-bond with the active site S119. The H-bonding propensity is diminished or completely lost in the S/R stereoisomers which, overall, have the lowest inhibitory potential. Stereochemistry becomes even more important when the side-groups are substituted with bulky indole and naphthalene, whose ability to adjust/rearrange in the active site is likely to be limited. This, in turn, could explain why the backbone flexibility and proper spacing between the functional groups are important and greatly improve the binding and inhibitory strength. Considering recent findings on P450 aromatase inhibitors [43], it is plausible to suggest that strong heme ligation, favourable stereochemistry (when applicable), H-bonding to the active site residue(s), and the deformation of the active site (induced fit) might be common features leading to the potent inhibition of CYP enzymes.

In conclusion, the spectral, functional and structural characterization of eleven series V analogues showed that six- and seven-atom separation between the pyridine and R_1_ is most optimal, as further linker extension lowers the binding and inhibitory strength by several-fold, likely due to spatial limitations in the active site and/or conformational constraints. Compared to the shorter series IV analogues, an increase in the R_2_ size/hydrophobicity has a less pronounced impact, partly due to the higher flexibility/mobility of the elongated compounds. Even so, the new lead compound, **3h**, was among the best CYP3A4 inhibitors designed to date with the highest binding affinity ever detected. Moreover, **3h** and other lead compounds most effectively prevented the H_2_O_2_-induced degradation of the heme, confirming once again that strong heme ligation and the blockage of the active site are critical for potent inhibition. Finally, the SAR analysis of series IV and V analogues suggests that further improvement in the inhibitory power could be achieved by balancing the flexibility/adjustability and rigidity of the scaffold.

## 4. Materials and Methods

Chemistry General Methods—All reactions were performed with commercially available reagents (Aldrich, Thermo-Fisher, Alfa Aesar, Acros, Oakwood, Millipore) without further purification. Anhydrous solvents were acquired through a solvent purification system (Inert PureSolv and JC Meyer systems) or purified according to standard procedures. ^1^H NMR spectra were recorded on Bruker DRX 400 MHz, Bruker DRX 500 MHz, or Bruker Avance 600 MHz spectrometer and processed using TopSpin 3.5 software. Low and high resolution mass spectrometry (LRMS and HRMS) data were obtained via ESI LC-TOF on a Waters (Micromass) LCT Premier spectrometer (Waters), with PEG as the calibrant for HRMS. Optical rotation was recorded on a Rudolph Autopol III Automatic Polarimeter at room temperature in methanol. Thin layer chromatography (TLC) was performed using EMD Millipore silica gel 60 F_254_ aluminium plates. Separation by column chromatography was conducted using Fisher silica gel 60 (230–400 mesh). All investigated compounds were >95% pure as determined by NMR. High resolution mass spectrometry data and NMR spectra are included in the Supplementary Material.

**Scheme 1:**
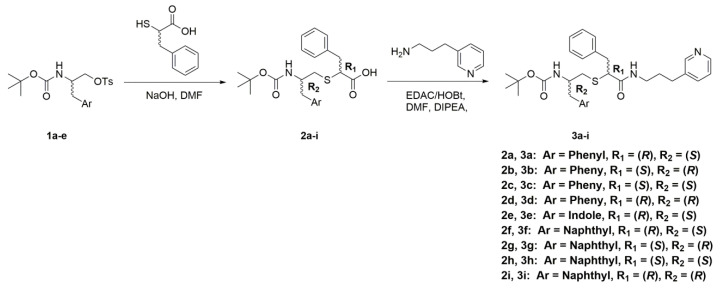
Synthesis of compounds **3a**–**i**.

**Scheme 2:**
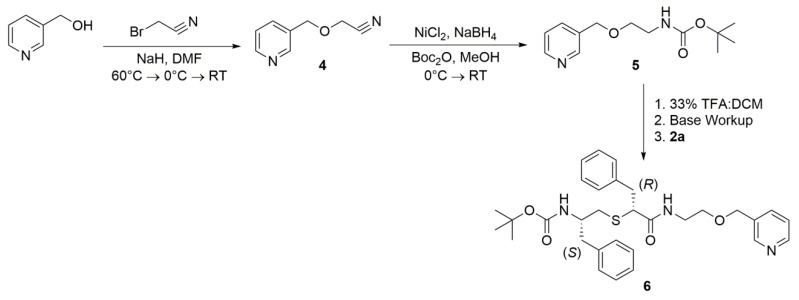
Synthesis of compound **6**. RT is room temperature.

**Scheme 3:**
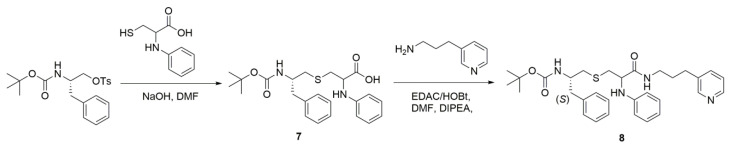
Synthesis of compound **8**.

Synthesis of Analogues—Compounds **1a**–**e** were prepared as described previously [10,22], with either boc-protection and the tosylation of commercially available amino alcohol or amino acid reduction and protection. Sequentially, the Ar groups are: **1a** phenyl (*S*), **1b** phenyl (*R*), **1c** indole (*S*), **1d** naphthyl (*S*), and **1e** naphthyl (*R*). Compounds **2a**–**i** were also prepared as described previously [10,22], with either *D* or *L-*α-thio-phenylalanine [44]. Synthesis of other analogues is outlined in Scheme 1, Scheme 2 and Scheme 3.

General Procedure for the Synthesis of Compounds **3a**–**i**— Steps for the synthesis of compounds **3a**-**i** are outlined in Scheme 1. Crude **2a** (0.34 g, 0.82 mmol) was dissolved in dimethylformamide (DMF) (3 mL). To this solution, 3-(3-pyridyl)propylamine (0.17 g, 1.23 mmol, 1.5 eq) in 3 mL DMF was added, followed by 1-ethyl-3-(3-dimethylaminopropyl)carbodiimide (EDAC) (0.24 g, 1.23 mmol, 1.5 eq), hydroxybenzotriazole (HOBt) (0.19 g, 1.23 mmol, 1.5 eq) and N,N-diisopropylethylamine (DIPEA) (0.32 g, 2.46 mmol, 3 eq). The reaction was stirred at room temperature overnight. Upon completion, the solvent was evaporated and the reaction mixture was diluted with ethyl acetate. The organic layer was then washed with saturated NaHCO_3_, water, and brine. The combined organic layers were dried over MgSO_4_ and concentrated in vacuo to give the crude product, which was purified via column chromatography (95:5 EtOAc:MeOH). The pure product **3a** was obtained as a clear yellow oil (0.07 g, 16%). TLC: EtOAc/MeOH 90:10 (Rf. 0.57). ^1^H NMR (400 MHz, CDCl_3_) δ 8.43 (d, *J* = 4.8 Hz, 1H), 8.37 (s, 1H), 7.43 (d, *J* = 7.7 Hz, 1H), 7.36–7.11 (m, 11H), 6.52 (t, *J* = 6.0 Hz, 1H (NH)), 4.63 (d, *J* = 8.3 Hz, 1H), 3.96 (m, 1H), 3.25 (quint, *J* = 6.7 Hz, 2H), 2.90 (m, 2H), 2.79 (d, *J* = 6.9 Hz, 2H), 2.68 (dd, *J* = 5.2, 13.6 Hz, 1H), 2.58 (dd, *J* = 6.1, 13.5 Hz, 1H), 2.48 (t, *J* = 7.9 Hz, 2H), 1.69 (quint, *J* = 7.3 Hz, 2H), 1.40 (s, 9H). HRMS *m/z* calculated for C_31_H_39_N_3_O_3_SNa [M + Na]^+^: 556.2610. Found: 556.2606. The pure product **3b** was obtained as a yellow gum (0.015 g, 15%). TLC: EtOAc/MeOH 90:10 (Rf. 0.57). ^1^H NMR (400 MHz, CDCl_3_) δ 8.45 (d, *J* = 4.9 Hz, 1H), 8.39 (s, 1H), 7.44 (d, *J* = 8.1 Hz, 1H), 7.31–7.16 (m, 9H), 7.13 (d, *J* = 6.8 Hz, 2H), 6.45 (t, *J* = 7.0 Hz, 1H (NH)), 4.57 (bs, 1H), 3.96 (m, 1H), 3.26 (quint, *J* = 7.0 Hz, 2H), 3.18 (m, 1H), 2.96 (dd, *J* = 7.0, 13.4 Hz, 1H), 2.79 (d, *J* = 7.0 Hz, 2H), 2.67 (dd, *J* = 5.7 13.5 Hz, 1H), 2.58 (dd, *J* = 6.0, 13.4 Hz, 1H), 2.49 (t, *J* = 7.7 Hz, 2H), 1.70 (quint, *J* = 7.4 Hz, 2H), 1.41 (s, 9H). HRMS *m/z* calculated for C_31_H_39_N_3_O_3_SNa [M + Na]^+^: 556.2610. Found: 556.2587. The pure product **3c** was obtained as a yellow gum (0.018 g, 18%). TLC: EtOAc/MeOH 90:10 (Rf. 0.57). ^1^H NMR (400 MHz, CDCl_3_) δ 8.44 (d, *J* = 4.8 Hz, 1H), 8.39 (s, 1H), 7.45 (d, *J* = 7.6 Hz, 1H), 7.32–7.17 (m, 9H), 7.08 (d, *J* = 6.7 Hz, 2H), 6.90 (bs, 1H (NH)), 4.64 (d, *J* = 6.9 Hz, 1H), 3.85 (q, *J* = 6.7 Hz, 1H), 3.54 (m, 1H), 3.30 (dd, *J* = 7.4, 13.7 Hz, 2H), 3.19 (quint, *J* = 6.7 Hz, 1H), 2.91 (m, 2H), 2.68 (m, 2H), 2.50 (m, 2H), 1.73 (quint, *J* = 7.5 Hz, 2H), 1.37 (s, 9H). HRMS *m/z* calculated for C_31_H_39_N_3_O_3_SNa [M + Na]^+^: 556.2610. Found: 556.2585. The pure product **3d** was obtained as a yellow gum (0.027 g, 21%). TLC: EtOAc/MeOH 90:10 (Rf. 0.57). ^1^H NMR (400 MHz, CDCl_3_) δ ^1^H NMR (400 MHz, CDCl_3_) δ 8.44 (d, *J* = 4.8 Hz, 1H), 8.37 (s, 1H), 7.45 (d, *J* = 7.8 Hz, 1H), 7.31–7.17 (m, 9H), 7.08 (d, *J* = 6.8 Hz, 2H), 6.89 (bs, 1H (NH)), 4.64 (d, *J* = 7.8 Hz, 1H), 3.85 (q, *J* = 7.0 Hz, 1H), 3.54 (m, 1H), 3.30 (dd, *J* = 7.3, 13.7 Hz, 2H), 3.19 (quint, *J* = 6.7 Hz, 1H), 2.91 (m, 2H), 2.69 (m, 2H), 2.49 (m, 2H), 1.73 (quint, *J* = 7.4 Hz, 2H), 1.39 (s, 9H). HRMS *m/z* calculated for C_31_H_39_N_3_O_3_SNa [M + Na]^+^: 556.2610. Found: 556.2598. Compound **3e** was obtained as a white fluffy solid (0.022 g, 13%). TLC: EtOAc/MeOH 90:10 (Rf. 0.49). ^1^H NMR (400 MHz, CDCl_3_) δ 8.44 (d, *J* = 4.8 Hz, 1H), 8.33 (s, 1H). In addition, 8.30 (s, 1H), 7.61 (d, *J* = 7.6 Hz, 1H), 7.41 (d, *J* = 7.8 Hz, 1H), 7.38–7.13 (m, 7H), 7.10 (t, *J* = 6.9 Hz, 1H), 6.98 (s, 1H), 6.33 (t, *J* = 5.2 Hz, 1H (NH)), 4.69 (d, *J* = 7.7 Hz, 1H), 4.09 (q, *J* = 6.6 Hz, 1H), 3.25 (dd, *J* = 7.4, 13.7 Hz, 1H), 3.12 (quint, *J* = 6.8 Hz, 1H), 2.97 (m, 4H), 2.71 (dd, *J* = 5.5, 13.9 Hz, 1H), 2.59 (dd, *J* = 6.2, 13.4 Hz, 1H), 2.40 (t, *J* = 7.7 Hz, 2H), 1.56 (quint, *J* = 7.4 Hz, 2H), 1.43 (s, 9H). HRMS *m/z* calculated for C_33_H_41_N_4_O_3_S [M + H]^+^: 573.2899. Found: 573.2900. The pure product **3f** was obtained as an off white fluffy solid (0.031 g, 20%). TLC: EtOAc/MeOH 90:10 (Rf. 0.51). ^1^H NMR (400 MHz, CDCl_3_) δ 8.43 (d, *J* = 4.7 Hz, 1H), 8.13 (d, *J* = 8.4 Hz, 1H), 7.82 (d, *J* = 8.0 Hz, 1H), 7.72 (d, *J* = 8.1 Hz, 1H), 7.55 (t, *J* = 7.8 Hz, 1H), 7.47 (t, *J* = 8.0 Hz, 1H), 7.36 (m, 3H), 7.31–7.16 (m, 7H), 6.34 (t, *J* = 5.3 Hz, 1H (NH)), 4.73 (d, *J* = 7.9 Hz, 1H), 4.16 (q, *J* = 6.3 Hz, 1H), 3.25 (m, 4H), 2.96 (dd, *J* = 6.8, 13.7 Hz, 2H), 2.68 (t, *J* = 4.6 Hz, 2H), 2.36 (t, *J* = 7.8 Hz, 2H), 1.53 (t, *J* = 7.4 Hz, 2H), 1.39 (s, 9H). HRMS *m/z* calculated for C_35_H_42_N_3_O_3_S [M + H]^+^: 584.2947. Found: 584.2921. The pure product **3g** was obtained as a yellow solid (0.024 g, 16%). TLC: EtOAc/MeOH 90:10 (Rf. 0.5). ^1^H NMR (400 MHz, CDCl_3_) δ 8.44 (d, *J* = 4.7 Hz, 1H), 8.13 (d, *J* = 8.3 Hz, 1H), 7.82 (d, *J* = 8.2 Hz, 1H), 7.72 (d, *J* = 8.0 Hz, 1H), 7.55 (t, *J* = 7.6 Hz, 1H), 7.47 (t, *J* = 8.0 Hz, 1H), 7.36 (m, 3H), 7.31–7.16 (m, 7H), 6.32 (t, *J* = 6.8 Hz, 1H (NH)), 4.72 (d, *J* = 8.0 Hz, 1H), 4.16 (q, *J* = 6.5 Hz, 1H), 3.25 (m, 4H), 2.96 (dd, *J* = 6.8, 13.7 Hz, 2H), 2.68 (t, *J* = 4.8 Hz, 2H), 2.36 (t, *J* = 6.8 Hz, 2H), 1.54 (t, *J* = 6.7 Hz, 2H), 1.39 (s, 9H). HRMS *m/z* calculated for C_35_H_42_N_3_O_3_S [M + H]^+^: 584.2947. Found: 584.2930. The pure product **3h** was obtained as an off white fluffy solid (0.089 g, 26%). TLC: EtOAc/MeOH 90:10 (Rf. 0.51 (Avg)). ^1^H NMR (400 MHz, CDCl_3_) δ 8.43 (d, *J* = 4.8 Hz, 1H), 8.35 (s, 1H), 8.16 (d, *J* = 7.8 Hz, 1H), 7.88 (d, *J* = 7.6 Hz, 1H), 7.77 (d, *J* = 8.2 Hz, 1H), 7.53 (quint, *J* = 7.6 Hz, 2H), 7.40 (t, *J* = 7.5 Hz, 2H), 7.29–7.16 (m, 7H), 6.81 (t, *J* = 5.3 Hz, 1H (NH)), 4.77 (d, *J* = 7.1 Hz, 1H), 4.08 (q, *J* = 6.4 Hz, 1H), 3.53 (m, 1H), 3.30 (m, 2H), 3.11 (m, 2H), 2.93 (dd, *J* = 6.0, 13.6 Hz, 1H), 2.79 (m, 1H), 2.54 (dd, *J* = 6.6, 13.9 Hz, 1H), 2.43 (sext, *J* = 6.3 Hz, 2H), 1.67 (oct, *J* = 7.4 Hz, 2H), 1.34 (s, 9H). HRMS *m/z* calculated for C_35_H_42_N_3_O_3_S [M + H]^+^: 584.2947. Found: 584.2928. The pure product **3i** was obtained as a white fluffy solid (0.07 g, 26%). TLC: EtOAc/MeOH 90:10 (Rf. 0.49). ^1^H NMR (400 MHz, CDCl_3_) δ 8.43 (d, *J* = 4.7 Hz, 1H), 8.35 (s, 1H), 8.16 (d, *J* = 7.8 Hz, 1H), 7.86 (dd, *J* = 8.2, 16.5 Hz, 1H), 7.75 (dd, *J* = 8.0, 19.1 Hz, 1H), 7.53 (m, *J* = 7.7 Hz, 2H), 7.40 (t, *J* = 7.7 Hz, 2H), 7.30–7.15 (m, 7H), 6.82 (bs, 1H (NH)), 4.77 (d, *J* = 8.0 Hz, 1H), 4.08 (sext, *J* = 6.7 Hz, 1H), 3.53 (m, 1H), 3.31 (dd, *J* = 8.5, 13.5 Hz, 2H), 3.13 (dd, *J* = 8.0, 14.8 Hz, 2H), 2.93 (dd, *J* = 6.2, 13.5 Hz, 1H), 2.79 (m, 1H), 2.54 (dd, *J* = 6.9, 14.3 Hz, 1H), 2.43 (sext, *J* = 5.9 Hz, 2H), 1.67 (sext, *J* = 7.6 Hz, 2H), 1.34 (s, 9H). HRMS *m/z* calculated for C_35_H_41_N_3_O_3_SNa [M + Na]^+^: 606.2766. Found: 606.2797. 

Synthesis of Compound **4**—Pyridine-3-methanol (2.0 g, 18.3 mmol) was dissolved in anhydrous DMF (30 mL) and the solution was cooled to 0 °C on an ice bath. Sodium hydride (0.88g, 36.6 mmol, 2 eq) was added slowly over 10 min. Once H_2_ formation subsided, the solution was warmed to 60 °C and stirred for 45 min. The solution was then cooled to 0 °C on an ice bath and bromoacetonitrile (2.74 g, 22.8 mmol, 1.25 eq) was added dropwise. The reaction was allowed to slowly come to room temperature overnight. Upon completion, the solvent was evaporated, and the reaction mixture was diluted with ethyl acetate. The organic layer was then washed with saturated NaHCO_3_, water, and brine. The combined organic layers were dried over MgSO_4_, filtered through a silica plug, and concentrated in vacuo to give the crude product, which was purified via column chromatography (100% EtOAc). The pure product **4** was obtained as a brown oil (0.65g, 24%). LRMS m/z calculated for C_8_H_8_N_2_ONa [M + Na]^+^: 171.0. Found: 171.0

Synthesis of Compound **5**—Compound **4** (0.5 g, 3.4 mmol) was dissolved in methanol (20 mL) and the solution was cooled to 0 °C on an ice bath. Nickel chloride hexahydrate (0.89 g, 3.7 mmol, 1.1 eq,) and Di-*tert*-butyl dicarbonate (1.5 g, 6.8 mmol, 2 eq) were then added to the solution, followed by slow addition of sodium borohydride (1.3 g, 34 mmol, 10 eq) over 10 min. Once H_2_ formation subsided, the solution was removed from the ice bath and allowed to stir at room temperature overnight. Upon completion, the solvent was evaporated, and the reaction mixture was diluted with ethyl acetate. The organic layer was then washed with saturated NaHCO_3_ and filtered through celite. The organic layers were dried over MgSO_4_ and concentrated in vacuo to give the crude product, which was purified via column chromatography (100% EtOAc). The pure product **5** was obtained as a brown oil (0.1 g, 12%). LRMS m/z calculated for C_13_H_20_N_2_O_3_Na [M + Na]^+^: 275.1. Found: 275.1.

Synthesis of Compound **6**—First, compound **5** (0.1 g, 0.40 mmol) was added to dichloromethane (DCM) (3 mL). Trifluoroacetic acid (1.5 mL) was added dropwise and the reaction was allowed to stir at room temperature for 2 h. Upon completion, the solvent was evaporated, and the reaction mixture was neutralized with NaHCO_3_. The pH was then adjusted to 14 with NaOH, and the solution was extracted with DCM. The combined organic layers were concentrated in vacuo to give the free amine product (2-(pyridine-3-ylmethoxy)ethan-1-amine) as a light-yellow oil (0.06g, 98%). HRMS m/z calculated for C_8_H_13_N_2_O [M + H]^+^: 153.1028. Found: 153.1043. Then, crude **2a** (0.15 g, 0.36 mmol) was dissolved in DMF (3 mL). To this solution, EDAC (0.1 g, 0.54 mmol, 1.5 eq) and HOBt (0.083 g, 0.54 mmol, 1.5 eq) were added, followed by the addition of 2-(pyridine-3-ylmethoxy)ethan-1-amine (0.06 g, 0.39 mmol, 1.1 eq) in DMF (2 mL) and DIPEA (0.14 g, 1.1 mmol, 3 eq). The reaction was stirred at room temperature overnight. Upon completion, the solvent was evaporated, and the reaction mixture was diluted with ethyl acetate. The organic layer was then washed with saturated NaHCO3, water, and brine. The combined organic layers were dried over MgSO_4_ and concentrated in vacuo to give the crude product, which was purified via column chromatography (100% EtOAc). The pure product **6** was obtained as a light orange oil (0.013 g, 6.5%). TLC: EtOAc/MeOH 90:10 (Rf. 0.54). ^1^H NMR (400 MHz, CDCl_3_) δ 8.54 (m, 2H), 7.64 (d, *J* = 6.2 Hz, 1H), 7.34–7.07 (m, 11H), 6.75 (m, 1H (NH)), 4.43 (s, 1H), 3.64 (m, 2H), 3.47 (m, 2H), 3.36 (s, 1H), 3.25 (dd, *J* = 6.9, 13.4 Hz, 1H), 2.95 (m, 2H), 2.73 (m, 2H), 2.65 (d, *J* = 6.8, 1H), 2.54 (dd, *J* = 6.5, 13.9 Hz, 1H), 2.35 (dd, *J* = 4.5, 19.4 Hz, 1H), 1.41 (s, 9H). HRMS *m/z* calculated for C_31_H_40_N_3_O_4_S [M + H]^+^: 550.2739. Found: 550.2738.

Compound **7** was prepared as described previously [21], with n-phenylcysteine and Boc-protected, tosylated L-phenylalanine (Boc–L–Phe–OTs).

Synthesis of Compound **8**—Crude **7** (0.34 g, 0.8 mmol) was dissolved in DMF (9 mL). To this solution, EDAC (0.23 g, 1.2 mmol, 1.5 eq) and HOBt (0.18 g, 1.2 mmol, 1.5 eq) were added, followed by the addition of 3-(3-pyridyl)propylamine (0.16 g, 1.2 mmol, 1.5 eq) and DIPEA (0.31 g, 2.4 mmol, 3 eq). The reaction was stirred at room temperature overnight. Upon completion, the solvent was evaporated, and the reaction mixture was diluted with ethyl acetate. The organic layer was then washed with saturated NaHCO_3_, water, and brine. The combined organic layers were dried over MgSO_4_ and concentrated in vacuo to give the crude product, which was purified via column chromatography (95:5 EtOAc:MeOH). The pure product **8** was acquired as a light-yellow gum (0.032 g, 23%). TLC: EtOAc/MeOH 90:10 (Rf. 0.55). ^1^H NMR (400 MHz, CDCl_3_) δ 8.43 (d, J = 4.7 Hz, 1H), 8.36 (s, 1H), 7.41 (m, 2H), 7.30–7.12 (m, 6H), 7.00 (t, J = 6.1 Hz, 1H), 6.85 (dt, J = 3.4, 11.0 Hz, 1H), 6.67 (t, J = 8.5 Hz, 2H), 4.70 (t, J = 10.6 Hz, 1H), 4.61 (bs, 1H (NH)), 4.00 (bs, 1H), 3.86 (bd, J = 28.8 Hz, 1H (NH)), 3.30 (m, 2H), 3.15 (m, 1H), 3.00 (m, 1H), 2.85 (m, 2H), 2.64 (m, 2H), 2.56 (t, J = 7.7 Hz, 2H), 1.87 (bs, 1H (NH)), 1.78 (m, 2H), 1.42 (d, J = 3.0 Hz, 9H). HRMS m/z calculated for C_31_H_40_N_4_O_3_SNa [M + Na]^+^: 571.2719. Found: 571.2698.

Protein Expression and Purification—Codon-optimized full-length and Δ3–22 human CYP3A4 were produced as reported previously [45] and used for assays and crystallization, respectively. The K421A and K424A mutations were introduced to the Δ3–22 CYP3A4 expression plasmid using a QuikChange mutagenesis kit (Stratagene) and confirmed by sequencing. The mutant was expressed and purified similar to the wild-ype (WT) protein.

Spectral Binding Titrations—Equilibrium ligand binding to CYP3A4 was monitored in a Cary 300 spectrophotometer at ambient temperature in 0.1 M phosphate buffer, pH 7.4, supplemented with 20% glycerol and 1 mM dithiothreitol. Inhibitors were dissolved in dimethyl sulfoxide (DMSO) and added to a 2 μM protein solution in small aliquots, with the final solvent concentration < 2%. Spectral dissociation constants (K_s_) were determined from quadratic fits to titration plots.

Thermal Denaturation—Thermal denaturation curves were recorded in 0.1 M phosphate buffer, pH 7.4, in a Cary 300 spectrophotometer. Protein (1 μM) was mixed with a ligand (20 μM) and incubated for 15 min at 23 °C. Melting curves were recorded at 260 nm using a 0.2 °C measurement step, 0.9 °C/min ramp rate, and a 50–75 °C temperature range. A denaturation midpoint (melting temperature; T_m_) was determined from non-linear fittings to the melting curve as described earlier [22].

Inhibitory Potency Assays—Inhibitory potency for the 7-benzyloxy-4-(trifluoromethyl)coumarin (BFC) *O*-debenzylation activity of CYP3A4 was evaluated fluorometrically in a soluble reconstituted system. The full-length CYP3A4 and rat cytochrome P450 reductase (40 and 60 μM, respectively) were preincubated at room temperature for 1 h before 20-fold dilution with the reaction buffer consisting of 0.1 M potassium phosphate, pH 7.4, catalase and superoxide dismutase (2 units/mL each), and 0.0025% CHAPS (3-[(3-cholamidopropyl)dimethyl-ammonio]-1-propanesulfonate). Prior to measurements, 85 μL of the reaction buffer was mixed with 10 μL of the NADPH-regenerating system (10 mM glucose, 0.2 mM NADP^+^, and 2 units/mL glucose-6-phosphate dehydrogenase), 5 μL of the protein mixture (0.1 μM final CYP3A4 concentration), and 2 μL of the inhibitor solution or DMSO. The mixture was incubated for 2 min, after which 20 μL BFC and 70 μL NADPH (final concentration) were added to initiate the reaction. Accumulation of the fluorescent product, 7-hydroxy-4-(trifluoromethyl)coumarin, was monitored for 2 min at room temperature in a Hitachi F400 fluorimeter (λ_ex_ = 404 nm; λ_em_ = 500 nm). Within this time interval, fluorescence changes were linear. The average of three measurements was used to calculate the remaining activity, with the DMSO-containing sample used as a control (100% activity). The IC_50_ values were derived from the (% activity) vs. (inhibitor concentration) plots as described previously [22]. Our prior work showed that preincubation of ritonavir or the analogues with NADPH in a lipid-free reconstituted system leads to a small increase rather than decrease in IC_50_, likely to due to partial metabolism [22]. Therefore, in this study, inhibitory assays with NADPH preincubation were not conducted.

Kinetics of Ligand Binding and Heme Reduction—Kinetics of ligand binding to CYP3A4 and its reduction with sodium dithionite were measured at 426 and 443 nm, respectively, in a SX.18MV stopped flow apparatus (Applied Photophysics, UK), as described earlier [22].

H_2_O_2_-Dependent Heme Depletion Assay—Heme bleaching in ligand-free and inhibitor-bound CYP3A4 (1.6 μM) was monitored at ambient temperature in 0.1 M phosphate buffer, pH 7.4. After 10 min preincubation of CYP3A4 with 16 μM inhibitors, 10 mM H_2_O_2_ (final concentration) was added, and heme decay was monitored at 420 nm for 120 min. The percentage of heme destroyed at the end of the reaction was calculated relative to that in the ligand-free CYP3A4 (100% decay).

Crystallization of the Inhibitory Complexes—Compounds **3a**–**d**, **8**, and **3e**–**h** were co-crystallized with WTΔ3-22 CYP3A4, and **3d** and **3i** with the K421A/K424A mutant. Crystals of **3b**-, **3c**-, **3f**-, **3g**- and **8**-bound complexes were grown using a microbatch method under paraffin oil. Other compounds were co-crystallized with CYP3A4 using a sitting drop vapor diffusion method. Prior to crystallization setup, CYP3A4 (60–70 mg/mL in 20–100 mM phosphate, pH 7.4) was incubated with a 2-fold ligand excess and centrifuged to remove the precipitate. The supernatant (0.4–0.6 μL) was mixed with 0.4–0.6 μL of crystallization solution containing: 8%–10% polyethylene glycol (PEG) 3350 and 70–80 mM sodium malonate, pH 7.0, for **3a**, **3g** and **8**; 10%–12% PEG 3350 and 60–100 mM succinate, pH 7.0, for **3b** and **3f**; 10% PEG 3350 and 80 mM taximate, pH 6.0 (Hampton Research) for **3c**; 6% PEG 3350 and 50 mM sodium malonate, pH 7.0, for **3e**; or 6%–8% PEG 4000, 50 mM HEPES, pH 8.0, and 0.1–0.2 M lithium sulfate for **3d**, **3h** and **3i**. Crystals were grown at room temperature for 2–3 days and cryoprotected with Paratone-N before freezing in liquid nitrogen.

Determination of the X-Ray Structures—X-ray diffraction data were collected at the Stanford Synchrotron Radiation Lightsource beamlines 9–2 and 12–2, and the Advanced Light Source beamlines 5.0.2 and 8.2.2. Crystal structures were solved by molecular replacement with PHASER [46]. In addition, 5VCC was used as a search model for the **3b**-, **3c**-, **3f**-, **3g**- and **8**-bound complexes, crystallized in the I222 space group. For the **3a**-, **3d**-, **3h**- and **3i**-bound CYP3A4, crystallized in the C2 and I2 space groups, the search model was the CYP3A4-**3e** dimer, whose structure was determined first using 6UNJ for molecular replacement. Ligands were built with eLBOW [47] and manually fit into the density with COOT [48]. The initial models were rebuilt and refined with COOT and PHENIX [47]. For racemic **8**, the R_1_ side-group configuration was automatically assigned to *R* during the first refinement cycle. The following refinement was conducted with all stereoisomer combinations to confirm that the assigned chirality was most optimal. Polder omit electron density maps and local correlation coefficients (Table S3) were calculated with PHENIX. Data collection and refinement statistics are summarized in Tables S1 and S2. The atomic coordinates and structure factors for the **3a**-, **3b**-, **3c**-, **3d**-, **8**-, **3e**-, **3f**-, **3g**-, **3h**-, and **3i**-bound CYP3A4 were deposited in the Protein Data Bank with the ID codes 7KVH, 7KVI, 7KVJ, 7KVK, 7KVM, 7KVN, 7KVO, 7KVP, 7KVQ and 7KVS, respectively.

## Figures and Tables

**Table 1:** Properties of series V inhibitors.

Compound	λ_max_ (nm)	A_421/417_^a^	ΔA_max_^b^	K_s_^c^	IC_50_^d^	IC_50_/K_s_	ΔT_m_^e^	*k*_fast_^f^	*k* ^ET g^
	Ferric/ferrous			μM	μM		°C	s^−1^	s^−1^
*Pyridyl-propyl linker*									
*4-atom R_1_(phenyl)-R_2_(phenyl) spacer*									
**3a** (*R*, *S*)	422/444	1.13	122%	0.015 ± 0.003	0.16 ± 0.02	10.7	8.1	12.5 (36%)	0.011 (98%)
**3b** (*S*, *R*)	421/443	1.08	111%	0.029 ± 0.004	0.30 ± 0.02	10.3	5.3	9.5 (35%)	0.014 (87%)
**3c** (*S*, *S*)	422/444	1.09	111%	0.026 ± 0.002	0.31 ± 0.04	11.9	5.8	9.5 (33%)	0.012 (77%)
0.012 (77%)	422/444	1.10	114%	0.013 ± 0.005	0.21 ± 0.02	20.0	5.6	12.0 (38%)	0.013 (95%)
*5-atom R_1_(phenyl)-R_2_(phenyl) spacer*									
**8** (*rac*, *S*)	422/444	1.10	114%	0.028 ± 0.004	0.33 ± 0.03	7.5	5.0	16.2 (30%)	0.018 (58%)
*Pyridyl-mehtoxyethyl (butyl-like) linker*									
*4-atom R_1_(phenyl)-R_2_(phenyl) spacer*									
**6** (*R*, *S*)	422/443	1.07	113%	0.213 ± 0.009	0.73 ± 0.06	3.4	3.0	9.5 (29%)	0.017 (65%)
*Pyridyl-propyl linker*									
*4-atom R_1_(phenyl)-R_2_(indol) spacer*									
**3e** (*R*, *S*)	422/444	1.11	125%	0.019 ± 0.002	0.15 ± 0.03	7.9	7.8	20.1 (24%)	0.020 (85%)
*Pyridyl-propyl linker*									
*4-atom R_1_(phenyl)-R_2_(naphthalene) spacer*									
**3f** (*R*, *S*)	422/444	1.12	125%	0.015 ± 0.001	0.16 ± 0.03	10.7	7.9	19.3 (28%)	0.011 (94%)
**3g** (*S*, *R*)	422/444	1.09	111%	0.024 ± 0.006	0.30 ± 0.06	12.5	4.9	10.8 (32%)	0.011 (75%)
**3h** (*S*, *S*)	422/444	1.09	112%	0.007 ± 0.001	0.09 ± 0.01	12.9	5.9	16.1 (24%)	0.017 (72%)
**3i** (*R*, *R*)	422/444	1.10	114%	0.018 ± 0.003	0.15 ± 0.02	8.3	5.5	12.3 (24%)	0.021 (97%)

^a^ The ratio between the absorbance maxima of ferric ligand-bound and ligand-free CYP3A4. ^b^ Maximal absorbance change (difference between the peak and trough in the difference spectrum) relative to that induced by ritonavir. ^c^ Spectral dissociation constant for the CYP3A4–inhibitor complex determined from quadratic fits to titration plots (right insets in Figures 3, 5 and 6). ^d^ Inhibitory potency measured for the 7-benzyloxy-4-(trifluoromethyl)coumarin (BFC) debenzylase activity in a soluble reconstituted system with cytochrome P450 reductase. ^e^ Ligand-dependent change in the melting temperature of CYP3A4. ^f^ Rate constant for the fast phase of the ligand binding reaction. Values in the brackets are the percentage of the fast phase. ^g^ Rate constant for reduction of inhibitor-bound CYP3A4 with sodium dithionite. Values in the brackets are a portion of the protein reduced.

**Table 2:** Structural features of CYP3A4–inhibitor complexes.

Compound	Fe–N Bond		Pyridine ring	I-helix	H-Bond	Pyridine–R_2_ Ring		F304–R_1_ Ring		*Boc*-Group
	Distance (Å) Angle (°) ^a^		Rotation (°) ^b^	Shift (Å) ^c^	with S119 (Å) ^d^	Angle and Overlap		Angle and Overlap		Contacts
**3a** (*R*, *S*) mol A	2.11	3	30	2.04–2.18	2.82	42°	full	50°	partial	108, 211, 213
**3b** (*S*, *R*)	2.20	15	17	1.09–1.76	3.25	46°	half ^e^	65°	partial ^e^	disordered
**3c** (*S*, *S*)	2.18	7	12	1.23–1.76	3.08	35°	partial	36°	half	105, 106, 108, 374
**3d** (*R*, *R*) mol A	2.18	0	37	2.11–2.18	2.48	5°	full	50°	half	57, 108, 211, 213, 482
**8** (*R*, *S*)	2.11	3	32	1.77–1.97	3.15 ^f^	25°	half	20°	full	disordered
**3e** (*R*, *S*) mol A	2.08	2	37	0.81–1.34	2.30	33°	full	50°	half	108, 211, 213
**3f** (*R*, *S*) mol A	2.07	0	33	1.90–2.11	2.41	45°	half	40°	half	105–108
**3g** (*S*, *R*)	2.23	6	10	0.47–0.42	-	30°	half	85°	none	disordered
**3h** (*S*, *S*) mol A	2.07	3	35	1.98–2.21	2.35	45°	full	40°	half	105–108
**3i** (*R*, *R*) mol A	2.09	4	30	1.96–2.14	2.30	20°	full	30°	half	105–108, 374

^a^ Deviation from perpendicularity. ^b^ Angle between the planes passing through the pyridine ring and the NB–ND heme atoms. ^c^ Distance between the Cα-atoms of F304 and A305 in the inhibitor- and water-bound CYP3A4 (5VCC structure). ^d^ Hydrogen bond length between the inhibitor’s carbonyl oxygen and S119 hydroxyl group. ^e^ In **3b**, the R_1_ and R_2_ side groups are in reverse orientation and placed near the heme-ligating pyridine and F304, respectively. ^f^ H-bonding via the amide nitrogen atom.

## Data Availability

Structure factors and coordinate files for the described structures are publicly available in the Protein Data Base.

## Supplementary Materials

Supplementary Materials can be found at https://www.mdpi.com/1422-0067/22/2/852/s1 and include figures showing reorganization of the F–F’ fragment in C2 crystals induced by different packing with a view at the distal face of WT and K421A/K424A CYP3A4, a comparison of the 3a and 3h binding modes in I222 and C2 crystals, absolute spectra of ferric and ferrous ligand-free and 3d-, 3i- and ritonavir-bound CYP3A4 K421A/K2424A with the respective titrations plots, X-ray data collection and refinement statistics, correlations coefficients for polder omit maps, ^1^H NMR spectra, and high-resolution mass spectrometry data.

## Abbreviations

CYP3A4	Cytochrome P450 3A4
BFC	7-benzyloxy-4-(trifluoromethyl)coumarin
SAR	Structure–activity relationship
WT	Wild type
