# Supplementary material for: Rational Design of CYP3A4 Inhibitors: A One-Atom Linker Elongation in Ritonavir-Like Compounds Leads to a Marked Improvement in the Binding Strength
Source: Int J Mol Sci. 2021 Jan 16;22(2):852. doi: 10.3390/ijms22020852 (PMC7830545; doi:10.3390/ijms22020852)
Supplement: Supplementary file 1 [file ijms-22-00852-s001.pdf]

Rational design of CYP3A4 inhibitors: A one-atom linker elongation in ritonavir-like compounds leads to a marked improvement in the binding strength

Eric R. Samuels[†ƒ] and Irina F. Sevrioukova[‡]*

Departments of [†]Pharmaceutical Sciences and [‡]Molecular Biology and Biochemistry, University of California, Irvine, California 92697-3900

SUPPLEMENTAL MATERIALS

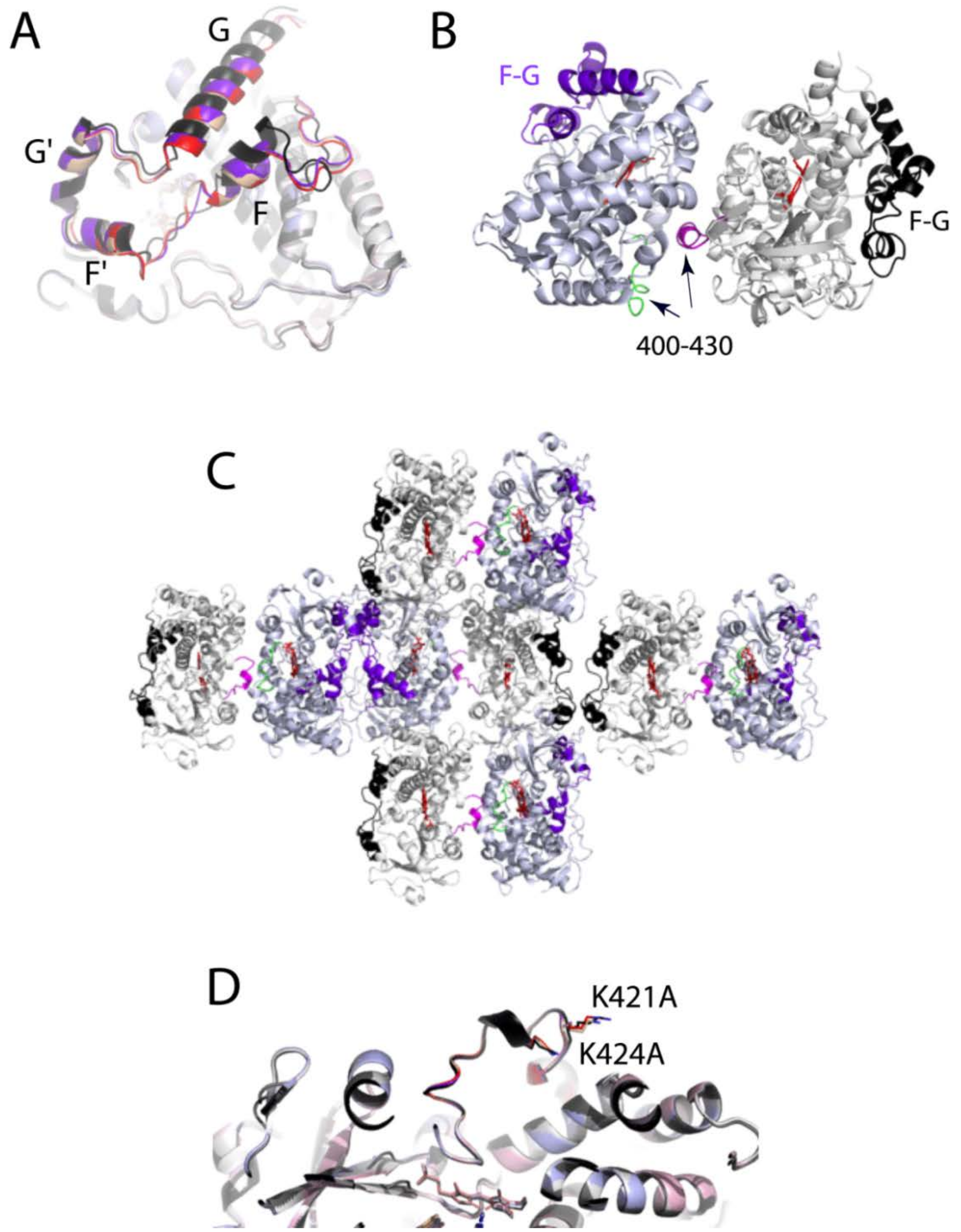

Figure S1. (**A**) Restructuring of the F-F’ helix/loop region triggered by distinct crystal packing in the C2 space group. The F-F’-G’-G fragments of ligand-free (5VCE; I222) and **3a**-, **3e**- and **3**i-bound CYP3A4 (C2) are shown in black, red, beige and purple, respectively. (**B, C**) Crystallographic dimer of CYP3A4 and its spatial arrangement in C2 crystals, respectively. The 400-430 fragment (in green and magenta) is at the monomer-monomer interface, whereas the F-G fragment (in black and purple) mediates inter-dimer contacts. (**D**) A view at the proximal face of CYP3A4. The water-bound protein (5VCC structure) is in gray; **3a**- and **3e**-bound complexes of WT CYP3A4 are in pink and light blue, respectively; and **3**i-bound K421A/K424A CYP3A4 is in black. Structural superposition shows that substitution of surface K421 and K424 with alanine does not distort the proximal loop region. Non-mutated lysine side chains are displayed.

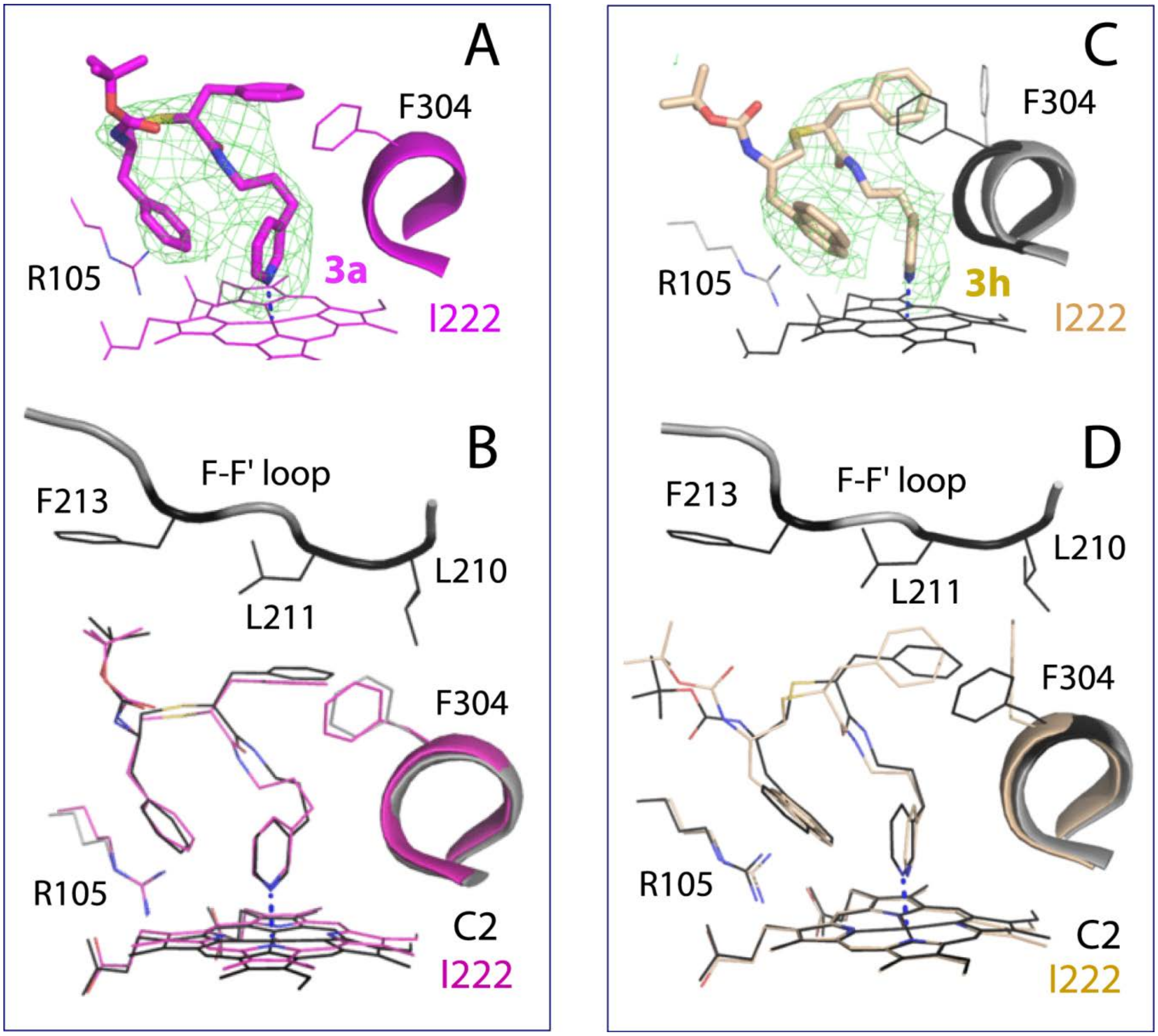

Figure S2. Binding of **3a** (**A**, **B**) and **3h** (**B**, **C**) to the active site of CYP3A4 crystallized in I222 and C2 space groups. For both **3a** and **3h**, electron density maps were not clearly defined in the I222 structures (green mesh in **A** and **C**, respectively). Therefore, **3a**- and **3h**-bound CYP3A4 was recrystallized in a more densely packed C2 space group. (**B**, **D**) Comparison of the ligand binding modes observed in the I222 and C2 structures shows that there are virtually no (for **3a**) or only minor distortions (for **3h**), primarily imposed by changes in the F-F' loop (residues 210-213).

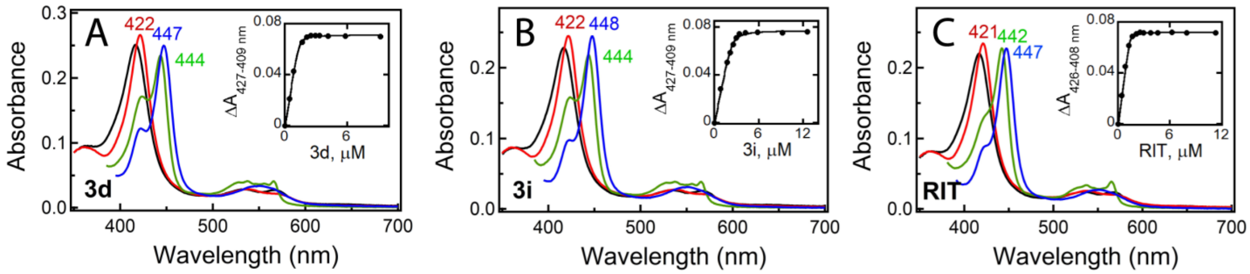

Figure S3. Spectral and ligand-binding properties of K421A/K424A CYP3A4. (**A**-**C**) Spectral changes induced in the mutant by **3d**, **3i** and ritonavir, respectively. Absorbance spectra of ferric ligand-free and inhibitor-bound CYP3A4 (2 µM) were recorded in 0.1 M phosphate buffer, pH 7.4, supplemented with 20% glycerol and 1 mM dithiothreitol, and displayed in black and red, respectively. Spectra of the ferrous form and its CO-adduct are in green and blue, respectively. The inhibitor concentration was 10 µM. Equilibrium titrations were conducted as described in the Materials and Methods section in the main text. Titration plots with quadratic fittings are shown in insets. The derived dissociation constants were 0,019 µM, 0.025 µM and 0.023 µM for **3d**, **3i** and ritonavir, respectively.

Table S1. Data collection and refinement statistics

Ligand	**3a**	**3b**	**3c**	**3d**	**8**
CYP3A4	WT	WT	WT	K421A/K424A	WT
PDB ID	7KNH	7KVI	7KVJ	7KVK	7KVM
Data statistics					
Space group	C2	I222	I222	C2	I222
Unit cell	*a, b, c* = 93 x 95 x 153 Å; α, β, γ = 90 x 90 x 124°	*a, b, c* = 77 x 103 x 126 Å; α, β, γ = 90°	*a, b, c* = 76 x 100 x 124 Å; α, β, γ = 90°	*a, b, c* = 76 x 101 x 125 Å; α, β, γ = 90°	*a, b, c* = 78 x 101 x 129 Å; α, β, γ = 90°
Resolution range (Å)	39.55-2.79 (2.79-2.94)[a]	79.64-2.55 (2.55-2.69)	78.17-2.65 (2.79-2.65)	77.80-2.55 (2.69-2.55)	79.64-2.70 (2.85-2.70)
Total reflections	167,933	81,756	51,716	159,292	56,723
Unique reflections	27,176	16,474	13,659	37,311	13,668
Redundancy	6.2 (6.0)	5.0 (4.8)	3.8 (4.0)	4.3 (4.4)	4.2 (4.3)
Completeness	98.3 (96.9)	99.2 (99.5)	97.1 (98.3)	99.8 (99.8)	95.9 (97.4)
Average $I/\sigma I$	5.8 (1.1)	8.3 (1.0)	7.4 (1.0)	8.4 (1.1)	8.5 (0.9)
R_{merge}	0.163 (2.561)	0.077 (1.163)	0.070 (2.029)	0.080 (1.443)	0.047 (2.926)
R_{pim}	0.107 (1.155)	0.038 (0.592)	0.042 (1.127)	0.043 (0.768)	0.038 (1.358)
CC ½	0.993 (0.489)	0.998 (0.532)	0.998 (0.320)	0.999 (0.398)	0.996 (0.313)
Refinement statistics					
R/R_{free}[b]	23.8/27.8	22.5/26.2	22.5/28.0	23.7/27.7	22.7/27.6
Number of atoms:					
Protein	3657/3555[c]	3654	3341	3716/3581	3720
Solvent	24	21	4	14	0
R.m.s. deviations					
Bond lengths, Å	0.002	0.002	0.002	0.002	0.003
Bond angles, °	0.490	0.531	0.490	0.467	0.661
Wilson B-factor, $Å^2$	83	79	96	73	112
Average *B*-factor, $Å^2$:					
Protein	91/110[c]	112	136	89/131	141
Ligand	85/104[c]	140	162	102/126	149
Ramachandran plot[d] (residues; %)					
Preferred	816 (93.0%)	427 (96.0%)	388 (95.0%)	848 (94.9%)	435 (95.0%)
Allowed	60 (6.8%)	17 (3.8%)	20 (5.0%)	44 (4.9%)	25 (5.0%)
Outliers	2 (0.2%)	1 (0.2%)	none	2 (0.2%)	none

[a]Values in brackets are for the highest resolution shell.
[b]R_{free} was calculated from a subset of 5% of the data that were excluded during refinement.
[c]Values for two molecules in the asymmetric unit.
[d]Analyzed with PROCHECK.

Table S2. Data collection and refinement statistics

Ligand	**3e**	**3f**	**3g**	**3h**	**3i**
CYP3A4	WT	WT	WT	WT	K421A/K424A
PDB ID	7KVN	7KVO	7KVP	7KVQ	7KVS
Data statistics					
Space group	C2	I2	I222	C2	C2
Unit cell	*a, b, c* = 93 x 96 x 153 Å; α, β, γ = 90 x 90 x 124°	*a, b, c* = 93 x 98 x 128 Å; α, β, γ = 90 x 90 x 93°	*a, b, c* = 78 x 102 x 128 Å; α, β, γ = 90°	*a, b, c* = 93 x 97 x 154 Å; α, β, γ = 90 x 90 x 124°	*a, b, c* = 93 x 98 x 155 Å; α, β, γ = 90 x 90 x 124°
Resolution range (Å)	76.93-2.70 (2.85-2.70)[a]	49.04-2.65 (2.79-2.65)	80.00-2.75 (2.907-2.75)	77.28-2.70 (2.85-2.70)	77.82-2.50 (2.64-2.50)
Total reflections	136,528	223,503	43,178	177,490	246,329
Unique reflections	28,152	33,059	13,250	30,116	38,953
Redundancy	4.8 (4.3)	6.8 (6.7)	3.3 (3.4)	5.9 (6.0)	6.3 (6.3)
Completeness	91.9 (84.0)	99.9 (99.9)	97.3 (98.6)	94.4 (97.2)	97.6 (97.4)
Average $I/\sigma I$	4.0 (1.0)	12.3 (1.1)	5.9 (0.7)	8.5 (1.1)	11.0 (1.0)
R_{merge}	0.233 (1.598)	0.070 (2.278)	0.072 (2.466)	0.093 (1.641)	0.075 (1.922)
R_{pim}	0.115 (0.841)	0.029 (0.944)	0.045 (1.566)	0.040 (0.723)	0.032 (0.830)
CC ½	0.970 (0.571)	0.999 (0.390)	0.998 (0.481)	0.999 (0.372)	0.999 (0.345)
Refinement statistics					
R/R_{free}[b]	24.8/29.0	24.1/29.3	22.7/27.3	25.0/28.0	23.5/27.5
Number of atoms:					
Protein	3653/3646[c]	3721/3631	3653	3669/3605	3689/3538
Solvent	88	0	2	0	47
R.m.s. deviations					
Bond lengths, Å	0.003	0.003	0.004	0.002	0.002
Bond angles, °	0.563	0.570	0.731	0.497	0.500
Wilson B-factor, $Å^2$	69	91	113	89	80
Average *B*-factor, $Å^2$:					
Protein	75/93[c]	107/160	96	100/167	99/151
Ligand	65/88[c]	164/177	163	128/177	132/182
Ramachandran plot[d] (residues; %)					
Preferred	826 (92.9%)	842 (94.1%)	435 (97.0%)	837 (93.9%)	836 (94.1%)
Allowed	62 (9.0%)	52 (5.8%)	14 (3.0%)	53 (6.0%)	51 (5.8%)
Outliers	1 (0.1%)	1 (0.1%)	none	1 (0.1%)	1 (0.1%)

[a]Values in brackets are for the highest resolution shell.
[b]R_{free} was calculated from a subset of 5% of the data that were excluded during refinement.
[c]Values for two molecules in the asymmetric unit.
[d]Analyzed with PROCHECK

Table S3. Local correlation coefficients (CC) and CC_{peak} between three polder maps ($F_{obs} = |F_{model}|$)

Map 1 (m1), calculated F_{obs} assuming that the omitted ligand atoms are present. Map 2 (m2), calculated F_{obs} assuming that the omitted ligand atoms are not present. Map 3 (m3), polder map using experimental data.

		m1-m2		m1-m3		m2-m3	
Ligand/PDB ID		CC	CC_{peak}	CC	CC_{peak}	CC	CC_{peak}
**3a**	7KVH	0.4949	0.4797	0.8790	0.8579	0.4998	0.4709
**3b**	7KVI	0.6654	0.6106	0.8934	0.8439	0.6464	0.6431
**3c**	7KNJ	0.6304	0.6167	0.8333	0.7082	0.4517	0.3954
**3d**	7KVK	0.5390	0.4609	0.9078	0.8797	0.5149	0.4463
**8**	7KVM	0.6183	0.5721	0.8442	0.7824	0.6879	0.6878
**3e**	7KVN	0.5025	0.5048	0.8715	0.8431	0.4884	0.4780
**3f**	7KVO	0.7812	0.7598	0.8015	0.7899	0.6965	0.6741
**3g**	7KVP	0.6169	0.6394	0.8884	0.8461	0.5352	0.5618
**3h**	7KVQ	0.6701	0.6616	0.8005	0.7598	0.6414	0.6466
**3i**	7KVS	0.6324	0.6383	0.8551	0.8222	0.7047	0.6985

Correlation coefficients for polder maps were calculated using phenix.*polder* in the PHENIX software package.[1]

For all ligands, CC(1,3) was > 0.8 and higher than CC(1,2) and CC(2,3), meaning that the polder maps displayed in Figures 3 and 6 of the main text show the omitted ligand atoms.[2]

[1]Adams, P.D.; Afonine, P.V.; Bunkoczi, G.; Chen, V.B.; Davis, I.W.; Echols, N.; Headd, J.J.; Hung, L.W.; Kapral, G.J.; Grosse-Kunstleve, R.W., et al. PHENIX: a comprehensive Python-based system for macromolecular structure solution. *Acta Crystallogr. Section D* **2010**, *66*, 213-321

[2]Liebschner D., Afonine P.V.; Moriarty N.W.; Poon B.K.; Sobolev O.V.; Terwilliger T.C.; Adams P.D. Polder maps: improving OMIT maps by excluding bulk solvent. *Acta Crystallogr. Section D* **2016**, *73*, 148-157.

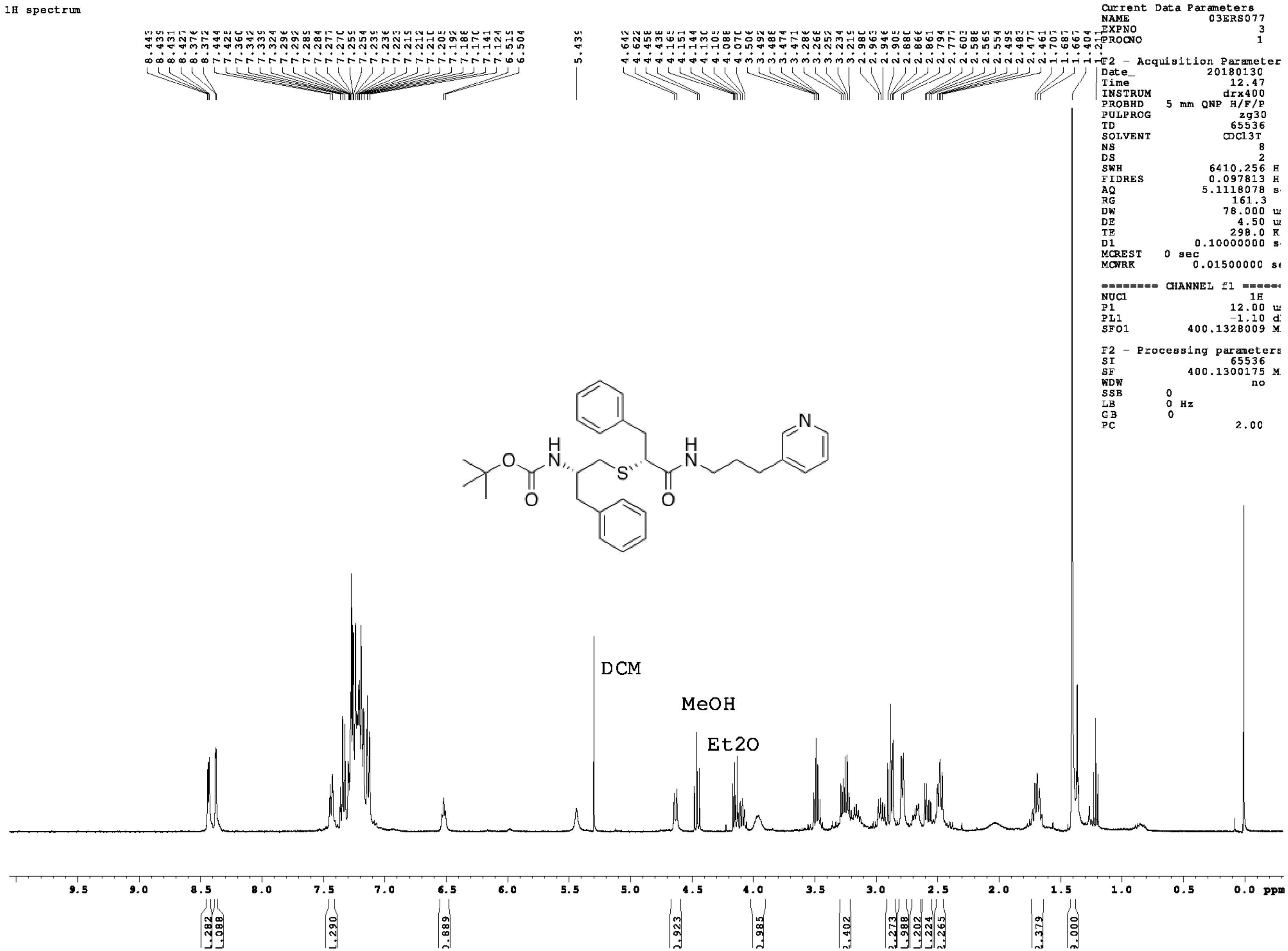
1H spectrum
DCM
MeOH
Et2O
Current Data Parameters
NAME 03ERS077
EXPNO 3
PROCNO 1
F2 - Acquisition Parameter
Date_ 20180130
Time 12.47
INSTRUM drx400
PROBHD 5 mm QNP H/F/P
PULPROG zg30
TD 65536
SOLVENT CDCl3T
NS 8
DS 2
SWH 6410.256 H
FIDRES 0.097813 H
AQ 5.1118078 s
RG 161.3
DW 78.000 u
DE 4.50 u
TE 298.0 K
D1 0.10000000 s
MCREST 0 sec
MCWRK 0.01500000 s
======== CHANNEL f1 =====
NUC1 1H
P1 12.00 u
PL1 -1.10 d
SFO1 400.1328009 M
F2 - Processing parameters
SI 65536
SF 400.1300175 M
WDW no
SSB 0
LB 0 Hz
GB 0
PC 2.00
ppm

1H spectrum

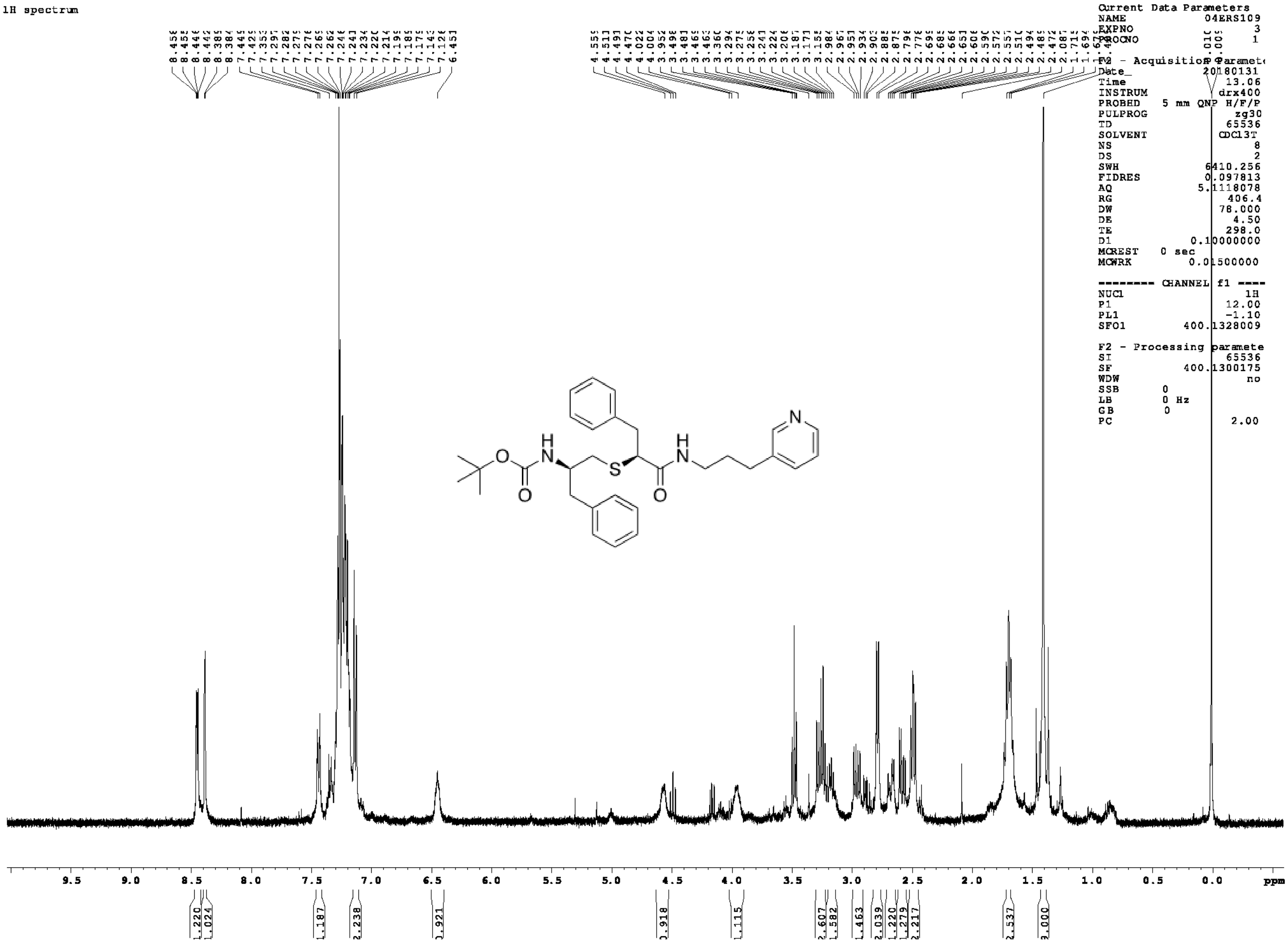
Current Data Parameters
NAME 04ERS109
EXPNO 3
PROCNO 1
F2 - Acquisition Parameters
Date_ 20180131
Time 13.06
INSTRUM drx400
PROBHD 5 mm QNP H/F/P
PULPROG zg30
TD 65536
SOLVENT CDCl3T
NS 8
DS 2
SWH 6410.256
FIDRES 0.097813
AQ 5.1118078
RG 406.4
DW 78.000
DE 4.50
TE 298.0
D1 0.10000000
MCREST 0 sec
MCWRK 0.01500000
======== CHANNEL f1 ========
NUC1 1H
P1 12.00
PL1 -1.10
SFO1 400.1328009
F2 - Processing parameters
SI 65536
SF 400.1300175
WDW no
SSB 0
LB 0 Hz
GB 0
PC 2.00
9.5
9.0
8.5
8.0
7.5
7.0
6.5
6.0
5.5
5.0
4.5
4.0
3.5
3.0
2.5
2.0
1.5
1.0
0.5
0.0
ppm

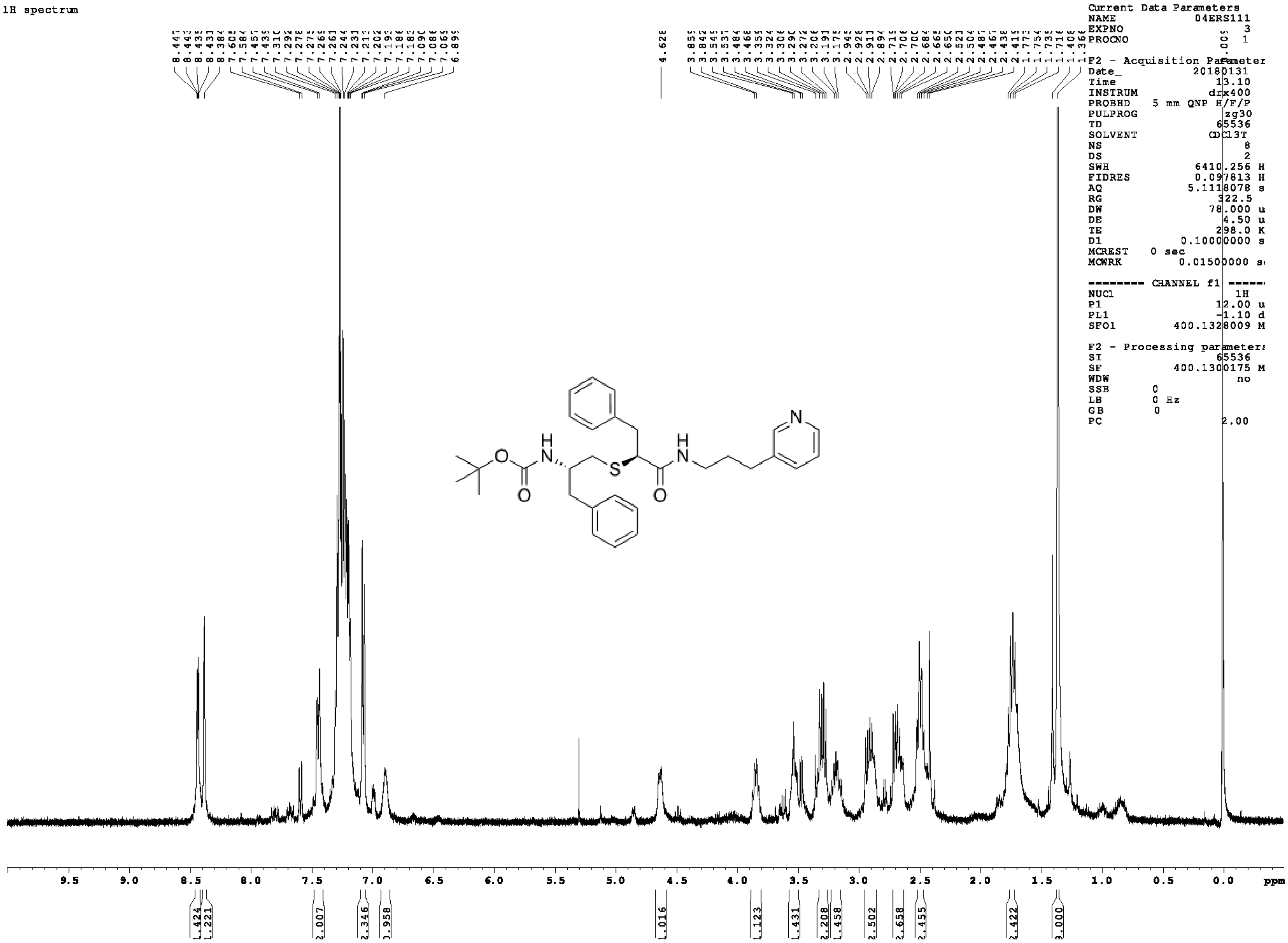
1H spectrum
Current Data Parameters
NAME 04ERS111
EXPNO 3
PROCNO 1
F2 - Acquisition Parameters
Date_ 20180131
Time 13.10
INSTRUM drx400
PROBHD 5 mm QNP H/F/P
PULPROG zg30
TD 65536
SOLVENT CDCl3T
NS 8
DS 2
SWH 6410.256 H
FIDRES 0.097813 H
AQ 5.1118078 s
RG 322.5
DW 78.000 u
DE 4.50 u
TE 298.0 K
D1 0.10000000 s
MCREST 0 sec
MCWRK 0.01500000 s
======== CHANNEL f1 ========
NUC1 1H
P1 12.00 u
PL1 -1.10 d
SFO1 400.1328009 M
F2 - Processing parameters
SI 65536
SF 400.1300175 M
WDW no
SSB 0
LB 0 Hz
GB 0
PC 2.00
9.5 9.0 8.5 8.0 7.5 7.0 6.5 6.0 5.5 5.0 4.5 4.0 3.5 3.0 2.5 2.0 1.5 1.0 0.5 0.0 ppm

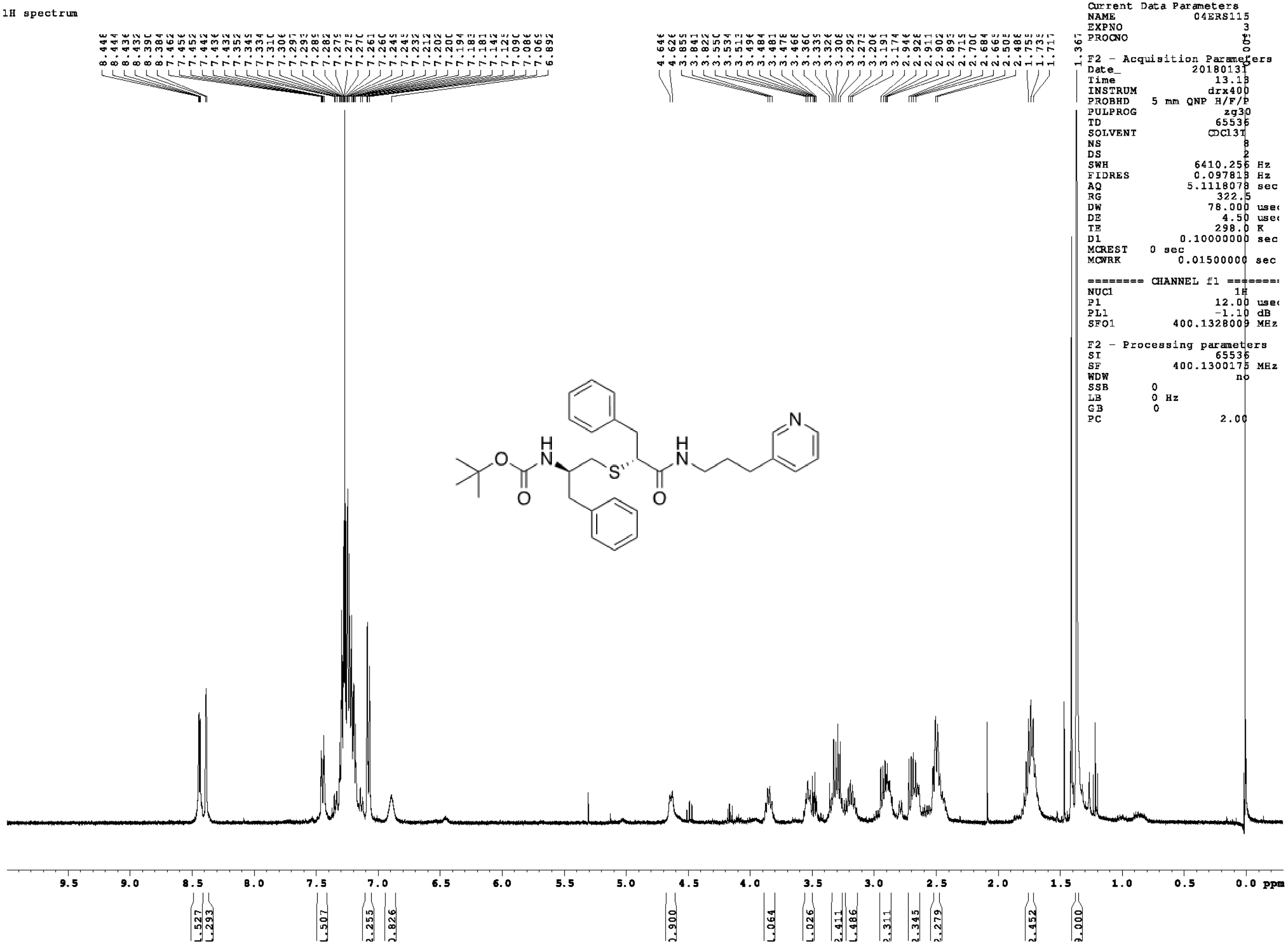

Current Data Parameters
NAME 04ERS115
EXPNO 3
PROCNO 1

F2 - Acquisition Parameters
Date_ 20180131
Time 13.13
INSTRUM drx400
PROBHD 5 mm QNP H/F/P
PULPROG zg30
TD 65536
SOLVENT CDCl3
NS 8
DS 2
SWH 6410.256 Hz
FIDRES 0.097813 Hz
AQ 5.1118078 sec
RG 322.5
DW 78.000 usec
DE 4.50 usec
TE 298.0 K
D1 0.10000000 sec
MCREST 0 sec
MCWRK 0.01500000 sec

======== CHANNEL f1 ========
NUC1 1H
P1 12.00 usec
PL1 -1.10 dB
SFO1 400.1328009 MHz

F2 - Processing parameters
SI 65536
SF 400.1300175 MHz
WDW no
SSB 0
LB 0 Hz
GB 0
PC 2.00

1H spectrum

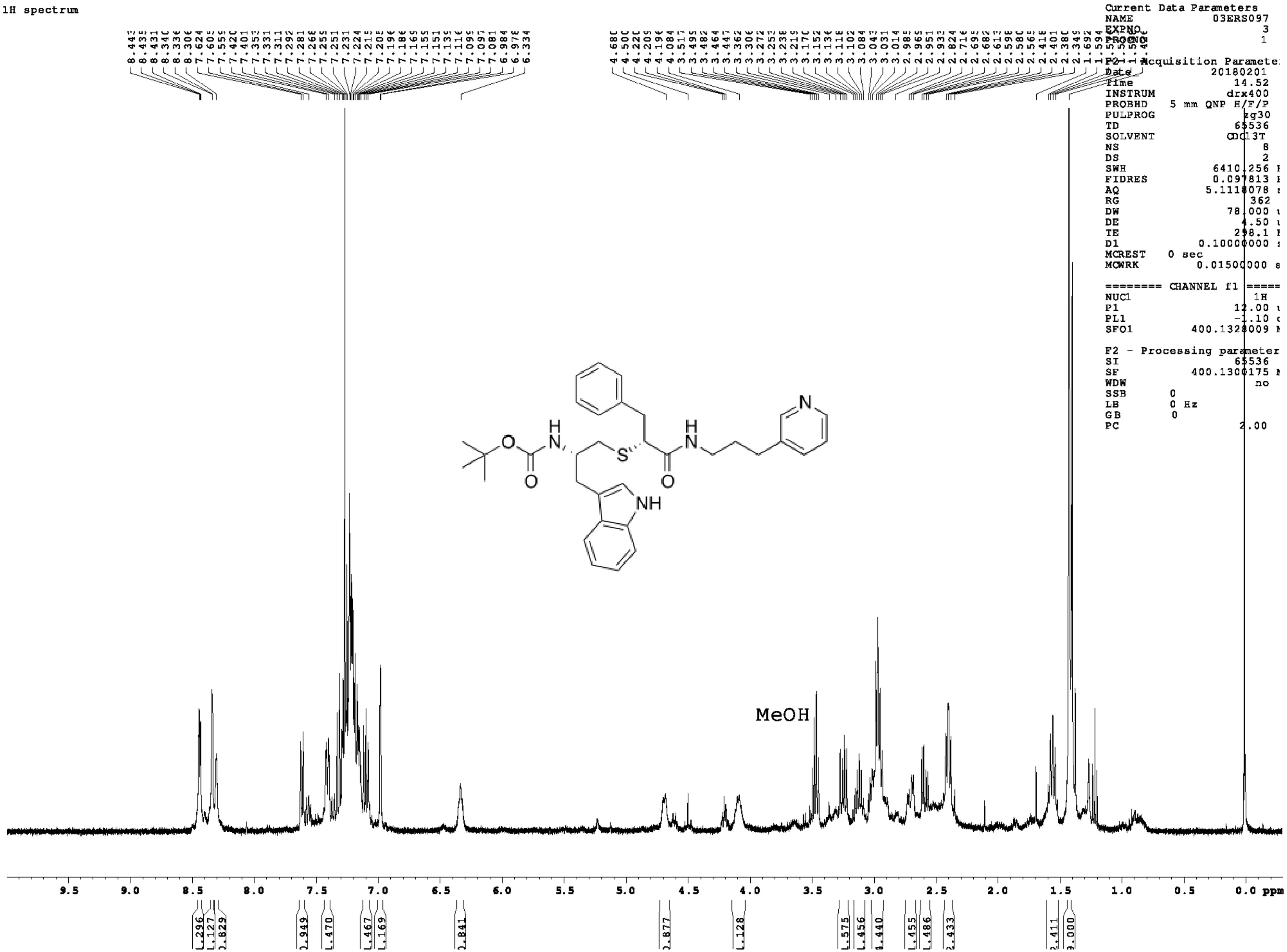

Current Data Parameters
NAME 03ERS097
EXPNO 3
PROCNO 1
F2 - Acquisition Paramete
Date_ 20180201
Time 14.52
INSTRUM drx400
PROBHD 5 mm QNP H/F/P
PULPROG zg30
TD 65536
SOLVENT CDCl3T
NS 8
DS 2
SWH 6410.256
FIDRES 0.097813
AQ 5.1118078
RG 362
DW 78.000
DE 4.50
TE 298.1
D1 0.10000000
MCREST 0 sec
MCWRK 0.01500000
======== CHANNEL f1 =====
NUC1 1H
P1 12.00
PL1 -1.10
SFO1 400.1328009
F2 - Processing parameter
SI 65536
SF 400.1300175
WDW no
SSB 0
LB 0 Hz
GB 0
PC 2.00
MeOH
ppm

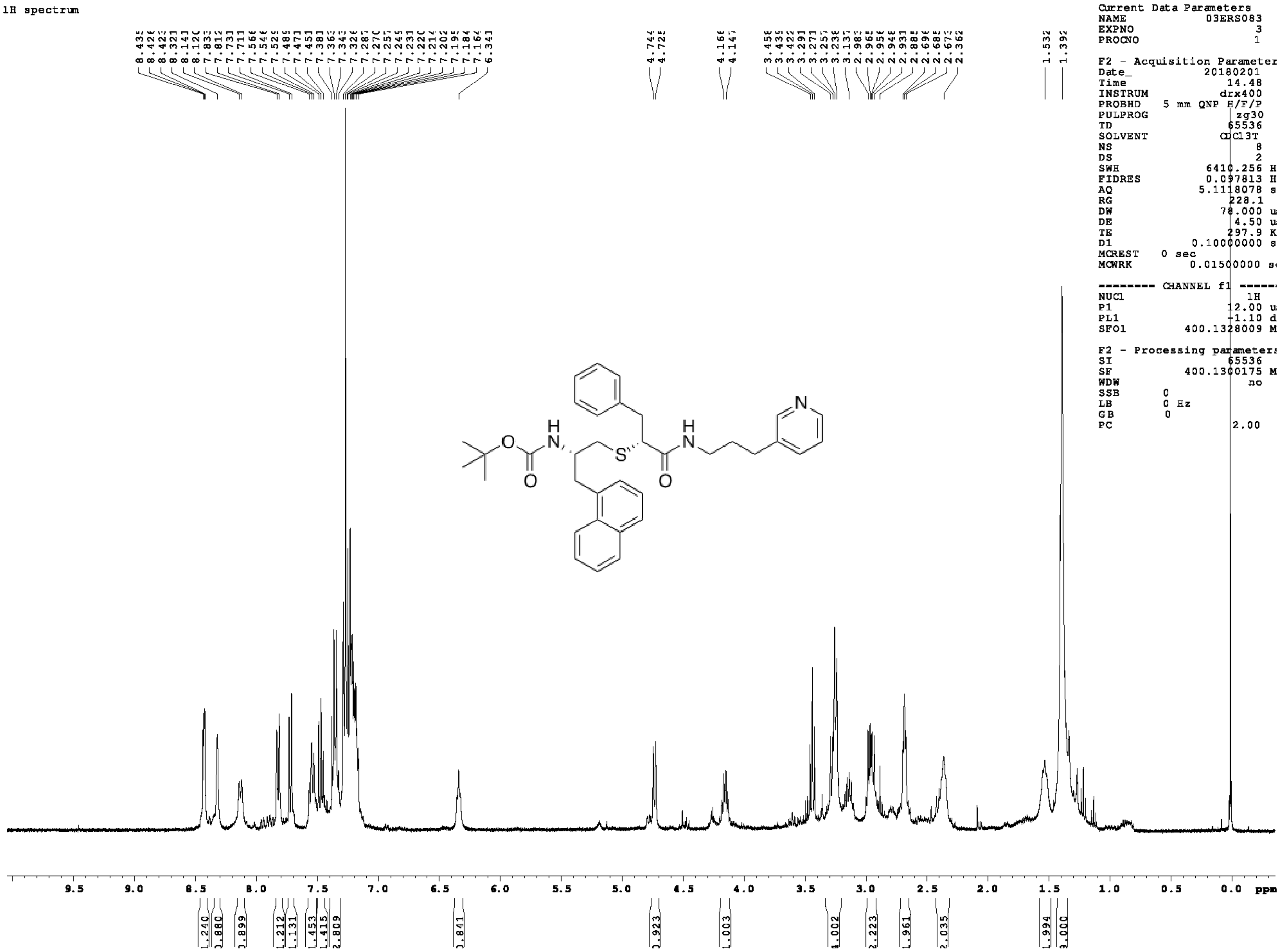
1H spectrum
Current Data Parameters
NAME 03ERS083
EXPNO 3
PROCNO 1
F2 - Acquisition Parameter
Date_ 20180201
Time 14.48
INSTRUM drx400
PROBHD 5 mm QNP H/F/P
PULPROG zg30
TD 65536
SOLVENT CDCl3T
NS 8
DS 2
SWH 6410.256 H
FIDRES 0.097813 H
AQ 5.1118078 s
RG 228.1
DW 78.000 u
DE 4.50 u
TE 297.9 K
D1 0.10000000 s
MCREST 0 sec
MCWRK 0.01500000 s
======== CHANNEL f1 ========
NUC1 1H
P1 12.00 u
PL1 -1.10 d
SFO1 400.1328009 M
F2 - Processing parameters
SI 65536
SF 400.1300175 M
WDW no
SSB 0
LB 0 Hz
GB 0
PC 2.00
9.5 9.0 8.5 8.0 7.5 7.0 6.5 6.0 5.5 5.0 4.5 4.0 3.5 3.0 2.5 2.0 1.5 1.0 0.5 0.0 ppm

1H spectrum

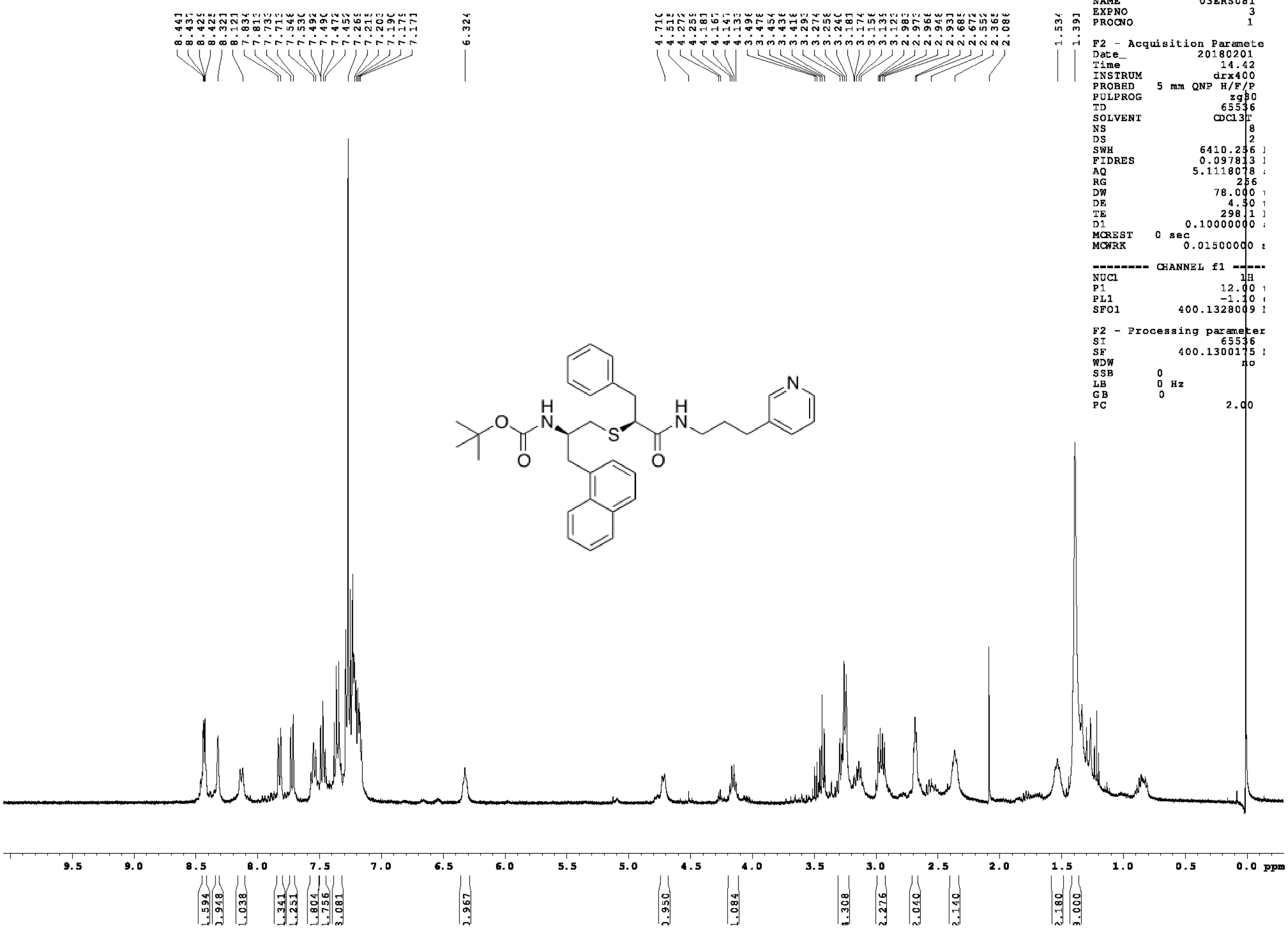


```
Current Data Parameters
NAME          03ERS081
EXPNO                3
PROCNO               1

F2 - Acquisition Paramete
Date_         20180201
Time             14.42
INSTRUM         drx400
PROBHD   5 mm QNP H/F/P
PULPROG           zg30
TD               65536
SOLVENT          CDCl3
NS                   8
DS                   2
SWH           6410.256
FIDRES        0.097813
AQ           5.1118078
RG                 256
DW              78.000
DE                4.50
TE               298.1
D1          0.10000000
MCREST   0 sec
MCWRK       0.01500000

======== CHANNEL f1 =====
NUC1                1H
P1               12.00
PL1              -1.10
SFO1       400.1328009

F2 - Processing parameter
SI               65536
SF         400.1300175
WDW                 no
SSB      0
LB       0 Hz
GB       0
PC                2.00
```

1H spectrum

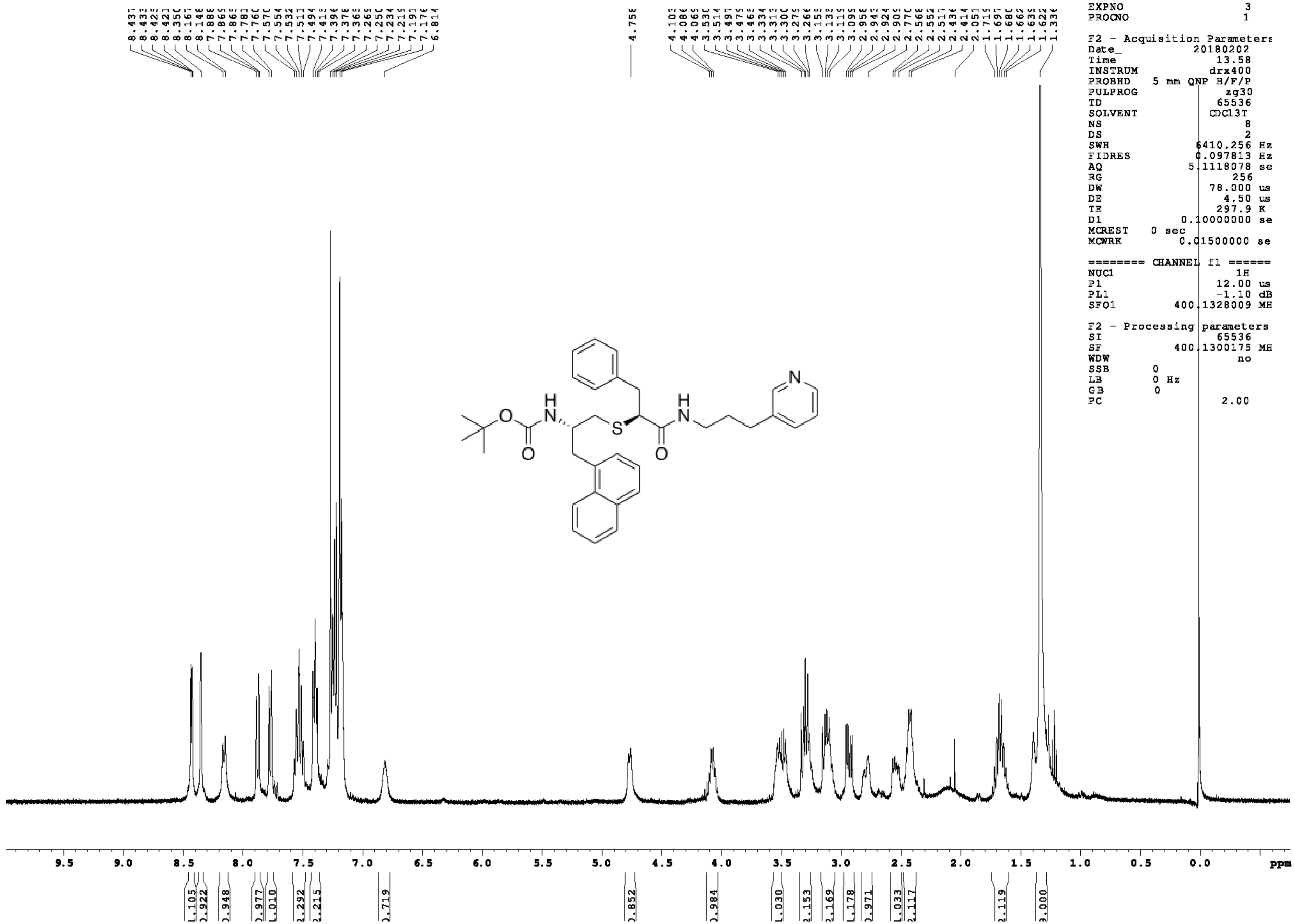

Current Data Parameters
NAME 04ERS129
EXPNO 3
PROCNO 1

F2 - Acquisition Parameters
Date_ 20180202
Time 13.58
INSTRUM drx400
PROBHD 5 mm QNP H/F/P
PULPROG zg30
TD 65536
SOLVENT CDCl3T
NS 8
DS 2
SWH 6410.256 Hz
FIDRES 0.097813 Hz
AQ 5.1118078 se
RG 256
DW 78.000 us
DE 4.50 us
TE 297.9 K
D1 0.10000000 se
MCREST 0 sec
MCWRK 0.01500000 se

======== CHANNEL f1 ======
NUC1 1H
P1 12.00 us
PL1 -1.10 dB
SFO1 400.1328009 MH

F2 - Processing parameters
SI 65536
SF 400.1300175 MH
WDW no
SSB 0
LB 0 Hz
GB 0
PC 2.00

1H spectrum

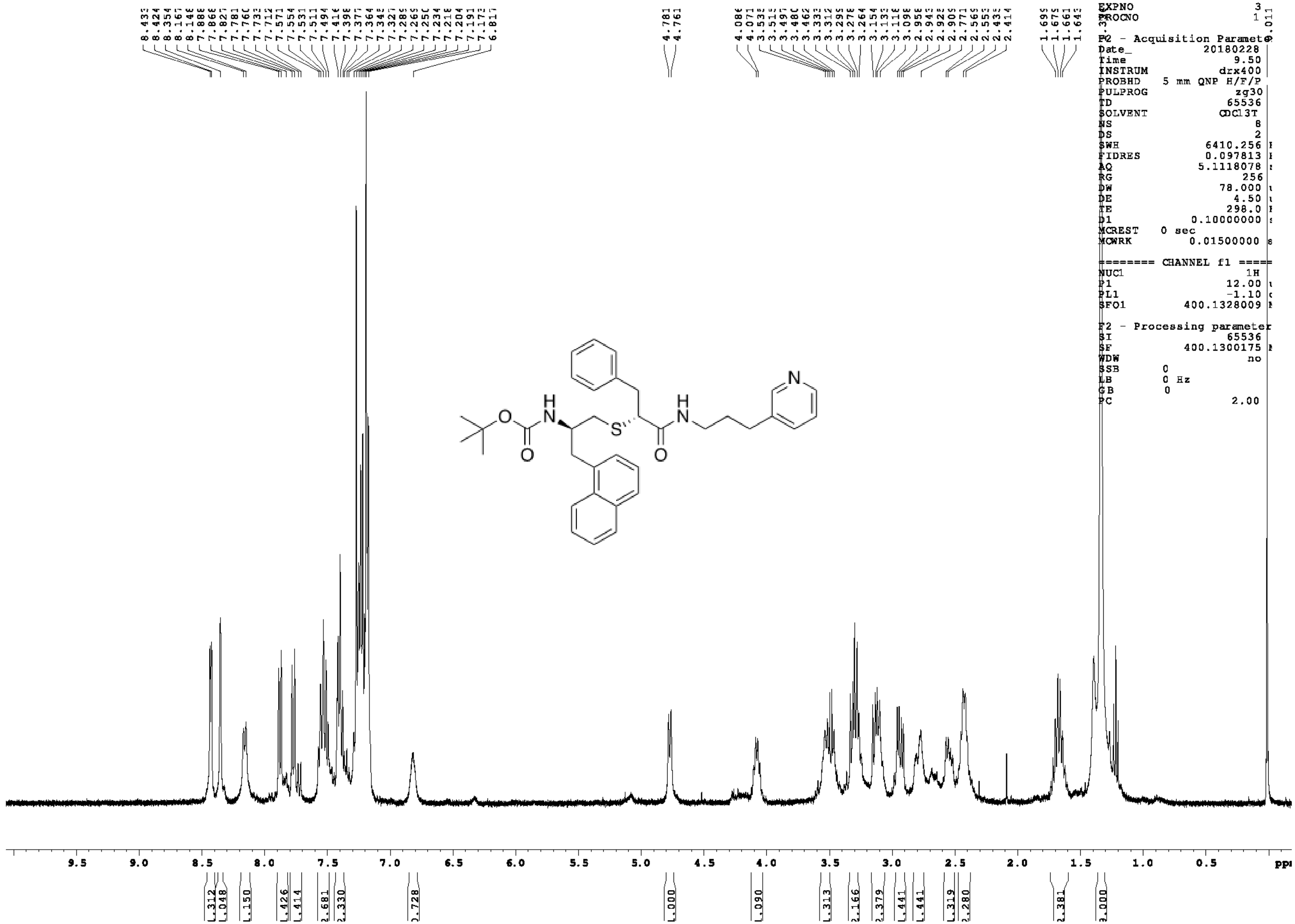
8.433
8.424
8.354
8.167
8.148
7.888
7.868
7.827
7.781
7.760
7.733
7.712
7.571
7.554
7.531
7.511
7.494
7.416
7.398
7.377
7.364
7.345
7.327
7.289
7.269
7.250
7.234
7.218
7.204
7.191
7.173
6.817
4.781
4.761
4.086
4.071
3.535
3.515
3.497
3.480
3.462
3.333
3.312
3.295
3.278
3.264
3.154
3.133
3.118
3.098
2.958
2.943
2.925
2.909
2.771
2.569
2.553
2.435
2.414
1.699
1.679
1.661
1.643
0.011
Current Data Parameters
NAME 04ERS145
EXPNO 3
PROCNO 1
F2 - Acquisition Paramete
Date_ 20180228
Time 9.50
INSTRUM drx400
PROBHD 5 mm QNP H/F/P
PULPROG zg30
TD 65536
SOLVENT CDCl3T
NS 8
DS 2
SWH 6410.256
FIDRES 0.097813
AQ 5.1118078
RG 256
DW 78.000
DE 4.50
TE 298.0
D1 0.10000000
MCREST 0 sec
MCWRK 0.01500000
======= CHANNEL f1 =====
NUC1 1H
P1 12.00
PL1 -1.10
SFO1 400.1328009
F2 - Processing parameter
SI 65536
SF 400.1300175
WDW no
SSB 0
LB 0 Hz
GB 0
PC 2.00
O
H
N
S
O
H
N
O
N
9.5
9.0
8.5
8.0
7.5
7.0
6.5
6.0
5.5
5.0
4.5
4.0
3.5
3.0
2.5
2.0
1.5
1.0
0.5
ppm
1.312
1.048
1.150
1.426
1.414
2.681
2.330
0.728
1.000
1.090
1.313
2.166
2.379
1.441
1.441
1.319
2.280
2.381
3.000

1H spectrum

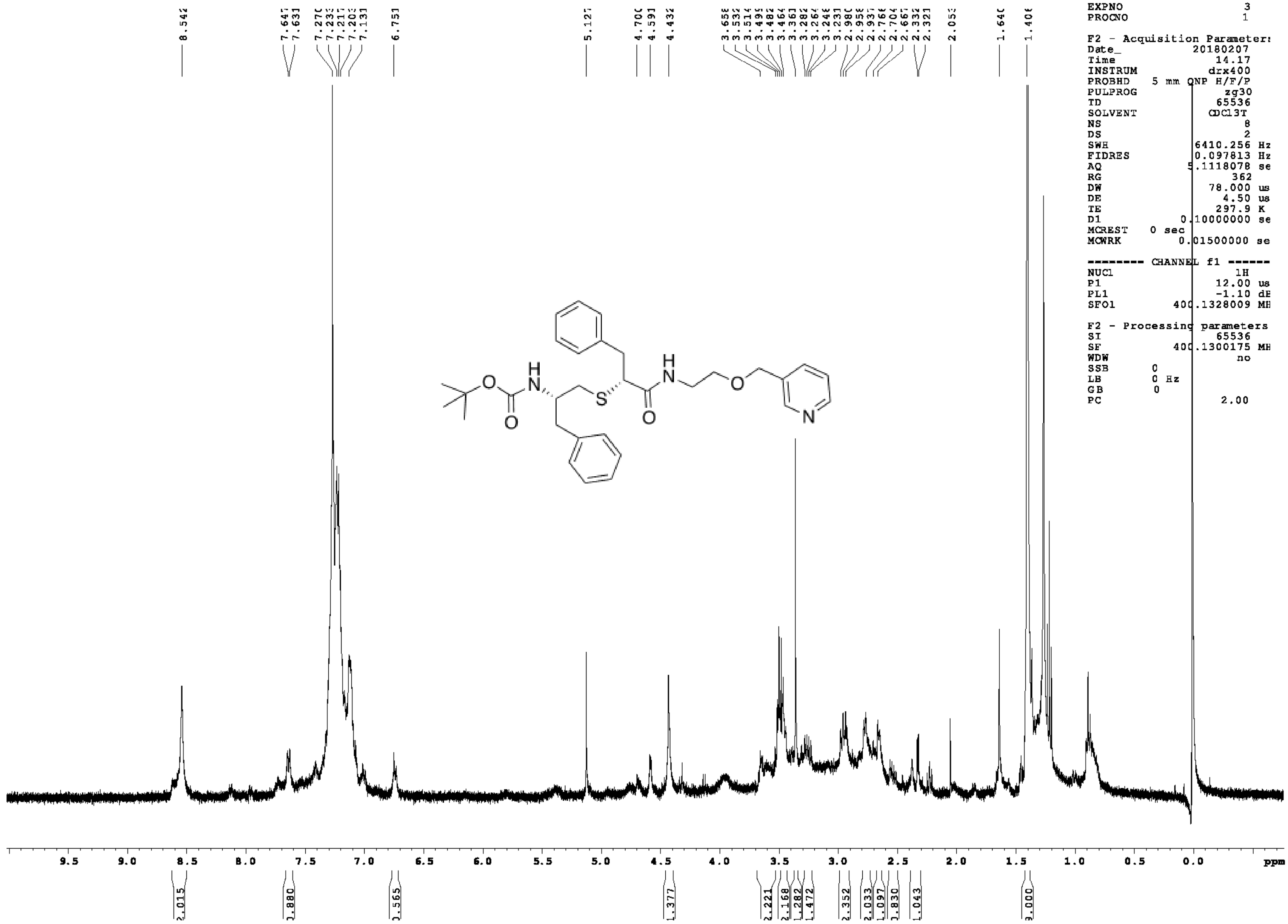

Current Data Parameters
NAME 04ERS102
EXPNO 3
PROCNO 1

F2 - Acquisition Parameters
Date_ 20180207
Time 14.17
INSTRUM drx400
PROBHD 5 mm QNP H/F/P
PULPROG zg30
TD 65536
SOLVENT CDCl3T
NS 8
DS 2
SWH 6410.256 Hz
FIDRES 0.097813 Hz
AQ 5.1118078 se
RG 362
DW 78.000 us
DE 4.50 us
TE 297.9 K
D1 0.10000000 se
MCREST 0 sec
MCWRK 0.01500000 se

======== CHANNEL f1 ======
NUC1 1H
P1 12.00 us
PL1 -1.10 dB
SFO1 400.1328009 MH

F2 - Processing parameters
SI 65536
SF 400.1300175 MH
WDW no
SSB 0
LB 0 Hz
GB 0
PC 2.00

1H spectrum

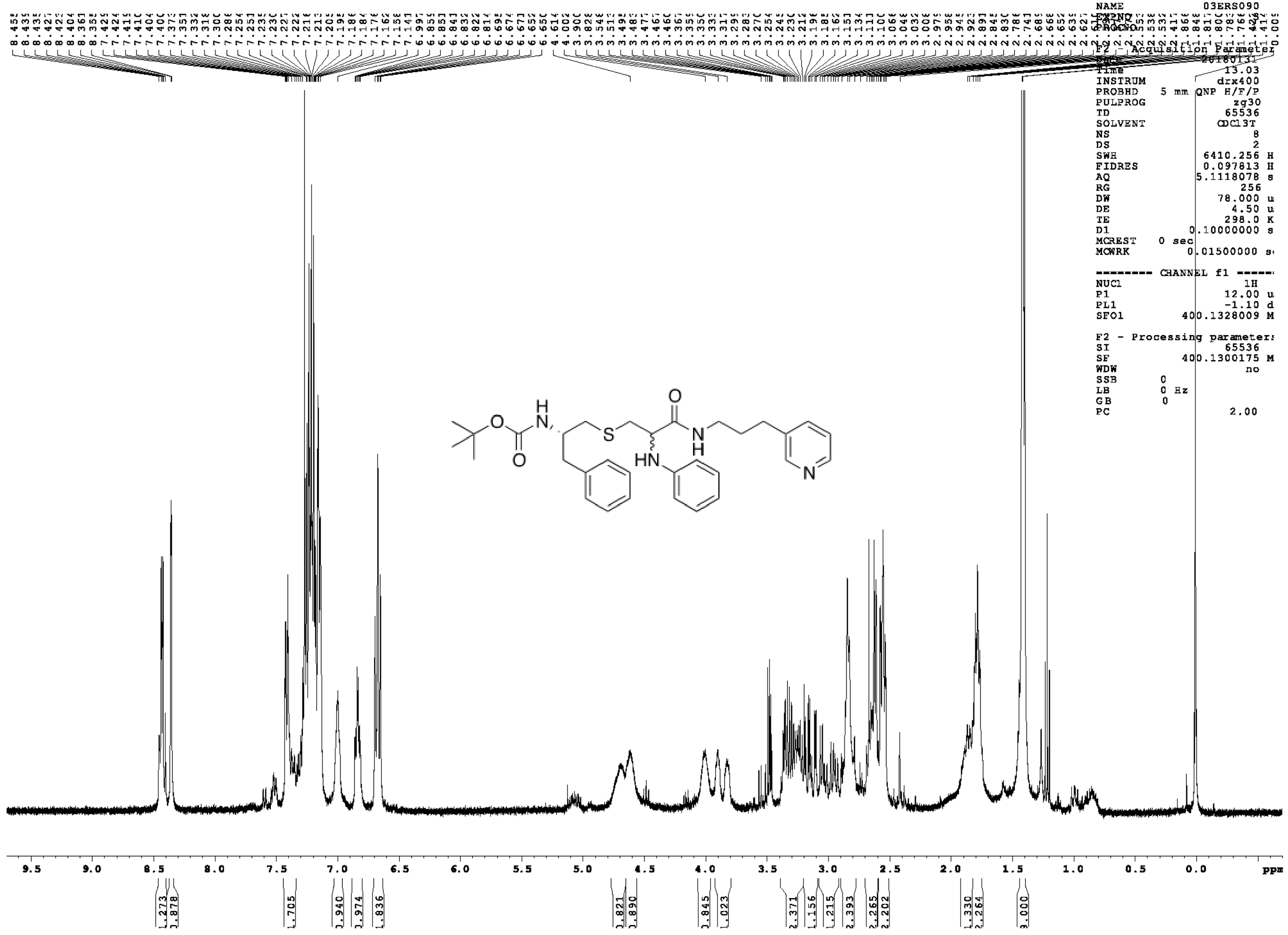

Current Data Parameters
NAME 03ERS090
F2 - Acquisition Parameters
Time 13.03
INSTRUM drx400
PROBHD 5 mm QNP H/F/P
PULPROG zg30
TD 65536
NS 8
DS 2
SWH 6410.256 H
FIDRES 0.097813 H
AQ 5.1118078 s
RG 256
DW 78.000 u
DE 4.50 u
TE 298.0 K
D1 0.10000000 s
MCREST 0 sec
MCWRK 0.01500000 s
CHANNEL f1
NUC1 1H
P1 12.00 u
PL1 -1.10 d
SFO1 400.1328009 M
F2 - Processing parameters
SI 65536
SF 400.1300175 M
WDW no
SSB 0
LB 0 Hz
GB 0
PC 2.00
9.5 9.0 8.5 8.0 7.5 7.0 6.5 6.0 5.5 5.0 4.5 4.0 3.5 3.0 2.5 2.0 1.5 1.0 0.5 0.0 ppm

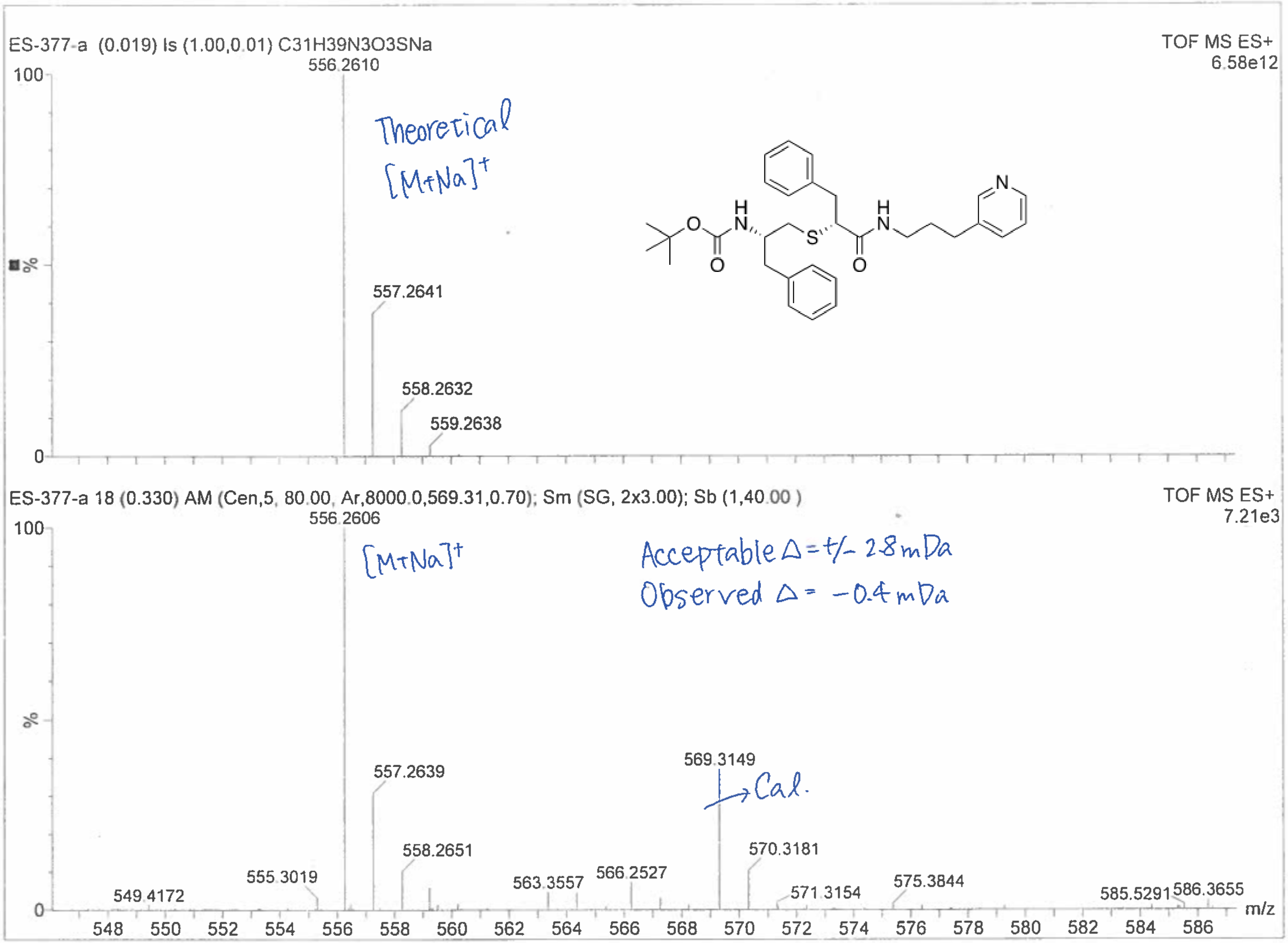
ES-377-a (0.019) Is (1.00,0.01) C31H39N3O3SNa
TOF MS ES+
6.58e12
556.2610
557.2641
558.2632
559.2638
Theoretical [M+Na]+
O
N
H
S
O
O
H
N
N
ES-377-a 18 (0.330) AM (Cen,5, 80.00, Ar,8000.0,569.31,0.70); Sm (SG, 2x3.00); Sb (1,40.00)
TOF MS ES+
7.21e3
556.2606
[M+Na]+
Acceptable Δ = +/- 2.8 mDa
Observed Δ = -0.4 mDa
557.2639
558.2651
555.3019
549.4172
563.3557
566.2527
569.3149
→ Cal.
570.3181
571.3154
575.3844
585.5291
586.3655
100
%
0
548
550
552
554
556
558
560
562
564
566
568
570
572
574
576
578
580
582
584
586
m/z

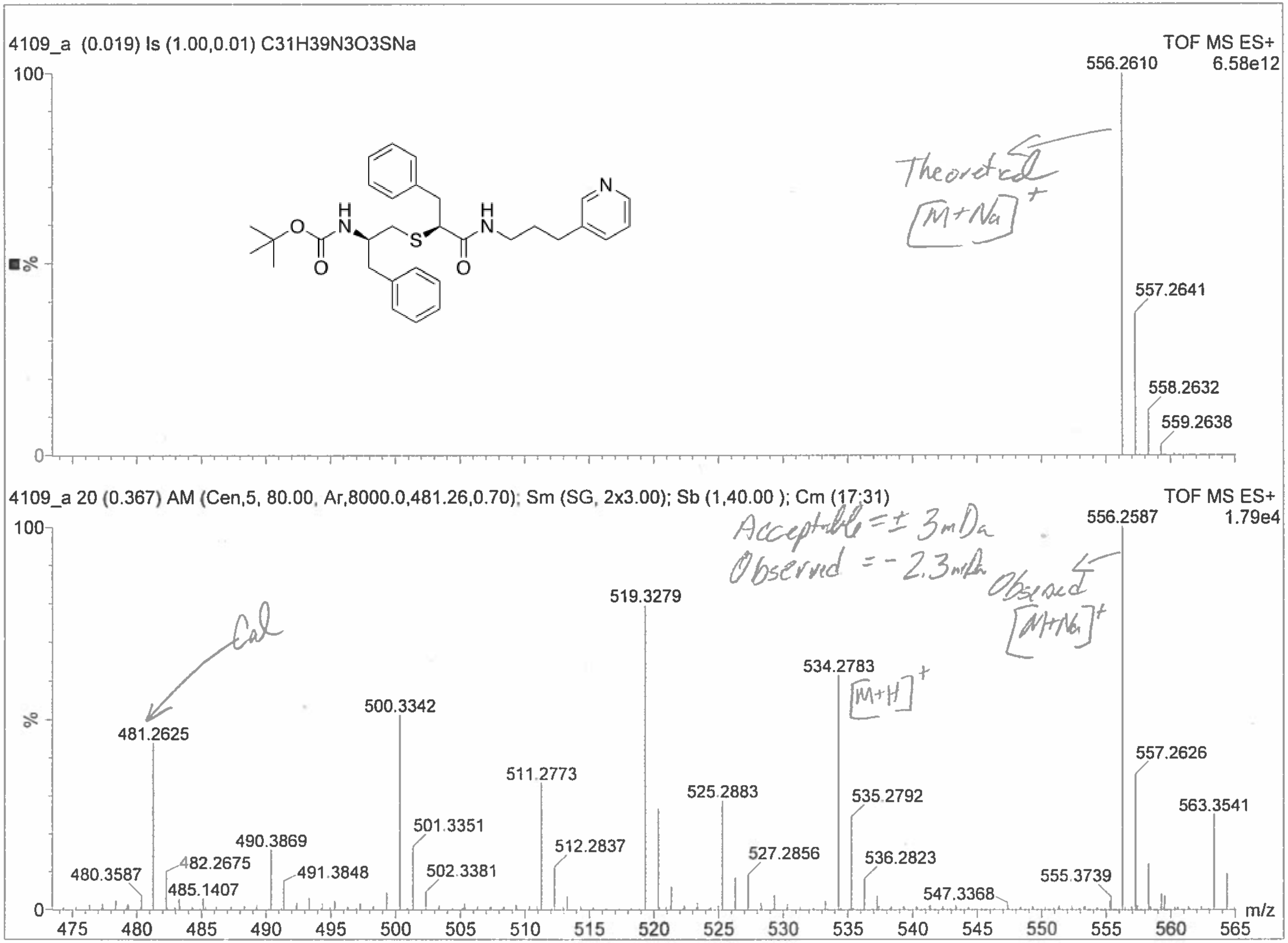
4109_a (0.019) Is (1.00,0.01) C31H39N3O3SNa
TOF MS ES+
556.2610
6.58e12
100
%
0
557.2641
558.2632
559.2638
Theoretical
[M+Na]+
4109_a 20 (0.367) AM (Cen,5, 80.00, Ar,8000.0,481.26,0.70); Sm (SG, 2x3.00); Sb (1,40.00); Cm (17:31)
TOF MS ES+
556.2587
1.79e4
Acceptable = ± 3mDa
Observed = -2.3mDa
Observed
[M+Na]+
Cal
519.3279
534.2783
[M+H]+
500.3342
481.2625
511.2773
525.2883
535.2792
557.2626
563.3541
501.3351
512.2837
527.2856
536.2823
490.3869
482.2675
480.3587
491.3848
502.3381
485.1407
547.3368
555.3739
475 480 485 490 495 500 505 510 515 520 525 530 535 540 545 550 555 560 565
m/z

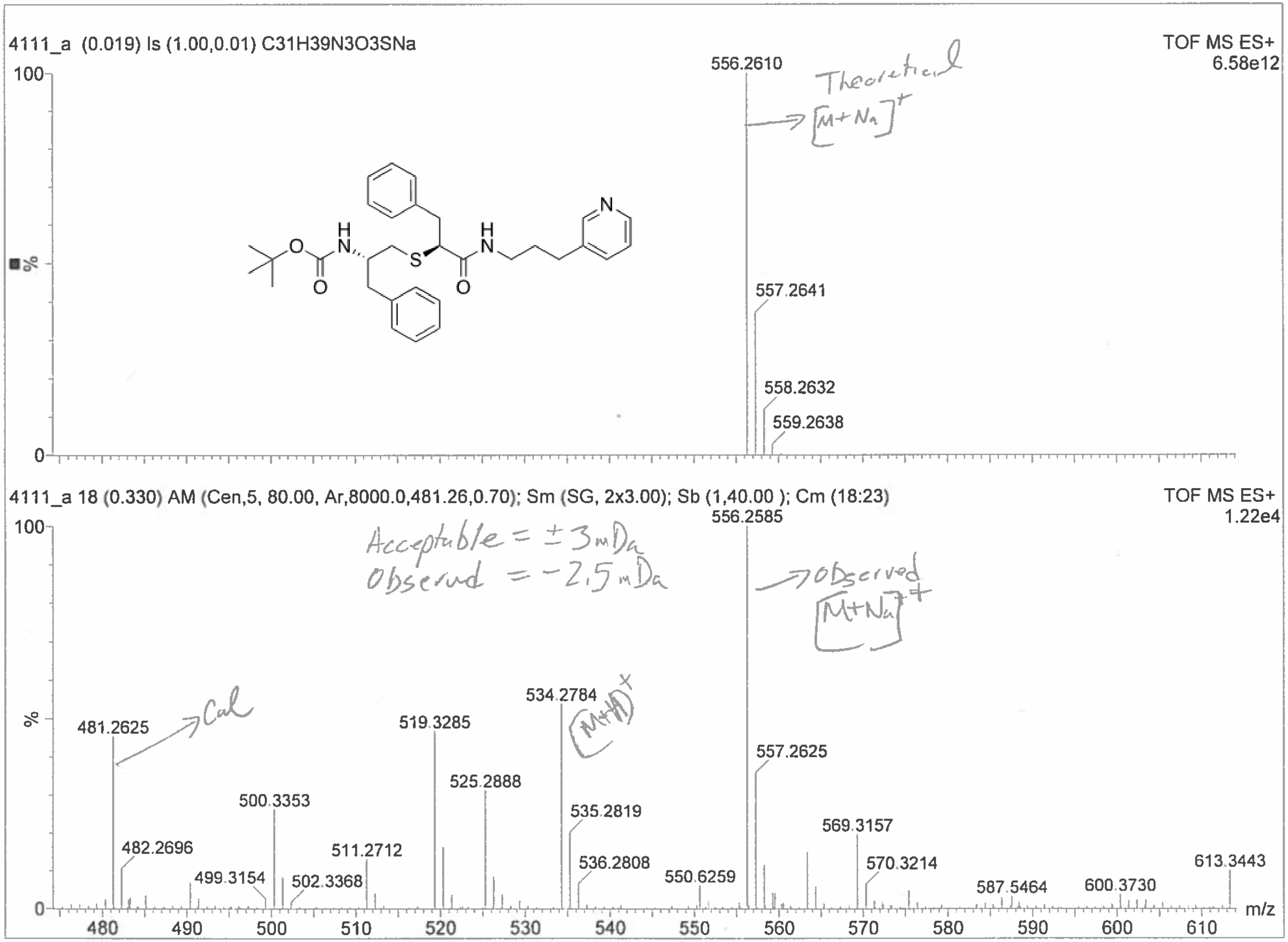
4111_a (0.019) Is (1.00,0.01) C31H39N3O3SNa
TOF MS ES+
6.58e12
556.2610
557.2641
558.2632
559.2638
Theoretical
[M+Na]+
4111_a 18 (0.330) AM (Cen,5, 80.00, Ar,8000.0,481.26,0.70); Sm (SG, 2x3.00); Sb (1,40.00); Cm (18:23)
TOF MS ES+
1.22e4
556.2585
Acceptable = ±3 mDa
Observed = -2.5 mDa
Observed
[M+Na]+
481.2625
Cal
482.2696
499.3154
500.3353
502.3368
511.2712
519.3285
525.2888
534.2784
(M+H)+
535.2819
536.2808
550.6259
557.2625
569.3157
570.3214
587.5464
600.3730
613.3443
m/z

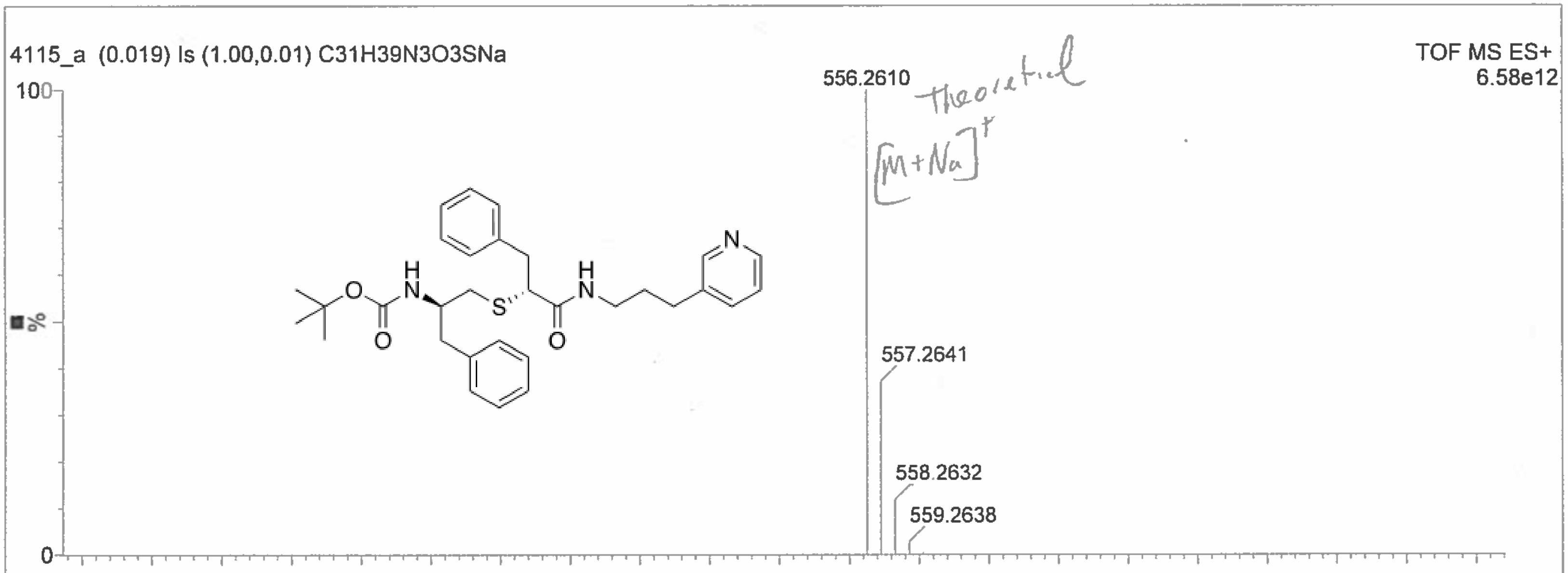
4115_a (0.019) Is (1.00,0.01) C31H39N3O3SNa
TOF MS ES+
6.58e12
100
%
0
556.2610
557.2641
558.2632
559.2638
theoretical
[M+Na]+

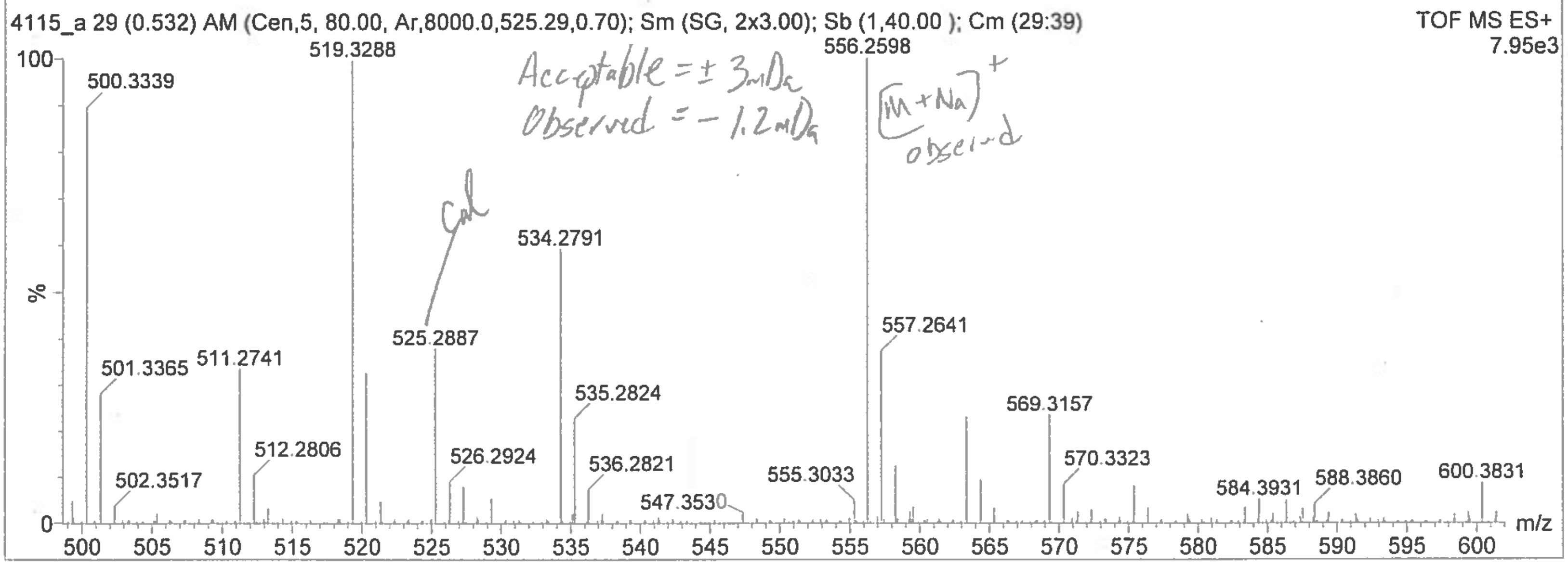
4115_a 29 (0.532) AM (Cen,5, 80.00, Ar,8000.0,525.29,0.70); Sm (SG, 2x3.00); Sb (1,40.00); Cm (29:39)
TOF MS ES+
7.95e3
100
%
0
500.3339
501.3365
502.3517
511.2741
512.2806
519.3288
525.2887
526.2924
534.2791
535.2824
536.2821
547.3530
555.3033
556.2598
557.2641
569.3157
570.3323
584.3931
588.3860
600.3831
500
505
510
515
520
525
530
535
540
545
550
555
560
565
570
575
580
585
590
595
600
m/z
Acceptable = ± 3mDa
Observed = −1.2mDa
Cal
[M+Na]+
observed

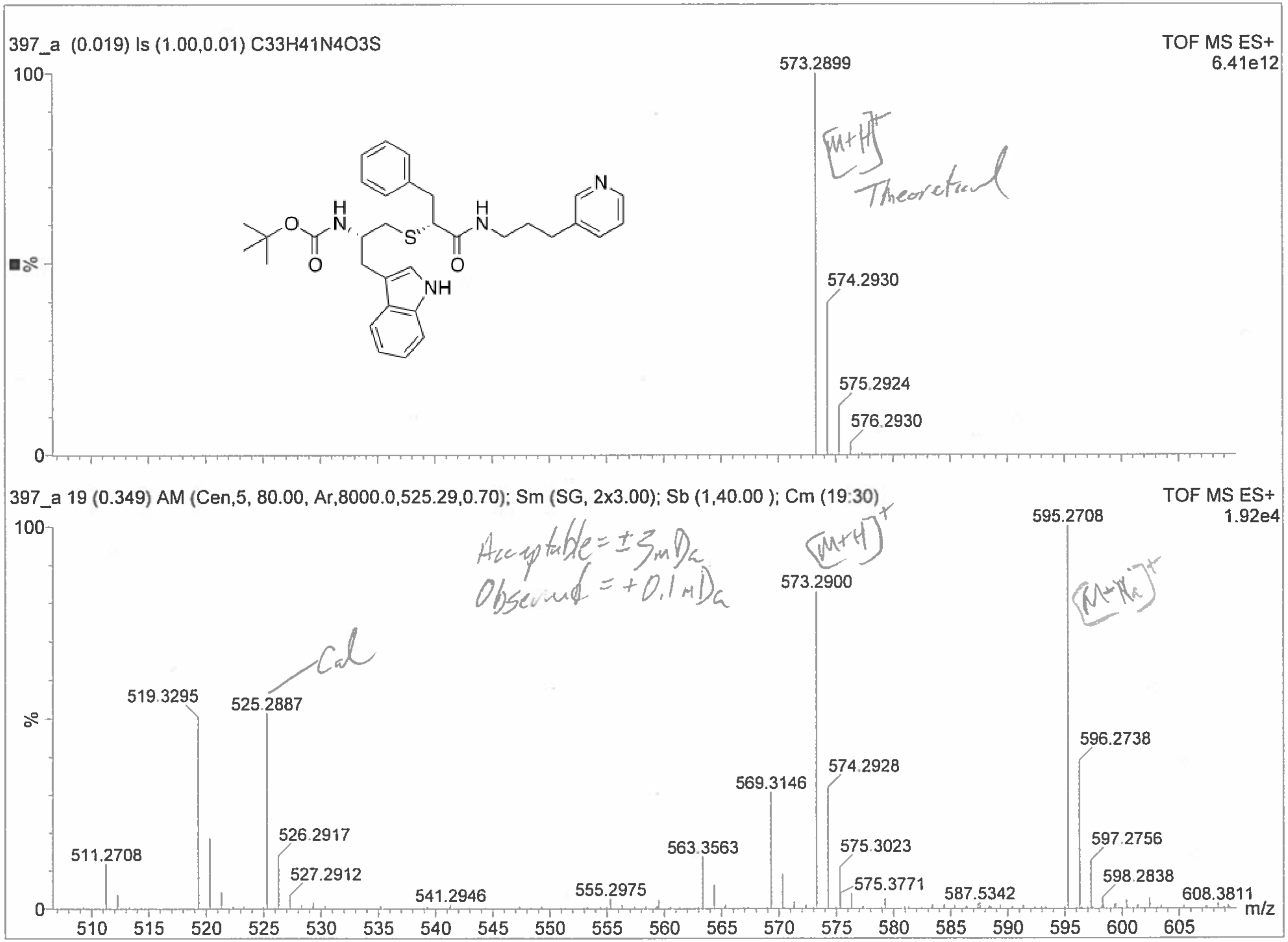
397_a (0.019) Is (1.00,0.01) C33H41N4O3S
TOF MS ES+
6.41e12
573.2899
574.2930
575.2924
576.2930
[M+H]+
Theoretical
397_a 19 (0.349) AM (Cen,5, 80.00, Ar,8000.0,525.29,0.70); Sm (SG, 2x3.00); Sb (1,40.00); Cm (19:30)
TOF MS ES+
1.92e4
Acceptable = ±3 mDa
Observed = +0.1 mDa
[M+H]+
[M+Na]+
Cal
595.2708
573.2900
519.3295
525.2887
596.2738
574.2928
569.3146
526.2917
511.2708
563.3563
575.3023
597.2756
527.2912
598.2838
575.3771
541.2946
555.2975
587.5342
608.3811
%
m/z

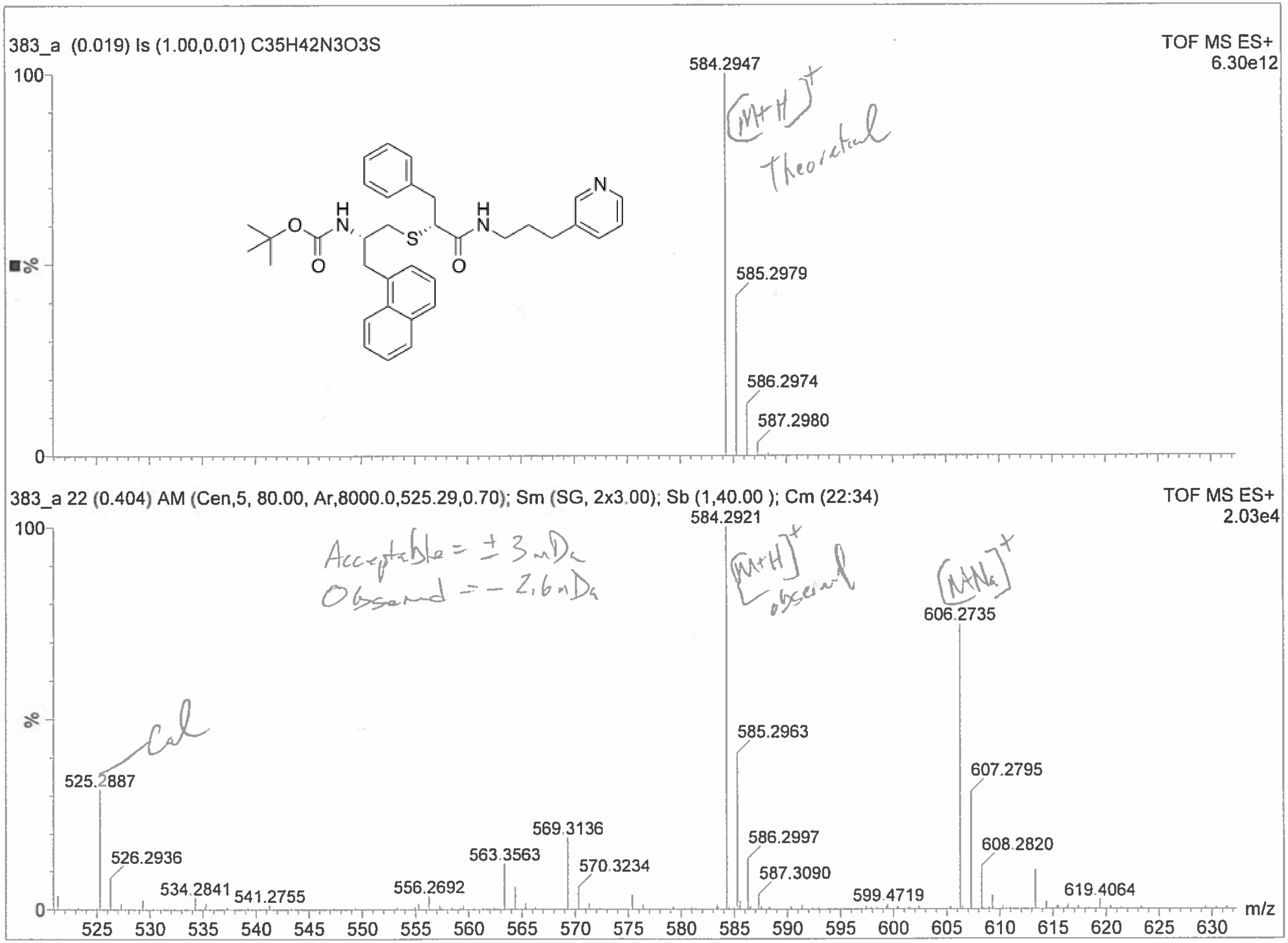
383_a (0.019) Is (1.00,0.01) C35H42N3O3S
TOF MS ES+
6.30e12
584.2947
[M+H]+
Theoretical
585.2979
586.2974
587.2980
N
H
N
O
O
S
H
N
O
100
%
0
383_a 22 (0.404) AM (Cen,5, 80.00, Ar,8000.0,525.29,0.70); Sm (SG, 2x3.00); Sb (1,40.00); Cm (22:34)
TOF MS ES+
2.03e4
584.2921
Acceptable = ± 3 mDa
Observed = − 2.6 mDa
[M+H]+
observed
[M+Na]+
606.2735
Cal
525.2887
526.2936
534.2841
541.2755
556.2692
563.3563
569.3136
570.3234
585.2963
586.2997
587.3090
599.4719
607.2795
608.2820
619.4064
m/z
525
530
535
540
545
550
555
560
565
570
575
580
585
590
595
600
605
610
615
620
625
630

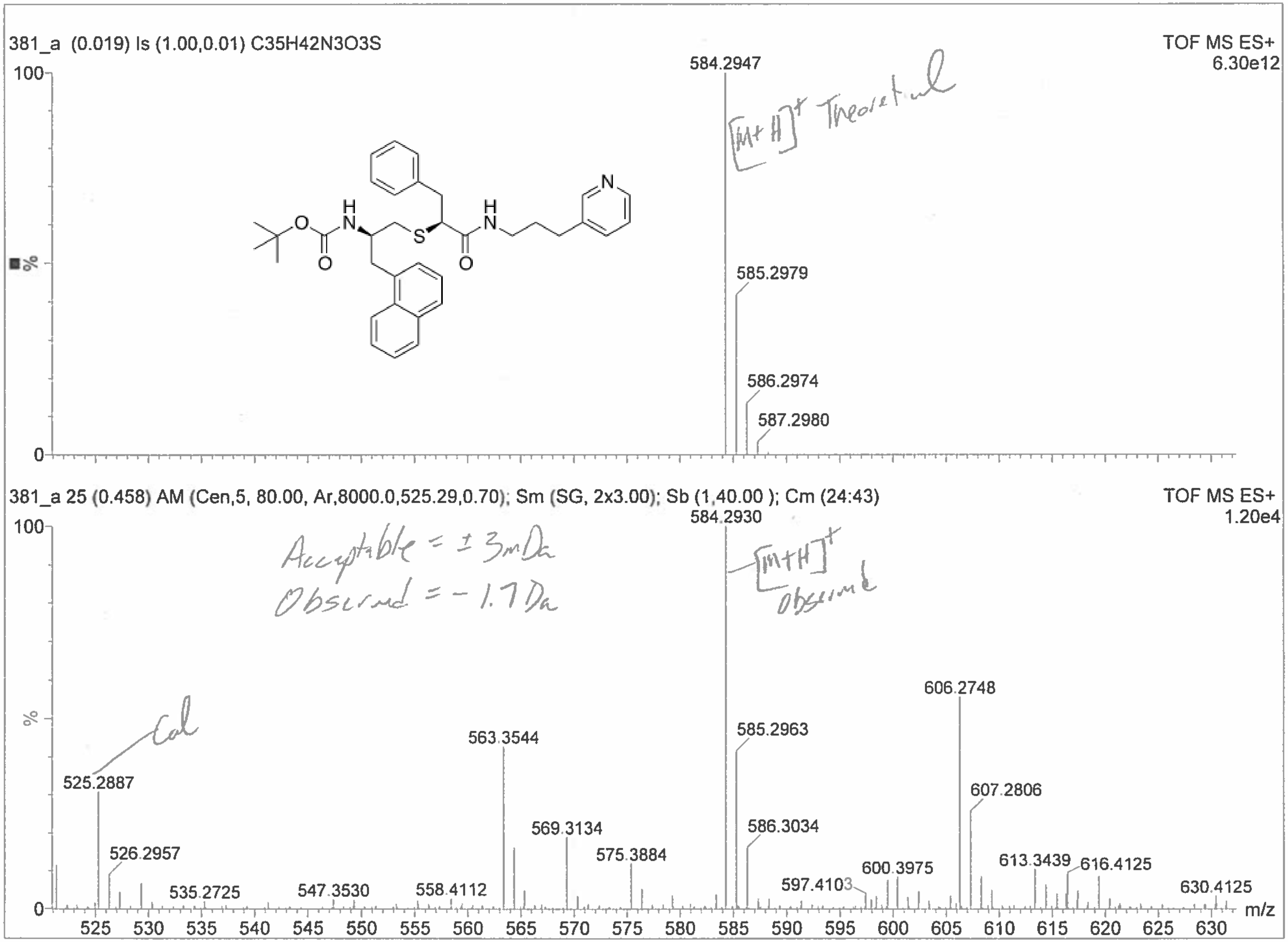
381_a (0.019) Is (1.00,0.01) C35H42N3O3S
TOF MS ES+
6.30e12
100
%
0
584.2947
585.2979
586.2974
587.2980
[M+H]+ Theoretical
381_a 25 (0.458) AM (Cen,5, 80.00, Ar,8000.0,525.29,0.70); Sm (SG, 2x3.00); Sb (1,40.00); Cm (24:43)
TOF MS ES+
1.20e4
584.2930
Acceptable = ± 3mDa
Observed = -1.7Da
[M+H]+ Observed
Cal
525.2887
526.2957
535.2725
547.3530
558.4112
563.3544
569.3134
575.3884
585.2963
586.3034
597.4103
600.3975
606.2748
607.2806
613.3439
616.4125
630.4125
525 530 535 540 545 550 555 560 565 570 575 580 585 590 595 600 605 610 615 620 625 630
m/z

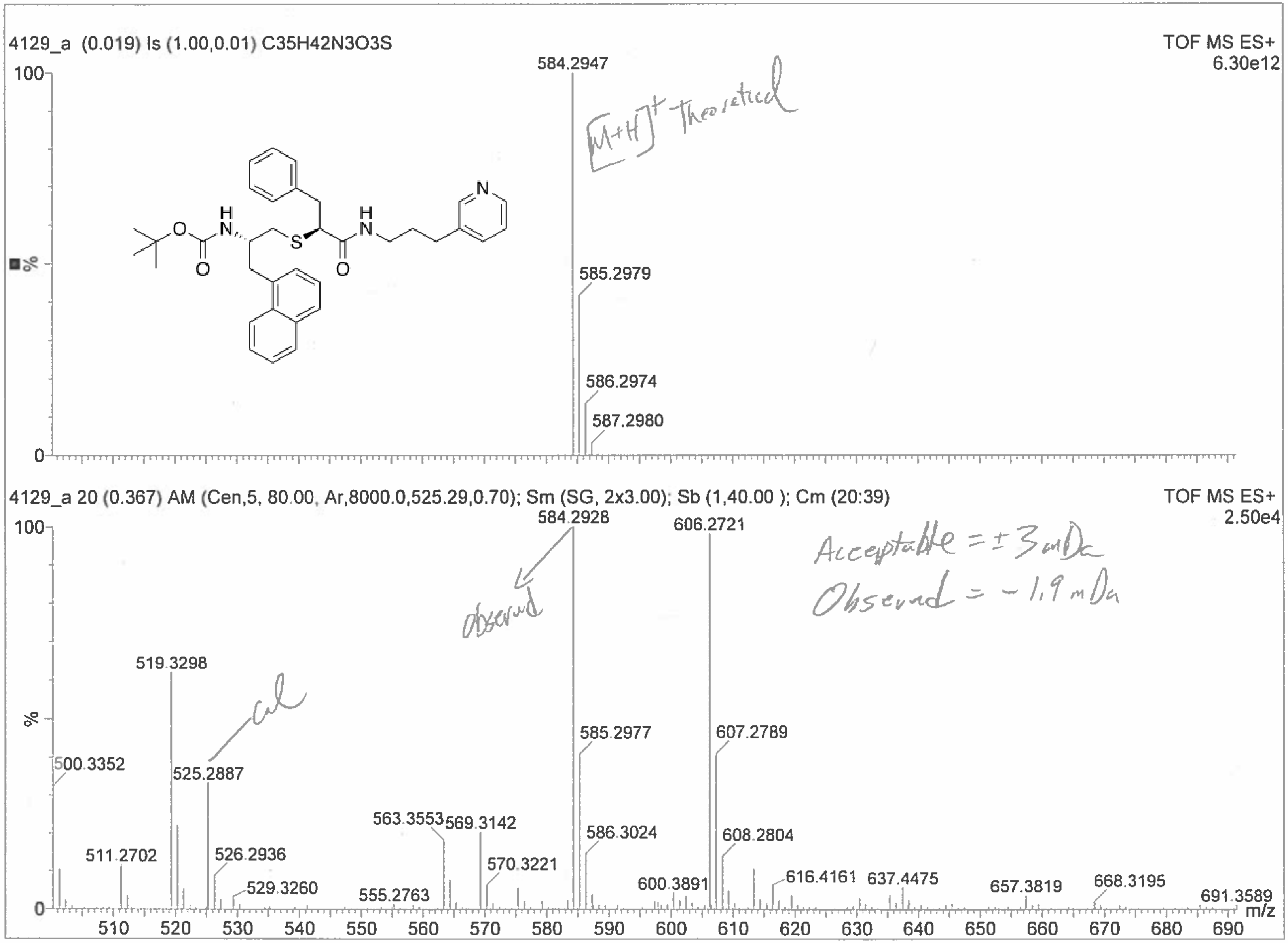
4129_a (0.019) Is (1.00,0.01) C35H42N3O3S
TOF MS ES+
6.30e12
584.2947
[M+H]+ Theoretical
585.2979
586.2974
587.2980
%
100
0
4129_a 20 (0.367) AM (Cen,5, 80.00, Ar,8000.0,525.29,0.70); Sm (SG, 2x3.00); Sb (1,40.00); Cm (20:39)
TOF MS ES+
2.50e4
584.2928
606.2721
Observed
Acceptable = ± 3 mDa
Observed = -1.9 mDa
519.3298
cal
500.3352
525.2887
585.2977
607.2789
511.2702
526.2936
529.3260
555.2763
563.3553
569.3142
570.3221
586.3024
600.3891
608.2804
616.4161
637.4475
657.3819
668.3195
691.3589
m/z
510 520 530 540 550 560 570 580 590 600 610 620 630 640 650 660 670 680 690

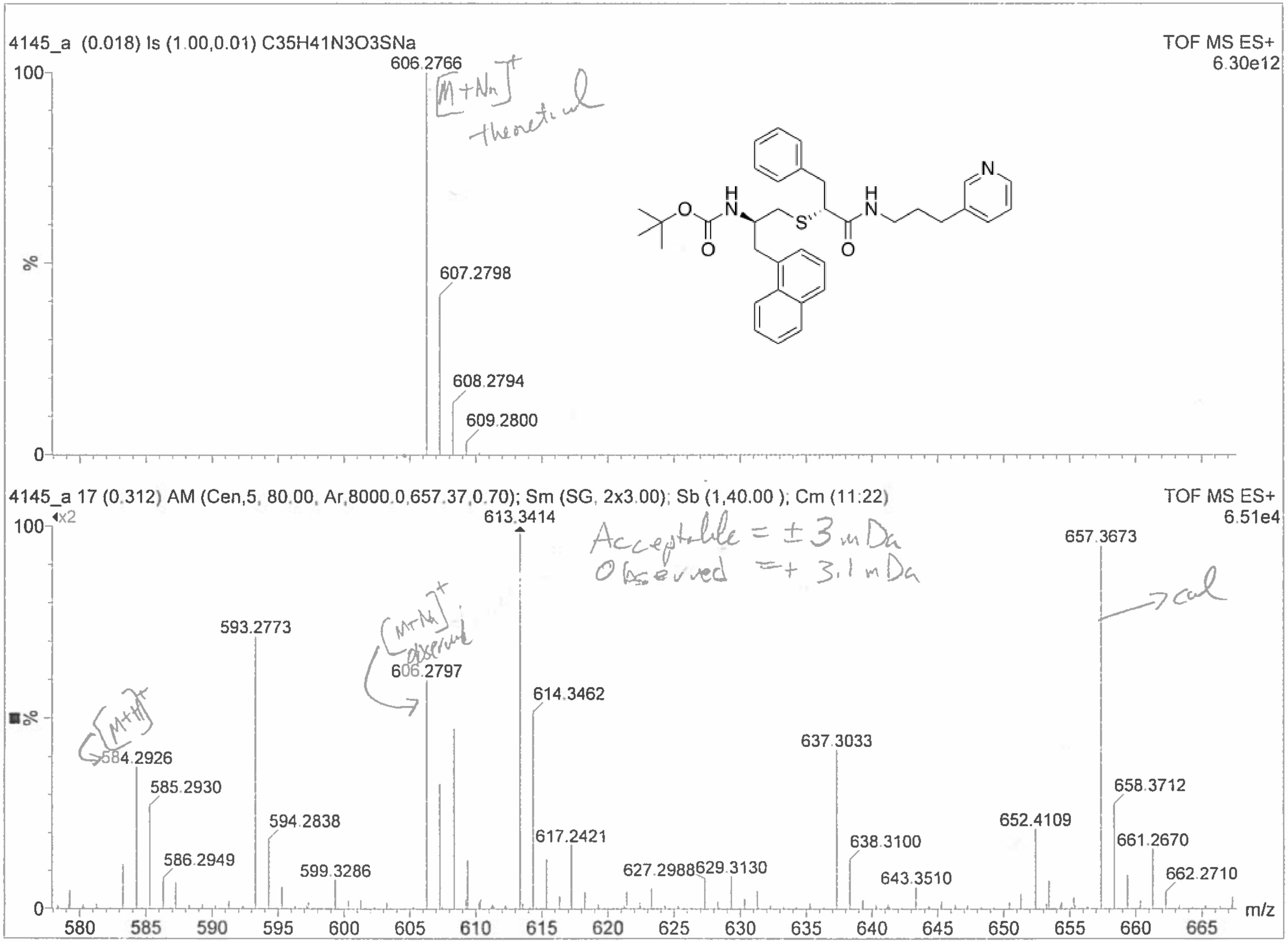
4145_a (0.018) Is (1.00,0.01) C35H41N3O3SNa
TOF MS ES+
6.30e12
606.2766
607.2798
608.2794
609.2800
[M+Na]+ theoretical
4145_a 17 (0.312) AM (Cen,5, 80.00, Ar,8000.0,657.37,0.70); Sm (SG, 2x3.00); Sb (1,40.00); Cm (11:22)
TOF MS ES+
6.51e4
x2
613.3414
657.3673
Acceptable = ± 3 mDa
Observed = + 3.1 mDa
cal
593.2773
[M+Na]+ observed
606.2797
614.3462
[M+H]+
584.2926
585.2930
594.2838
586.2949
599.3286
617.2421
637.3033
658.3712
652.4109
638.3100
661.2670
627.2988
629.3130
643.3510
662.2710
m/z

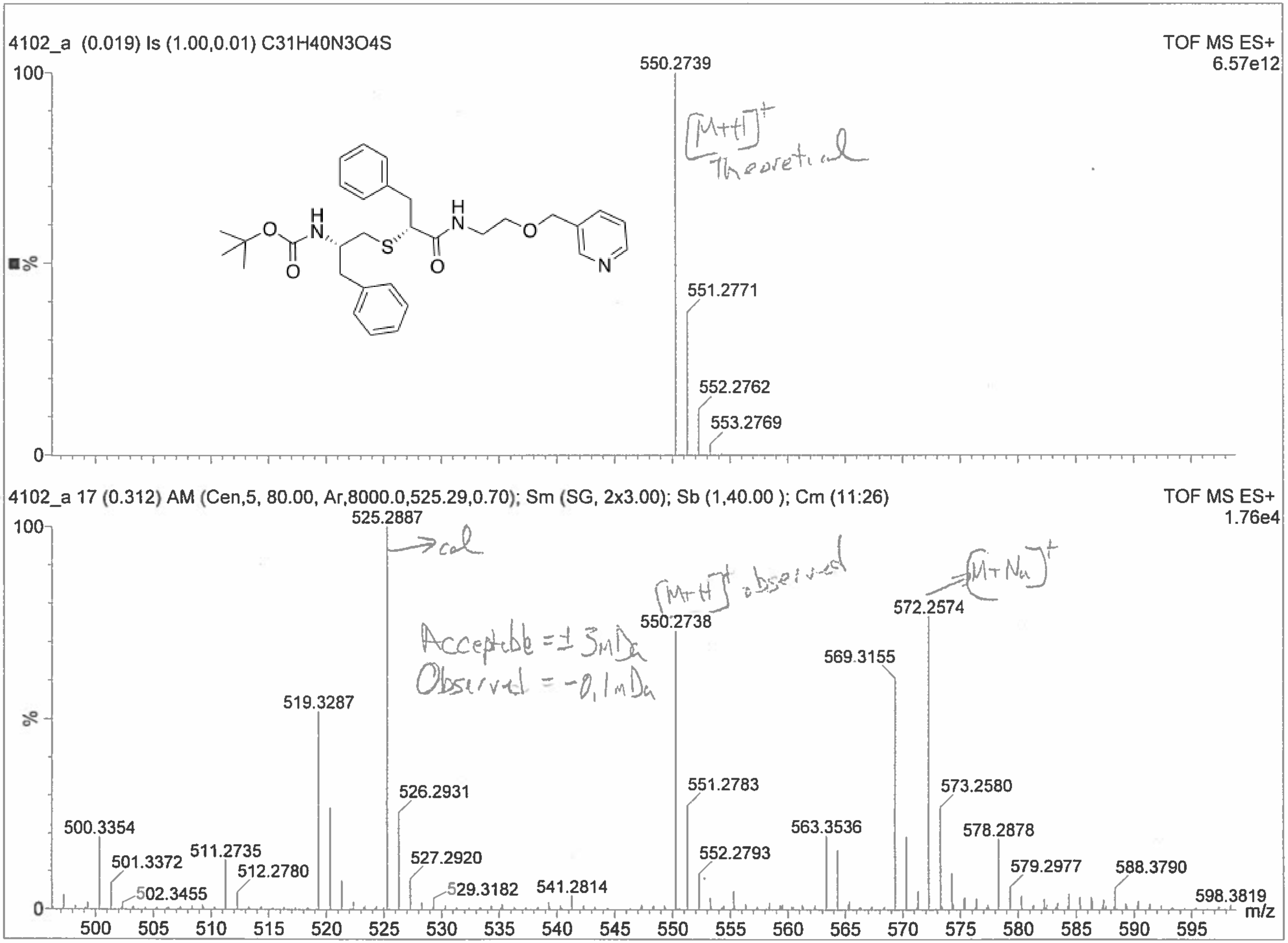
4102_a (0.019) Is (1.00,0.01) C31H40N3O4S
TOF MS ES+
6.57e12
550.2739
[M+H]+ Theoretical
551.2771
552.2762
553.2769
4102_a 17 (0.312) AM (Cen,5, 80.00, Ar,8000.0,525.29,0.70); Sm (SG, 2x3.00); Sb (1,40.00); Cm (11:26)
TOF MS ES+
1.76e4
525.2887
cal
[M+H]+ observed
[M+Na]+
572.2574
550.2738
Acceptable = ± 3mDa
Observed = -0.1mDa
569.3155
519.3287
526.2931
551.2783
573.2580
500.3354
563.3536
578.2878
511.2735
501.3372
552.2793
527.2920
512.2780
579.2977
588.3790
502.3455
541.2814
529.3182
598.3819
m/z
%
100
0
500 505 510 515 520 525 530 535 540 545 550 555 560 565 570 575 580 585 590 595

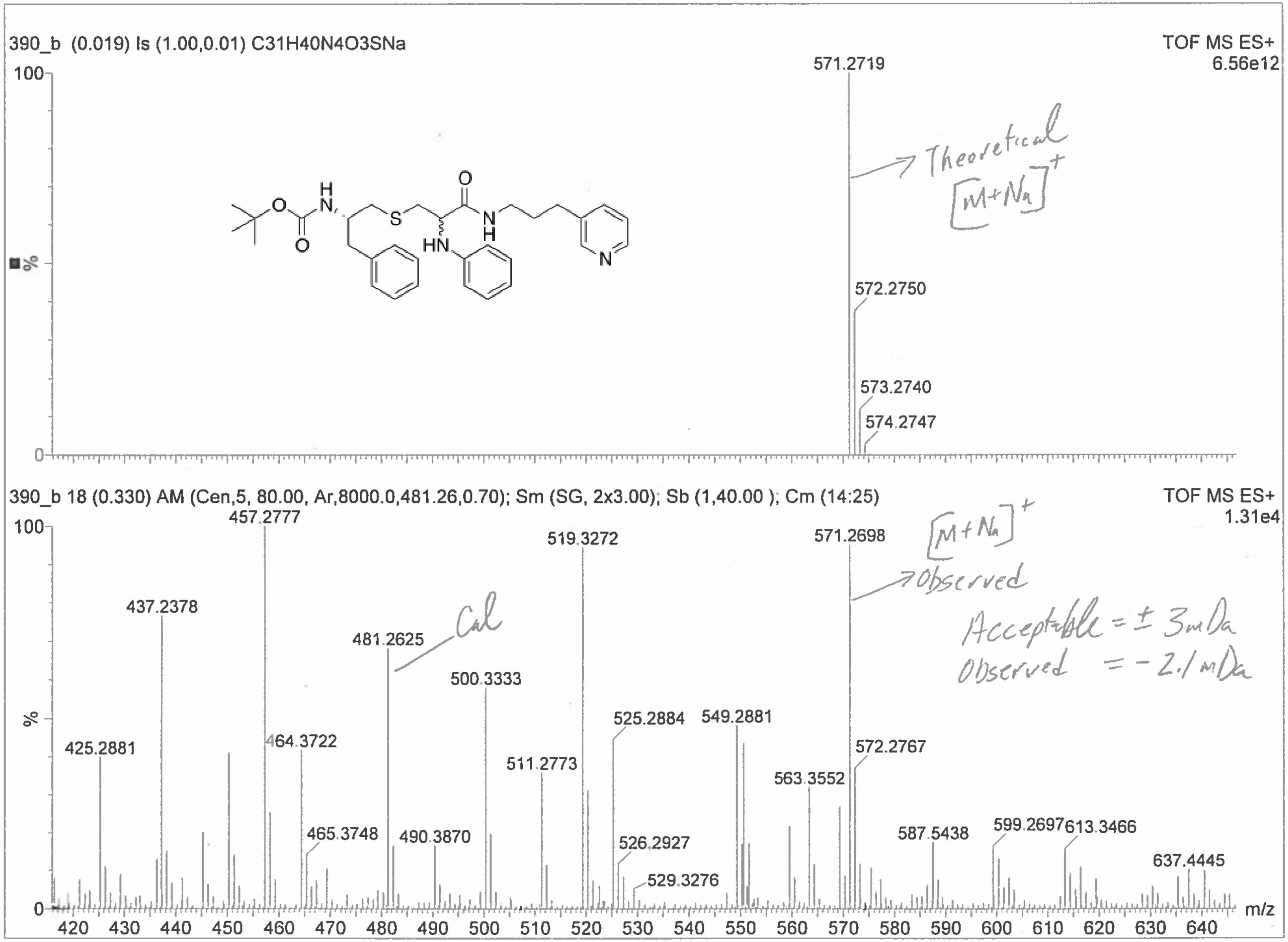

390_b (0.019) Is (1.00,0.01) C31H40N4O3SNa
TOF MS ES+
6.56e12
571.2719
572.2750
573.2740
574.2747
Theoretical [M+Na]+
390_b 18 (0.330) AM (Cen,5, 80.00, Ar,8000.0,481.26,0.70); Sm (SG, 2x3.00); Sb (1,40.00); Cm (14:25)
TOF MS ES+
1.31e4
457.2777
519.3272
571.2698
437.2378
481.2625
Cal
500.3333
525.2884
549.2881
425.2881
464.3722
511.2773
572.2767
563.3552
465.3748
490.3870
526.2927
587.5438
599.2697
613.3466
637.4445
529.3276
[M+Na]+
Observed
Acceptable = ± 3 mDa
Observed = -2.1 mDa
m/z
